# Supplementary material for: An Annotated Inventory of Tanzanian Medicinal Plants Traditionally Used for the Treatment of Respiratory Bacterial Infections
Source: Plants (Basel). 2022 Mar 30;11(7):931. doi: 10.3390/plants11070931 (PMC9002956; doi:10.3390/plants11070931)
Supplement: Supplementary file 1 [file plants-11-00931-s001.zip › plants-1626449-supplementary-update.pdf]

Table S1: Ethnobotanical information on medicinal plants against infective diseases of respiratory tract in Tanzania

Table S2: Anti-microbial activity of plants traditionally used against infective diseases of respiratory tract in Tanzania

Table S3: Natural products with antimicrobial activity from selected plants traditionally used against infective diseases of respiratory tract in Tanzania

Table S1: Ethnobotanical information on medicinal plants against infective diseases of respiratory tract in Tanzania (Keywords for searching: tuberculosis, TB, pneumonia, cough)

S. NO.	FAMILY	PLANT NAME	VOUCHER SPECIMEN	HABIT	PART USED	CLAIM FOR RESPIRATORY DISEASE RELATED CONDITION	OTHER CLAIMS FOR DISEASE CONDITION TREATMENT IN	PREPARATION	BOOK OR JOURNAL REFERENCE
1	Acanthaceae	*Acanthus pubescens* (Oliv.) Engl.	MJM 3028	shrub	leaves, root	coughs, pneumonia	treatment of warts, syphilis and gonorrhea	the leaves are put on fire to allow partial burning and then squeezed and one tablespoon full of the liquid is given to children to treat coughs	[63, 64]
			n.d.	n.d.	root	TB			[65]
2		*Adhatoda engleriana* (Lindau) C.B.Clarke	TMP: 185, 284	herb	root	TB	laxative, epilepsy		[66]
3	Achariaceae	*Caloncoba welwitschii* (Oliv.) Gilg	n.d.	tree	bark	pneumonia			[67]
4	Amaranthaceae	*Achyranthes aspera* L.	TMP: 103	herb	root	pneumonia		applied externally	[66, 67]
5		*Chenopodium ambrosioides* L. (syn. *Dysphania ambrosioides* (L.) Mosyakin & Clemants)	TMRU: 2972	herb	leaves	cough			[68, 69]
6	Anacardiaceae	*Lannea schimperi* (Hoechst. Ex A. Rich) Engl.	DK 047/06	tree or shrub	bark	TB	skin rashes, herpes zoster, herpes simplex, chronic diarrhea		[70]
7		*Lannea stuhlmannii* Engl. (syn. *Lannea schweinfurthii* var. *stuhlmannii* (Engl.) Kokwaro)	TMRU: 1822, 2448, 2992	tree	root	TB	stomachache, fever, asthma, dysentery	powdered root hairs taken with water against TB	[71]
8		*Mangifera indica* L.	DK 037/06	tree	leaves	TB			[70]

S. NO.	FAMILY	PLANT NAME	VOUCHER SPECIMEN	HABIT	PART USED	CLAIM FOR RESPIRATORY DISEASE RELATED CONDITION	OTHER CLAIMS FOR DISEASE CONDITION TREATMENT IN	PREPARATION	BOOK OR JOURNAL REFERENCE
			TMRU: 963	n.d.	leaves	cough	skin diseases, dysentery, asthma		[68, 71]
			n.d.	n.d.	bark	TB	waist pains, infertility, dysentery		[67]
			n.d.	n.d.	stem bark	TB			[65]
9		*Ozoroa insignis* Delile	DK 023/06	tree	root	TB	skin rashes, herpes simplex, herpes zoster, cryptococcal menengitis, oral candidiasis		[70]
10		*Ozoroa mucronata* (Bernh.) R. Fern & A. Fern.	TMRU: 580, 1966, 2389 TMRU: 580, 1966, 2389	tree	root, leaves	cough	fever, gonorrhoea, bilharzia, imotency	decoction of roots and leaves	[71]
11		*Pseudospondias microcarpa* (A. Rich.) Engl.	DK 005/06	shrub or tree	leaves, bark	TB	oral candidiasis		[70]
12		*Rhus natalensis* Bernh. Ex C. Krauss. (syn. *Searsia natalensis* (Bernh. Ex C.Krauss) F.A.Barkley)	TMP: 28	shrub	leaves	cough	colds, stomach pains		[66, 72]
13	Annonaceae	*Annona senegalensis* Pers.	TMRU: 1670, 2321, 2804	tree	root	pneumonia	cold, fever, chest pain, abdominal pain, vaginal prolapse, venral sores, menorrhalgia	decoction of roots	[71]
14		*Polyalthia stuhlmannii* (Engl.) Verdc.	TMRU: 308	shrub	root	cough	meningitis, rheumatic pain	decoction of roots	[71]
15		*Uvaria acuminata* Oliv.	TMRU: 1550, 1609, 1697	shrub	root	cough	abdominal pain, dysentery,	decoction of roots	[71]

S. NO.	FAMILY	PLANT NAME	VOUCHER SPECIMEN	HABIT	PART USED	CLAIM FOR RESPIRATORY DISEASE RELATED CONDITION	OTHER CLAIMS FOR DISEASE CONDITION TREATMENT IN	PREPARATION	BOOK OR JOURNAL REFERENCE
							menorrhalgia, snake bite, pectoral diseases		
16	Apocynaceae	*Diplorhynchus condylocarpon* (Müll. Arg.) Pichon.	TMRU: 70, 3048	tree	root	pneumonia	fever, hernia, frequent abortion and sterility in females, leprosy, gonorrhea	decoction of roots	[71]
			n.d.	n.d.	root	cough	abdominal pain	roots cooked with meat and the soup drunk	[73]
17		*Rauvolfia caffra* Sond.	n.d.	tree	root and stem barks	TB	antioxidant, antimicrobial	grind the stem barks and smell for headache. Root used for treatment of TB	[73]
			TMRU: 2980	tree	root	pneumonia	general body swellings, rheumatisms		[71, 72]
18	Araliaceae	*Stylochiton hennigii* Engl.	TMRU: 208	herb	root	bloody cough	constipation, hernia	decoction of roots given to children	[71]
19	Asparagaceae	*Asparagus africanus* Lam.	TMRU: 2323, 2340	herb	root	cough	sore throat		[72, 74]
20		*Asparagus setaceus* (Kunth) Jessop	TMRU: 1166	herb	fruits and leaves	pneumonia, cough			[71, 72]
21		*Dracaena steudneri* Engl.	DK 014/06	shrub or tree	bark	TB	cryptococcal meningitis, oral candidiasis		[70]
			n.d.	n.d.	stem	TB			[65]
22	Bignoniaceae	*Kigelia africana* (Lam.) Benth.	SMM-BD03	tree	stem bark	pneumonia	bilharzia, coughing, and female gynaecological problems, gonorrhea, dysentery	stem bark is boiled in water and drunk	[75]
			TMP: 52, (134), (136), 141	tree	stem bark	cough	expectorans		[66, 76]

S. NO.	FAMILY	PLANT NAME	VOUCHER SPECIMEN	HABIT	PART USED	CLAIM FOR RESPIRATORY DISEASE RELATED CONDITION	OTHER CLAIMS FOR DISEASE CONDITION TREATMENT IN	PREPARATION	BOOK OR JOURNAL REFERENCE
23		*Markhamia obtusifolia* (Baker) Sprague	TMP: 159, 164A	shrub	root	cough	scrofula, hookworm, snake bites	decoction of roots	[66, 77]
24		*Markhamia zanzibarica* (Bojer ex DC.) K. Schum.	TMRU: 382	tree	root	pneumonia	abdominal paint, psychiatric problems	decoction of roots	[71]
25		*Stereospermum kunthianum* Cham.	TMRU: 115	tree	root	cough	leprosy		[71, 77]
26	Boraginaceae	*Cordia 5ysmenor* Lam.	n.d.	tree	root	TB	cough, asthma		[78]
			n.d.	n.d.	root	TB			[65]
27		*Ehretia amoena* Klotzsch	TMRU: 1767	tree	root	TB, pneumonia	epilepsy, convulsions with fever, hookworm	decoction of roots	[71]
28		*Tecomaria capensis* (Thunb.) Spach.	n.d.	n.d.	leaves	pneumonia	bleeding gums, diarrhea, enteritis		[79]
29		*Trichodesma zeylanicum* (Burm.f.) R.Br.	TMP: 150	herb	root	TB	diuretic and against rheumatoid arthritis	decoction of roots	[66]
			n.d.	herb or shrub	stem bark	TB		powder in boiled water, oral; doze: 125 mL x 3 till cure	[78]
30	Canellaceae	*Warburgia salutaris* (Bertol.f.) Chiov.	n.d.	tree	bark	pneumonia	cough, haenia, typhoid fever, stomach ache		[67]
31		*Warburgia ugandensis* Sprague	n.d.	tree	n.d.	TB			[80]
			TMRU: 214	tree	leaves	TB	antidote in poisoning		[71]
			n.d.	tree	stem bark or root	TB, cough	asthma	stem bark boiled in water; one cup three times a day for TB and asthma; or soak powdered root in cold water, one table spoon three times a day till cure for cough	[78]

S. NO.	FAMILY	PLANT NAME	VOUCHER SPECIMEN	HABIT	PART USED	CLAIM FOR RESPIRATORY DISEASE RELATED CONDITION	OTHER CLAIMS FOR DISEASE CONDITION TREATMENT IN	PREPARATION	BOOK OR JOURNAL REFERENCE
			n.d.	n.d.	stem bark	TB			[65]
32	Cannabaceae	*Trema orientalis* (L.) Blume	TMRU: 1552	shrub	leaves	pneumonia, cough	bornichtis, antidote to general poisoning		[72, 81]
33	Capparaceae	*Boscia angustifolia* A. Rich	n.d.	shrub or tree	bark	cough		stem bark boiled in water; 1 teaspoon three times a day	[78]
34		*Cadaba farinosa* Forssk.	n.d.	tree	leaves	TB	ulcers, dysentery		[67]
35		*Capparis erythrocarpos* Isert	DK 028/06	shrub	root	TB	skin rashes, cryptococcal meningitis, oral candidiasis, herpes zoster, herpes simplex, chronic diarrhea		[70]
36		*Capparis tomentosa* Lam.	DK 020/06	shrub	root	TB	oral candidiasis, herpes zoster, herpes simplex		[70]
			TMRU: 2780	tree or shrub	root	pneumonia, cough	impotency, barrenness, wounds, ophthalmic problems, leprosy		[68, 69]
37	Celastraceae	*Elaeodendron buchananii* (Loes.) Loes. (syn. *Cassine buchananii* Loes.)	TMRU: 187	tree or shrub	root	coughing blood	wounds, syphilis		[69, 72]
38		*Maytenus senegalensis* (Lam.) Exell. (syn. *Gymnosporia senegalensis* (Lam.) Loes.)	DK 018/06	shrub or tree	bark/root	TB	herpes simplex, herpes zoster, oral candidiasis, skin rashes		[70]
			TMRU: 11	shrub or tree	root	pneumonia		root decoction	[69]
39	Chrysobalanaceae	*Parinari curatellifolia* Planch. Ex Benth.	DK 039/06	tree	bark/root	TB	skin rashes, chronic diarrhea, herpes zoster, herpes simplex		[70]

S. NO.	FAMILY	PLANT NAME	VOUCHER SPECIMEN	HABIT	PART USED	CLAIM FOR RESPIRATORY DISEASE RELATED CONDITION	OTHER CLAIMS FOR DISEASE CONDITION TREATMENT IN	PREPARATION	BOOK OR JOURNAL REFERENCE
40	Clusiaceae	*Garcinia buchananii* Baker	DK 063/06	tree	bark/root	TB	chronic diarrhoea, cryptococcal meningitis, herpes zoster, herpes simplex, skin rashes		[70]
			n.d.	n.d.	root bark	TB			[65]
41	Combretaceae	*Combretum collinum* Fresen.	DK 041/06	tree	leaves/bark/ root	TB	chronic diarrhea		[70]
42		*Combretum fragrans* F. Hoffm. (syn. *Combretum adenogonium* Steud. Ex A.Rich.)	TMRU: 2792	tree	root	cough	syphilis		[69, 72]
43		*Combretum zeyheri* Sond.	TMP: 210	tree	leaves	cough		dried leaves smoked	[66, 72]
44		*Terminalia kilimandscharica* Engl.	TMP: 151	tree	stem bark	cough	asthma, pains in back and loins	decoction of stem bark drunk	[66]
45	Compositae (Asteraceae)	*Ageratum conyzoides* (L.) L.	MJM 3023	herb	root, leaves	cough	constipation, peptic ulcers and fibroids, women with difficulties to conceive	roots are chewed fresh as an anti-acid and antiseptic; leaves are boiled and decoction taken as tea	[63]
			TMRU: 3182	herb	root	cough	chest complaints		[69, 72]
46		*Crassocephalum mannii* (Hook.f.) Milne-Redh. (syn.: *Solanecio mannii* (Hook.f.) C. Jeffrey)	n.d.	shrub or small tree	root	TB	asthma	boil roots with a soup of sheep; Used fresh, 1 cup x 3; very short shelf life of 3 days	[78]
47		*Microglossa pyrifolia* (Lam.) Kunze	MJM 3200	shrub	leaves	cough	cleansing airways, colds, flu	leaves are pounded and the sap squeezed into the nostrils	[63]

S. NO.	FAMILY	PLANT NAME	VOUCHER SPECIMEN	HABIT	PART USED	CLAIM FOR RESPIRATORY DISEASE RELATED CONDITION	OTHER CLAIMS FOR DISEASE CONDITION TREATMENT IN	PREPARATION	BOOK OR JOURNAL REFERENCE
			TMP: 275	trailing sub-shrub	entire plant	chronic cough with fever	stomach pains, headache, pain in chest and shoulders with chronic rhinitis, for heartburn, epilepsy, hookoworm, furunculosis, impotence		[66, 68]
48		*Senecio discifolius* Oliv. (syn. *Emilia discifolia* (Oliv.) C.Jeffrey)	DK 058/06	tree	bark	TB	cryptococcal meningitis		[70]
49		*Senecio stuhlmannii* Klatt (syn. *Solanecio cydoniifolius* (O.Hoffm.) C.Jeffrey)	MJM 3043/3134	climbing herb	leaves	cough	wounds, swellings, stiff neck, poisoning	for coughs the leaves are baked with salt and chewed.	[63]
50		*Vernonia adoensis* Sch. Bip. Ex Walp (syn. *Baccharoides adoensis* (Sch.Bip. ex Walp.) H.Rob.)	DK 008/06	herb	leaves	TB			[70]
			n.d.	herb	root	TB	digestive/appetizer, stomach pain, blotches, gonorrhea		[82, 83]
51		*Vernonia 8ysmenor* (L.) Less (syn. *Cyanthillium cinereum* (L.) H.Rob.)	n.d.	herb	root and leaves	TB	febrifuge, vermifuge, malaria, incontinence, pile, cough, pneumonia, asthma, bronchitis, snake-bite		[82, 83]
52		*Vernonia colorata* (Willd.) Drake (syn. *Gymnanthemum coloratum* (Willd.) H.Rob. & B.Kahn)	TMP: 95	shrub	root	cough	emetic, antifebrile		[66, 68]
53			TMP: 143	shrub	leaves	cough		infusion	[66, 72]

S. NO.	FAMILY	PLANT NAME	VOUCHER SPECIMEN	HABIT	PART USED	CLAIM FOR RESPIRATORY DISEASE RELATED CONDITION	OTHER CLAIMS FOR DISEASE CONDITION TREATMENT IN	PREPARATION	BOOK OR JOURNAL REFERENCE
		Vernonia hildebrandtii Vatke	n.d.	n.d.	leaves, stem	cough	strangulated hernia, stomach troubles		[79]
54		*Vernonia zanzibarensis* Less.	TMRU: 935	shrub	leaves	cough			[69, 72]
55	Convolvulaceae	*Ipomoea sinensis* (Desr.) Choisy (syn. *Ipomoea biflora* (L.) Pers.)	DK 055/06	herb	leaves	TB	oral candidiasis		[70]
56	Costaceae	*Costus afer* Ker Gawl.	TMRU: 1148	herb	leaves	whooping cough		the leaves are crushed, then cooked in sugar-cane juice and the liquid is drunk.	[81]
57	Crassulaceae	*Bryophyllum pinnatum* (Lam.) Oken	MJM 3107	herb	leaves	cough	cold/flu	the succulent leaves are wrapped in banana leaves and baked under fire; the baked leaves are then squeezed and the exuding juice administered to the patients orally	[63]
58	Dennstaedtiaceae	*Pteridium aquilinum* (L.) Kuhn	DK 029/06	fern	leaves	TB	oral candidiasis		[70]
59	Ebenaceae	*Diospyros fischeri* Gürke	n.d.	tree	leaves	cough	asthma	soak pound leaves in fresh cold water; one cup three times per day till cured	[78]
60		*Diospyros mespiliformis* Hochst. Ex DC.	n.d.	n.d.	leaves	cough	anthelmintic, wounds and sores, leprosy, dysentery		[79]

S. NO.	FAMILY	PLANT NAME	VOUCHER SPECIMEN	HABIT	PART USED	CLAIM FOR RESPIRATORY DISEASE RELATED CONDITION	OTHER CLAIMS FOR DISEASE CONDITION TREATMENT IN	PREPARATION	BOOK OR JOURNAL REFERENCE
61		*Euclea divinorum* Hiern	TMP: 229	shrub	root	pneumonia	chest pains, internal body swellings	a decoction of the roots mixed with roots of *Croton megalocarpus*	[84]
62	Euphorbiaceae	*Acalypha fruticosa* Forssk.	TMRU: 1201, 1835	shrub	root	cough	stomach problems		[72, 85]
			n.d.	n.d.	leaves	cough	chest pains, stomach aches, cholera		[68, 74]
			n.d.	n.d.	leaves	cough	cholera, stomach ache, chest pains		[79]
63		*Croton dichogamus* Pax	TMP: 294	shrub or tree	root	TB		powdered root in porridge or tea	[84]
			n.d.	n.d.	root	TB			[65]
64		*Euphorbia heterochroma* Pax	TMP: 242	tree	root and stems	TB, cough		a decoction of the fresh roots	[84]
65		*Euphorbia hirta* L.	n.d.	n.d.	plant	cough	gonorrhea, dysentery, boils, ophthalmic, wounds		[79]
67		*Jatropha curcas* L.	TMRU: 2935	shrub or tree	root	pneumonia	abortivum, anthelmintic, laxative and syphilis	root decoction	[74, 86]
68		*Mallotus oppositifolius* (Geiseler) Müll. Arg.	TMRU: 1116	tree or shrub	leaves	pneumonia			[74, 77]
69		*Ricinus communis* L.	DK 048/06	herb	root	chronic cough			[70]
70		*Sapium ellipticum* (Hochst.) Pax (syn. *Shirakiopsis elliptica* (Hochst.) Esser)	DK 019/06	shrub or tree	bark	TB	herpes zoster, cryptococcal meningitis		[70]
71		*Spirostachys africana* Sond.	TMRU: 1495, 1630, 2379	tree or shrub	root bark	cough	diarrhea, bilharzia	rootbark decoction	[74]
72		*Suregada zanzibariensis* Baill.	TMRU: 1980, 2117, 2367	shrub	root	pneumonia	stomach aches, hernia, chest pains, malaria,	root decoction	[74]

S. NO.	FAMILY	PLANT NAME	VOUCHER SPECIMEN	HABIT	PART USED	CLAIM FOR RESPIRATORY DISEASE RELATED CONDITION	OTHER CLAIMS FOR DISEASE CONDITION TREATMENT IN	PREPARATION	BOOK OR JOURNAL REFERENCE
							schistsosomiasis, chickenpox, gonorrhea		
73		*Synadenium glaucescens* Pax. (syn. *Euphorbia neoglaucescens* Bruyns)	TMP: 194	shrub	root	TB, severe cough		cold water extract of the debarked root with sugar after standing for 3 days	[84]
74	Lamiaceae (Labiatae)	*Coleus barbatus* (Andrews) Benth. Ex G. Don (syn. *Plectranthus barbatus* Andrews, syn. *Plectranthus comosus* Sims)	TMP: 164 (223)	shrub	leaves	cough	tonsillitits	leaves are pounded and drunk with water	[84]
			DK 071/06	shrub	leaves	TB	herpes zoster, herpes simplex, skin rashes, oral candidiasis		[70]
75		*Hoslundia opposita* Vahl	TMRU: 109, 238, 285, 1013	shrub	root	cough			[72, 74]
			TMP: 2, 7, 17, 24, 46, 51, 60, 63, 108, 128, 213	shrub	root	cough		decoction of roots	[84]
			n.d.	n.d.	entire plant	cough	gonorrhoea, cystitis, blenorrhoea, liver disease, pains in the chest, fever, hookworm, stomach disorders, wounds, mental disturbance		[68, 84]
			n.d.	shrub	root	cough	asthma	powdered roots boil in water; 1 teaspoon three times a day	[78]

S. NO.	FAMILY	PLANT NAME	VOUCHER SPECIMEN	HABIT	PART USED	CLAIM FOR RESPIRATORY DISEASE RELATED CONDITION	OTHER CLAIMS FOR DISEASE CONDITION TREATMENT IN	PREPARATION	BOOK OR JOURNAL REFERENCE
			n.d.	n.d.	plant	cough	gonorrhea, cystitis, wounds, liver disease, blenorrhoea, hookworms		[79]
76		*Hyptis pectinata* (L.) Poit.	TMRU: 2877	herb	leaves	cough	roundworm, fever		[68, 74]
77		*Ocimum suave* Willd. (syn: *Ocimum gratissimum* L.)	TMP: 77	shrub	leaves	cough	abdominal pains, sore eyes, ear troubles		[72, 84]
78		*Vitex fischeri* Gürke	DK 068/08	shrub	bark	TB	herpes zoster		[70]
79	Lauraceae	*Ocotea usambarensis* Engl.	n.d.	tree	bark	coughing	stomach gas, roundworms	infusion	[87]
80	Leguminosae (Fabaceae)	*Abrus precatorius* L.	TMRU: 2801, 2827	shrub, prostrate or climbing	leaves	cough	fever and dizziness	leaves are crushed and the juice drunk	[85]
			TMP: 49, 61	shrub, prostrate or climbing	leaves	cough	conjunctivitis, stomach troubles	decoction of the leaves	[88, 89]
81		*Acacia brevispica* Harms	n.d.	shrub	root bark	TB, cough		decoction of *A. brevispica* (root bark) + *W. ugadensis* (stem bark) + *C. febrifuga* (root bark) + Lusunga root in hot water. 1 spoon full + 3 daily till recovery	[78]
			TMRU: 28	tree or shrub	root	cough		the root decoction is drunk	[85]
82		*Acacia bussei* Sjostedt	TMP: 289	tree	bark	cough		a decoction of the bark is drunk	[72, 84]

S. NO.	FAMILY	PLANT NAME	VOUCHER SPECIMEN	HABIT	PART USED	CLAIM FOR RESPIRATORY DISEASE RELATED CONDITION	OTHER CLAIMS FOR DISEASE CONDITION TREATMENT IN	PREPARATION	BOOK OR JOURNAL REFERENCE
83		*Acacia mellifera* (M. Vahl) Benth.	TMP: 220	tree	bark	pneumonia	stomach trouble, sterility, malaria, syphilis	the bark is boiled in water and the liquid used as a remedy	[72, 84]
			n.d.	n.d.	bark	pneumonia	Syphilis, malaria, sterility, stomachache		[79]
84		*Acacia nilotica* (L.) Delile	TMRU: 2133, 2345	tree	root	TB			[68, 85]
			TMRU: 2133, 2345	tree	leaves	pneumonia			[72, 85]
			TMRU: 2133, 2345	tree	stem bark	cough	fever		[72, 85]
			n.d.	n.d.	plant	TB, pneumonia	gonorrhea, diarrhea, smallpox		[79]
85		*Acacia polyacantha* subsp. *Campylacantha* (A. Rich.) Brenan	TMP: 202	shrub	n.d.	pneumonia	throat maladies, tooth troubles, leprosy, gonorrhea		[68, 84]
			TMRU: 1428	tree	root	whooping cough		root infusion	[85]
86		*Afrormosia angolensis* (Baker) de Wild. (syn. *Pericopsis angolensis* (Baker) Meeuwen)	n.d.	tree	root bark	TB		powder in hot water, 1 tea spoon x 3	[78]
87		*Afzelia quanzensis* Welw.	TMRU: 1222, 1256	tree	stem bark	pneumonia	antimalarial		[71, 77]
88		*Albizia anthelmintica* Brongn.	n.d.	shrub or tree	bark	cough	asthma	powdered bark boil in water; one cup four times a day	[78]

S. NO.	FAMILY	PLANT NAME	VOUCHER SPECIMEN	HABIT	PART USED	CLAIM FOR RESPIRATORY DISEASE RELATED CONDITION	OTHER CLAIMS FOR DISEASE CONDITION TREATMENT IN	PREPARATION	BOOK OR JOURNAL REFERENCE
89		*Albizia versicolor* Oliv.	TMRU: 3211	tree	stem bark	cough		powdered dried stembark is sniffed to relieve cough	[85]
			TMP: 47	tree	stem bark	cough		the cold-water extract is beaten to produce foam and eaten	[77, 89]
			n.d.	tree	root	cough		grind and cook root with a lizard called *gemwambuli*, 1 teaspoon three times a day	[78]
			n.d.	n.d.	leaves	TB			[65]
90		*Bauhinia reticulata* DC.	n.d.	n.d.	plant	cough	dysentery, leprosy, roundworms, anthrax, malaria		[79]
91		*Cajanus cajan* (L.) Millsp.	n.d.	herb	leaves	cough		dry leaves in hot water, one teaspoon three times a day	[78]
92		*Cassia abbreviata* subsp. *Beareana* (Holmes) Brenan	TMP: 163	tree	root	pneumonia	chest complaints, uterine complaints, stomach troubles and fever of malaria, suspected syphilis, gonorrhea	decoction of roots	[66, 72]
		Cassia abbreviata Oliv.	n.d.	n.d.	dry root	pneumonia	gonorrhea, syphilis, diarrhea, dysentery, malaria		[79]
93		*Cassia mimosoides* L. (syn. *Chamaecrista mimosoides* (L.) Greene)	DK 0024/06	herb	leaves/root	TB			[70]

S. NO.	FAMILY	PLANT NAME	VOUCHER SPECIMEN	HABIT	PART USED	CLAIM FOR RESPIRATORY DISEASE RELATED CONDITION	OTHER CLAIMS FOR DISEASE CONDITION TREATMENT IN	PREPARATION	BOOK OR JOURNAL REFERENCE
94		*Cassia singueana* Delile (syn. *Senna singueana* (Delile) Lock)	TMP: 53	shrub	leaves	pneumonia	bronchopneumonia	crushed leaves are drunk	[66]
95		*Dichrostachys 15ysmenor* (L.) Wight & Arn.	n.d.	tree	leaves, stem and root bark	TB	antidiarrheal, antioxidant, nephroprotective, immunostimulant, antibacterial	grind the leaves and dress the wounds. Roots decoction used for TB, infertility, veneral diseases, abdominal ulcers	[73]
			n.d.	tree	root	TB	chronic ailments, infertility, veneral diseases		[67]
			TMP: 25, 43, 92	tree	root	TB		the smoke of the root and/or leaf is inhaled to treat pulmonary TB	[68, 84]
		Dichrostachys 15ysmenor (L.) Wight et Arn. Subsp. *15ysmenor* Brenan & Brummitt	TMP: 25, 43, 92	tree or shrub	leaves	pneumonia		leaf juice is drunk	[77, 84]
			TMRU: 1680, 1953	tree or shrub	root	pneumonia, persistent cough	burning abdominal pains, menorrhagia, bloody diarrhoea, threatened abortion	decoction	[85]
		Dichrostachys cinerea (L.) Wight & Arn. (syn. *Dichrostachys glomerata* (Forssk.) Chiov.)	n.d.	tree or shrub	root bark	TB	asthma	boil mixed root powder of *D. glomerata* and *Albizia versicolor* + cow liver	[78]
96		*Entada abyssinica* A. Rich.	TMRU: 3132	tree	root bark	TB, cough	catarrh and asthma	powder in boiled water, oral; 125 mL three times a day till cured	[77, 85]

S. NO.	FAMILY	PLANT NAME	VOUCHER SPECIMEN	HABIT	PART USED	CLAIM FOR RESPIRATORY DISEASE RELATED CONDITION	OTHER CLAIMS FOR DISEASE CONDITION TREATMENT IN	PREPARATION	BOOK OR JOURNAL REFERENCE
			DK 026/06	shrub or tree	leaves/bark	TB	skin rashes, oral candidiasis, herpes zoster, herpes simplex		[70, 80]
			n.d.	n.d.	root	TB			[65, 70]
97		*Entada leptostachya* Harms	DK 043/06	climbing shrub or tree	root	TB	skin rashes, herpes simplex, herpes zoster		
98		*Erythrina abyssinica* DC.	DK 040/06	tree	bark/root	TB			[70]
			n.d.		root bark	TB			[65]
99		*Lonchocarpus bussei* Harms	TMRU: 1792, 2998	tree	root	cough	diuretic		[72, 85]
			n.d.	n.d.	bark	cough	gonorrhoea		[79]
100		*Peltophorum africanum* Sond.	n.d.	tree	n.d.	TB	HIV, wounds, toothache, sore throat, cough, abdominal disorders, diarrhea, menorrhagia, infertility, dysentery		[90]
101		*Tamarindus indica* L.	TMP: 155	tree	root	cough	fevers	decoction of roots	[66, 72]
102		*Tephrosia purpurea* subsp. *Leptostachya* (DC.) Brummit	TMRU:1654	herb	root	cough	tightness of the chest, as a cholagogue, diuretic, asthma, deobstruent, tonic and laxative		[85, 91]
			n.d.	n.d.	plant	bilious cough	biliary and splenic troubles		[85, 92]
103	Loganiaceae	*Strychnos spinosa* Lam.	TMP: 198	tree	stem bark	cough	stomach trouble	powdered bark	[84, 88]
104	Malvaceae	*Adansonia 16ysmenor* L.	n.d.	tree	bark	TB			[67]
105		*Dombeya cincinnata* K. Schum. (syn. *Dombeya acutangula* Cav.)	TMRU: 169, 181, 1724	tree	root	cough with chest pains	against vomiting, abdominal pains, to facilitate conception	the root decoction is drunk	[81]

S. NO.	FAMILY	PLANT NAME	VOUCHER SPECIMEN	HABIT	PART USED	CLAIM FOR RESPIRATORY DISEASE RELATED CONDITION	OTHER CLAIMS FOR DISEASE CONDITION TREATMENT IN	PREPARATION	BOOK OR JOURNAL REFERENCE
106		*Grewia bicolor* Juss. (syn. *Grewia damine* Gaertn.)	TMRU: 572	tree or shrub	root	cough	mental illness, hernia, female sterility, convulsions, vaginal and rectal prolapse	the root decoction is drunk	[81]
107		*Grewia microcarpa* K. Schum.	TMRU: 1703	shrub	root, leaves	cough		the root decoction or the leaf juice is drunk.	[81]
108		*Hibiscus micranthus* L.f.	TMRU: 2020, 3137	shrub	root	pneumonia		root decoction	[74]
109		*Hibiscus surattensis* L.	TMRU: 926	herb	leaves	severe cough		the leaves are crushed and the juice drunk	[74]
110		*Pavonia urens* Cav. JAM 1855	n.d.	herb	root	pneumonia	stomachache	honey-based poultice	[87]
111		*Sida rhombifolia* L.	TMP: 100	herb	n.d.	TB	rheumatism	an extract or an infusion of the plant	[68, 84]
112		*Sida serratifolia* R. Wilczek & Steyaert	n.d.	n.d.	leaves	pulmoraly TB	diarrhoea		[79]
113		*Waltheria indica* L.	TMP: 74	tree	root	cough		decoction of the roots	[72, 89]
			n.d.	n.d.	n.d.	cough	abortifacient, emolient		[68, 89]
114	Meliaceae	*Trichilia emetica* Vahl	MJM 3066	tree	stem bark, leaves, root	TB	epilepsy, malaria, syphilis, gonorrhea	stem bark/leaves are boiled in water and decoction administered. In the treatment of gonorrhea and syphilis the roots are boiled together with the roots of *Alchonea cordifolia, Sapium ellipticum, Zenheria scabra, Acanthus pubescens, Emilia javanica*	[63]

S. NO.	FAMILY	PLANT NAME	VOUCHER SPECIMEN	HABIT	PART USED	CLAIM FOR RESPIRATORY DISEASE RELATED CONDITION	OTHER CLAIMS FOR DISEASE CONDITION TREATMENT IN	PREPARATION	BOOK OR JOURNAL REFERENCE
								and *Oxygonum sinuatum* and the decoction drunk.	
			n.d.	tree	leaves, bark	TB		fresh leaves boiled in water, 1 cup x 3 for TB, bark powder soaked in hot water for cough	[78]
			TMRU: 3009	tree	stem bark, root bark	whooping cough	boils	decoction of stembark and rootbark is drunk	[74]
			n.d.	tree	bark	pneumonia	jigger infections		[72, 74]
115	Menispermaceae	*Cissampelos pareira* L. var. *orbiculata* (L.) Miq. (syn. *Cissampelos pareira* L.)	TMP: 30 (101)	vine	root	cough	sore throat, cold, snake bite	roots are powdered and soak in water	[72, 84]
116	Monimiaceae	*Xymalos monospora* (Harv.) Baill.	MJM 3040	tree	leaves	cough		leaves are pounded together with ginger to make a thick paste or squeezed and the juice produced given to children for coughs	[63]
117	Moraceae	*Milicia excelsa* (Welw.) C.C. Berg	n.d.	tree	bark	pneumonia	infants fever, uterus cancer		[67]

S. NO.	FAMILY	PLANT NAME	VOUCHER SPECIMEN	HABIT	PART USED	CLAIM FOR RESPIRATORY DISEASE RELATED CONDITION	OTHER CLAIMS FOR DISEASE CONDITION TREATMENT IN	PREPARATION	BOOK OR JOURNAL REFERENCE
118	Moringaceae	*Moringa oleifera* Lam.	n.d.	tree	leaves	TB		fresh leaves boiled + lime juice + *Piper nigrum* + table salt; 1 cup x 3	[78]
119	Myricaceae	*Myrica salicifolia* Hochst. Ex A. Rich.	DK 057/06	shrub or tree	bark/root	TB	chronic diarrhea, cryptococcal meningitis, herpes simplex		[70]
			n.d.	n.d.	stem bark	cough		the powdered, dry stembark is mixed with honey and eaten	[84]
120	Myrtaceae	*Eucalyptus spp.*	n.d.	tree	n.d.	TB			[80]
			n.d.	n.d.	leaves	TB			[65]
121		*Psidium guajava* L.	DK 042/06	shrub or tree	leaves	TB	chronic diarrhea		[70]
			TMRU 3184	shrub or tree	leaves	cough and pulmonary disorders	sprains, diarrhea, intestinal haemorrhages		[68, 93]
			n.d.	n.d.	leaves	TB			[65]
122		*Syzygium cordatum* Hochst. Ex Krauss	TMRU 3165, 3166	tree	plant	TB	emetic		[68, 93]
123	Phyllanthaceae	*Antidesma membranaceum* Müll.Arg.	TMRU: 3108	tree or shrub	root	pneumonia	kwasnhiorkor, mouth ulcers		[70, 72]
124		*Antidesma venosum* E.Mey ex Tul.	DK 049/06	shrub or tree	root	TB	chronic diarrhoea, oral candidiasis		[70]
			TMRU: 978, 2309, 2904	tree or shrub	root	purulent cough in children	uterine prolapse, abdominal pains, dysmenorrhea, gonorrhoea, bilharzia, malaria	the root decoction is drunk	[81]
125			n.d.	tree	inner bark	cough		chew inner bark with salt	[78]

S. NO.	FAMILY	PLANT NAME	VOUCHER SPECIMEN	HABIT	PART USED	CLAIM FOR RESPIRATORY DISEASE RELATED CONDITION	OTHER CLAIMS FOR DISEASE CONDITION TREATMENT IN	PREPARATION	BOOK OR JOURNAL REFERENCE
		Bridelia micrantha (Hochst.) Baill.	n.d.	tree	root	TB	women's stomach ache, infertility		[67, 94]
126		*Flueggea virosa* (Roxb. Ex Willd.) Royle (syn. *Securinega virosa* (Roxb. Ex Willd.) Baill.)	TMRU: 272, 1003	tree or shrub	root	pneumonia	diarrhea		[68, 74]
			TMP: 14, 79, 105	shrub	stem bark	pneumonia	diarrhea		[68, 84]
			n.d.	n.d.	pulp	pneumonia	diarrhoea, gonorrhoea		[79]
127		*Margaritaria discoidea* (Baill.) G. L. Webster	TMRU: 1948	tree	root	cough	polymenorrhoea		[74]
128		*Phyllanthus amarus* Schumach. & Thonn.	TMRU: 3156	herb	aerial parts and leaves	persistent cough		infusion of the aerial parts is drunk and the leaves are chewed	[93]
129		*Pseudolachnostylis maprouneifolia* Pax	TMRU: 121, 1944	tree	stem bark	pneumonia	anaemia	dried stembark added to porridge	[68, 74]
130	Polygalaceae	*Securidaca longipedunculata* Fresen.	TMP: 119, 265	tree	root	cough	chest complains	decoction of roots	[89]
			n.d.	n.d.	leaves	cough		a decoction of the very young leaf made with lokal salt of vegetable origin	[68, 89]
			n.d.	tree	root bark	cough	asthma	powdered root boil in water, one teaspoon three times a day	[78]
131	Polygonaceae	*Rumex usambarensis* (Dammer) Dammer	TMRU: 3286	shrub	leaves	cough	stomach pains		[72, 95]
132	Polypodiaceae	*Phymatodes scolopendria* (Burm. F.) Ching (syn. *Phymatosorus scolopendria* (Burm. F.) Pic. Serm.)	TMRU: 2553	rhizomatus fern	leaves	pneumonia	chickenpox, oedema, enlarged spleen	decoction	[71, 77]

S. NO.	FAMILY	PLANT NAME	VOUCHER SPECIMEN	HABIT	PART USED	CLAIM FOR RESPIRATORY DISEASE RELATED CONDITION	OTHER CLAIMS FOR DISEASE CONDITION TREATMENT IN	PREPARATION	BOOK OR JOURNAL REFERENCE
133	Ranunculaceae	*Clematis brachiata* Thunb.	TMRU: 3106	woody climber	root	cough	syphilis, thrush, headaches		[68, 95]
134		*Clematis hirsuta* Guill. & Perr.	DK 051/06	herb	leaves	TB	cryptococcal meningitis, herpes zoster		[70]
135	Rhamnaceae	*Rhamnus prinoides* L'Hér.	TMRU: 3004	tree or shrub	root	pneumonia			[68, 95]
136		*Scutia myrtina* (Burm. F.) Kurz	TMP: 195	shrub	stem bark	cough	emetic, chest troubles	decoction of stem bark	[68, 89]
137		*Ziziphus abyssinica* Hochst. Ex A. Rich.	n.d.	shrub	root	pneumonia		root powder is given into skin incisions	[86]
138		*Ziziphus pubescens* Oliv.	n.d.	n.d.	leaves	pneumonia	diarrhoea, dysentery, wounds, skin diseases		[79]
139	Rubiaceae	*Agathisanthemum bojeri* Klotzsch	TMRU: 1621, 2140	herb	seeds	cough	chest problems		[68, 95]
140		*Chassalia violacea* K.Schum.	n.d.	shrub	root	pneumonia	infants complications, mental case, infertility, syphilis		[67]
141		*Crossopteryx febrifuga* (Afzel. Ex G. Don) Benth.	TMP: 162,269	tree	root	TB, cough	spasm of the stomach	decoction of the roots	[77, 89]
			SMM-BD11	tree	root bark	TB	venereal diseases	root bark is boiled in water, the decoction taken orally	[75]
			n.d.	tree	root bark	TB		root powder of *C. febrifuga* and *Cordia africana* are soaked in hot water; 1 table spoon x 3 till cure	[78]
			n.d.	n.d.	root	TB			[65]

S. NO.	FAMILY	PLANT NAME	VOUCHER SPECIMEN	HABIT	PART USED	CLAIM FOR RESPIRATORY DISEASE RELATED CONDITION	OTHER CLAIMS FOR DISEASE CONDITION TREATMENT IN	PREPARATION	BOOK OR JOURNAL REFERENCE
142		*Kraussia kirkii* (Hook. F.) Bullock	TMRU: 1293	shrub	root	cough		the dried powdered roots together with the dried powdered rootbark of *Carica papaya* L. are mixed with honey and taken internally. Pregnant women are prohibited from taking this remedy.	[95]
143		*Pavetta crassipes* K. Schum.	TMRU: 2936	tree or shrub	root and leaves	TB and cough	gonorrhea	grind dry/fresh roots + chicken soup	[68, 78, 95]
			n.d.	n.d.	root	TB			[65]
144		*Rubia cordifolia* L.	n.d.	herb	root	cough			[78]
			n.d.	n.d.	whole plant	TB			[65]
145		*Tarenna littoralis* (Hiern) Bridson (syn. *Coptosperma littorale* (Hiern) Degreef)	TMRU: 551, 1395	tree or shrub	root	cough with chest pains	amenorrhoea	the root decoction is drunk	[93]
146		*Vangueria infausta* subsp. *Rotundata* (Robyns) Verdc. (syn. *Vangueria rotundata* Robyns)	TMP: 70, 96, 246	shrub	root	pneumonia, cough	malarial fever, roundworms, purgative		[68, 89]
147	Rutaceae	*Citrus limon* (L.) Osbeck	DK 075/06	tree	root	TB			[70]
148		*Fagara chalybaea* Engl. (Engl.)	n.d.	n.d.	root bark	cough	Diarrhea, malaria, toothache		[79]

S. NO.	FAMILY	PLANT NAME	VOUCHER SPECIMEN	HABIT	PART USED	CLAIM FOR RESPIRATORY DISEASE RELATED CONDITION	OTHER CLAIMS FOR DISEASE CONDITION TREATMENT IN	PREPARATION	BOOK OR JOURNAL REFERENCE
149		*Gardenia ternifolia* subsp. *Jovis-tonantis* (Welw.) Verdc.	TMRU: 1509, 1672, 2257	tree or shrub	root	cough	malaria, laxative		[77, 95]
150		*Harrisonia abyssinica* Oliv	TMP: 106, 129	shrub or tree	root	TB			[89]
151		*Teclea simplicifolia* (Engl.) I. Ver. (syn. *Vepris simplicifolia* (Engl.) Mziray)	TMRU: 430	tree	root	pneumonia			[68, 95]
152		*Toddalia asiatica* (L.) Lam.	TMRU: 2990	shrub	leaves	pneumonia	malaria, intermittent fever, lung diseases, toothaches, rheumatic fever, syphilis, cholera		[68, 95]
			n.d.	n.d.	root	cough	emetic, snake bites		[72, 95]
			n.d.	n.d.	fruits	cough	colds		[72, 95]
153		*Vepris eugeniifolia* (Engl) I. Verd.	TMP: 217	tree	leaves	pneumonia	lung diseases	decoction of the leaf is administered with milk	[68, 86, 89]
154		*Zanthoxylum chalybeum* Engl.	n.d.	shrub or tree	root	TB; pneumonia		root decoction or pulp	
			TMP: 40, 50, 75, 209, 278	shrub	root	pneumonia	hookworm	decoction of roots	[77, 89]
			TMRU: 1675	shrub or tree	root	whooping cough	stomachache, strangulated hernia, schistosomiasis, skin diseases, hyper- and dysmenorrhoea, female infertility, swellings	root decoction	[95]
			TMRU: 1675	tree	stem bark	cough	malaria, colds, dizziness		[72, 95]

S. NO.	FAMILY	PLANT NAME	VOUCHER SPECIMEN	HABIT	PART USED	CLAIM FOR RESPIRATORY DISEASE RELATED CONDITION	OTHER CLAIMS FOR DISEASE CONDITION TREATMENT IN	PREPARATION	BOOK OR JOURNAL REFERENCE
			n.d.	tree	root bark	cough	asthma	powder in cold water, or powdered root in hot water; one teaspoon three times a day	[78]
			n.d.	n.d.	root	TB			[65]
155	Salicaeae	*Dovyalis abyssinica* (A. Rich.) Warb.	MJM 3033	tree	stem bark	cough		the bark is boiled in water together with the leaves of *Abrus precarorius* and the decoction drunk	[63]
156	Sapindaceae	*Allophylus ferrugineus* Taub.	n.d.	tree or shrub	root	coughing	stomach gas, roundworms, fever	infusion	[87]
157		*Deinbollia borbonica* Scheff.	TMRU: 2140, 2961	tree or shrub	root	cough	mental illness, hiccup, hernia, headaches, bilaharzia, gonorrhoea, stomachaches, malaria, convulsions, poliomyelitis, cardiac pains	roto decoction	[95]
158		*Zanha africana* (Radlk.) Exell	n.d.	tree	leaves	TB			[67]
			n.d.	shrub or tree	bark	cough	asthma	lick powder twice a day	[78]
159	Sapotaceae	*Pachystela brevipes* (Baker) Baill. (syn. *Synsepalum brevipes* (Baker) T.D.Penn.)	TMRU: 1134	tree	root	cough	malaria, catarrh, aphrodisiac		[77, 81]
160		*Sideroxylon inerme* L.	TMRU: 474	tree or shrub	root	cough	conjunctivitis, hernia, paralysis		[81]

S. NO.	FAMILY	PLANT NAME	VOUCHER SPECIMEN	HABIT	PART USED	CLAIM FOR RESPIRATORY DISEASE RELATED CONDITION	OTHER CLAIMS FOR DISEASE CONDITION TREATMENT IN	PREPARATION	BOOK OR JOURNAL REFERENCE
161		*Synsepalum cerasiferum* (Welw.) T.D.Penn.	MJM 3077/3097	tree	stem bark	TB	malaria, veneral diseases, and urinary tract infections	the stem bark is boiled in water and decoction taken orally	[63]
162	Smilacaceae	*Smilax anceps* Willd. (syn. *Smilax kraussiana* Meisn.)	n.d.	herb	leaves	TB			[67]
			TMRU: 3117	climbing shrub	root	cough	veneral diseases, diuretic		[68, 81]
163	Solanaceae	*Solanum incanum* L.	TMP: 102	shrub	n.d.	pneumonia	pleurisy	roasted parts of the plant	[89]
			TMP: 102	shrub	root<br>leaves	cough	abdominal pains, liver troubles, carbuncle, colic, sore throat, gonorrhoea, syphilis<br>snake bite, colic, sore throat, gonorrhoea, syphilis		[68, 89]
			n.d.	n.d.	fruits	TB			[65]
			n.d.	n.d.	plant	pneumonia	Ringworms, liver disease, gonorrhea, syphilis, ear ache		[79]
164	Stilbaceae	*Nuxia floribunda* Benth.	n.d.	tree	bark	pneumonia; coughing		powdered, two tbsp., three times per day	[87]
165	Verbenaceae	*Lantana camara* L.	TMP: 87	shrub	leaves	cough	sore throat, conjunctivitis, toothache	the ash of burnt leaves together with a little salt	[72, 89]
			MJM 3213	shrub	root, leaves	cough	gonorrhea, syphilis, swollen legs, to dilate vagina during labour	the root/leaves boiled together with stem bark of *Mangifera indica* and *Ocimum basillicum* and decoction taken.	[63]

S. NO.	FAMILY	PLANT NAME	VOUCHER SPECIMEN	HABIT	PART USED	CLAIM FOR RESPIRATORY DISEASE RELATED CONDITION	OTHER CLAIMS FOR DISEASE CONDITION TREATMENT IN	PREPARATION	BOOK OR JOURNAL REFERENCE
			n.d.	n.d.	leaves	cough	sore throat, colds, conjunctivitis, toothache		[79]
166	Vitaceae	*Cyphostemma adenocaule* (Steud. Ex A. Rich.) Desc. Ex Wild & R.B.Drumm	TMP: 16, 154<br>TMRU: 443, 3127	herb	leaves	pneumonia		the leaves are put on the chest as a poultice	[72, 81, 89]
167	Xanthorrhoeaceae	*Aloe secundiflora* Engl.	n.d.	succulent plant	n.d.	pneumonia	antimalarial, chest pains, disinfection, conjunctivitis		[86]
			n.d.		leaves	TB			[65]
168		*Aloe lateritia* Engl.	TMRU: 2988	shrub	root	pneumonia	malaria, menorrhagia		[74, 77]
169	Zygophyllaceae	*Balanites aegyptiaca* (L.) Delile	n.d.	tree	bark	cough		powder in hot water plus salt; one cup three times a day; or lick powder with salt	[78]

Footnote:

HIV = Human immunodeficiency virus; TB = tuberculosis

Table S2: Anti-microbial activity of plants traditionally used against infective diseases of respiratory tract in Tanzania (see Table S1)
Plants with high activity (MIC ≤ 50 µg/mL, IZD ≥ 20 mm) are in marked bold.

SNo.	FAMILY	PLANT NAME	METHOD OF ANTI-MICROBIAL ACTIVITY TEST EMPLOYED	ANTIMICROBIAL ACTIVITY OF EXTRACT	COUNTRY	BOOK OR JOURNAL REFERENCE
	Acanthaceae	*Acanthus pubescens* (Oliv.) Engl.	Agar dilution streak method	**Leaf, stem, root** and **fruit** methanol extracts: no activity against BC, MS, PA, SG, and SA at concentrations of 1000 µg/mL.	Rwanda	[96]
			Microdilution method	**Root** dichloromethane extract: activity against SA, SF, BA, and SAG (MIC = 6.25 mg/mL each), and against BS (MIC = 3.12 mg/mL), no activity against ST and PA; root ethyl acetate extract: activity against SA and BA (MIC = 1.6 mg/mL each), ST (MIC = 3.12 mg/mL), PA (MIC = 6.25 mg/mL), and SAG (MIC = 25.0 mg/mL), no activity against SF and BS.	Tanzania	[64]
	Amaranthaceae	***Achyranthes aspera* L.**	Disc diffusion method	**Leaf** water extract: weak activity against PV at low dilutions. IZD =12 mm at 3000 ppm and IZD = 19 mm at 4000 ppm.	India	[97]
			Agar cup and microdilution methods (800 µg extract / well)	**Root** aqueous extract: activity against BL and STM (IZD = 10 mm each), and BB (IZD =12 mm), not active against PA, EC, SE, BS, and PF; MIC: no activity (MIC > 5000 µg/mL) for SE, BS, BB, PF, and STM. Methanol extact: activity against BL (IZD = 12 mm), and STM and BB (IZD = 14 mm), not active against PA, EC, SE, BS, and PF at concentrations of 20 mg/mL; MIC: activity against BB (MIC = 1000 µg/mL), and STM (MIC = 2000 µg/mL), no activity (MIC > 4000 µg/mL) for SE, BS, BB, and PF.	India	[98]
			Disc diffusion method	**Root** ethanol extract: at concentrations of 500 µg/mL high activity against STM, EC, and BS, moderate activity against PA and SAB; root acetone and ethyl acetate extracts: moderate activities against STM, EC, BS, and SA, no activity against SAB.	India	[99]

SNo.	FAMILY	PLANT NAME	METHOD OF ANTI-MICROBIAL ACTIVITY TEST EMPLOYED	ANTIMICROBIAL ACTIVITY OF EXTRACT	COUNTRY	BOOK OR JOURNAL REFERENCE
			Disc diffusion method (disc diameter: 5 mm)	**Herb** methanol extract (10 mg/mL): activity against SA and BS (IZD = 9 mm each), and ST (IZD = 7 mm); *n*-hexane extract: activity against SA, EC, and BS (IZD = 6 mm each).<br>**Leaf** chloroform extract (10 mg/mL): activity against SA and ST (IZD = 6 mm each).<br>**Infloroscence** chloroform extract (10 mg/mL): activity against SA and ST (IZD = 6 mm each); *n*-hexane extract: activity against SA, EC, and BS (IZD = 8 mm each).<br>**Stem** *n*-hexane extract (10 mg/mL): activity against SA, EC, and BS (IZD = 6 mm).	India	[100]
			Agar dilution streak method	**Leaf** and **stem** methanol extracts: no activity against BS, MS, PA, SG, and SA at concentrations of 1000 µg/mL	Rwanda	[96]
			Microdilution method	**Herb** methanol extract: no activity against MA (MIC > 500 µg/mL) and MS (MIC > 500 µg/mL).	n.d	[101]
			Microbial percent inhibition assay (50 µg extract / well)	**Leaf** methanol extract: activity against **EC (IZD = 20 mm)**, no activity against SA, hexane: activity against SA (IZD = 7 mm), no activity against EC.	Tonga	[102]
			Agar diffusion method	**Leaf and stem** ethanol (80%) extract: activity against BS and SA (MIC = 25 mg/mL), no activity against EC and PA.	India	[103]
			Agar well diffusion method (hole diameter: 10 mm)	**Root** water extract (50 mg/mL): activity against EC (IZD = 16 mm), no activity against SA and BS.	Somalia	[104]
			Agar well-diffussion method	**Flower** petroleum ether fraction: activity against SA and PA (IZD lower than standard antibiotics), PV and KP (IZD equal to the standard	Ethiopia	[105]

SNo.	FAMILY	PLANT NAME	METHOD OF ANTI-MICROBIAL ACTIVITY TEST EMPLOYED	ANTIMICROBIAL ACTIVITY OF EXTRACT	COUNTRY	BOOK OR JOURNAL REFERENCE
			(200 µg extract / well)	antibiotics), no activity against SG and EC; dichloromethane fraction: activity against PA (IZD lower than standard antibiotics), SA (IZD equal to the standard antibiotics), no activity against SG, EC, PV, and KP; methanol fraction: activity against KP (IZD lower than standard antibiotics), PV (IZD equal to standard antibiotics), no activity against SA, SG, EC, and PA; residual water fraction: activity against SA and EC (IZD lower than standard antibiotics), no activity against SG, PV, PA, and KP; direct water extract: activity against SA (IZD lower than standard antibiotics), no activity against SG, EC, PV, PA, and KP.		
3.		***Chenopodium ambrosioides* L.**	Agar dilution streak method	**Leaf, stem and fruit** methanol extracts: no activity against BS, MS, PA, SG and SA at concentrations of 1000 µg/mL	Rwanda	[96]
			Microplate alamar blue assay (MABA)	**Leaf** ethanol (80%) extract: activity against MS (MIC = 10 mg/mL), MTB (H37Ra) (MIC = 5 mg/mL), no activity against MTB (H37Rv) (MIC > 10 mg/mL).	Ghana	[106]
			Micro-titer plate dilution technique	**Leaf** dichloromethane:methanol (1:1) extract: activity against SA (MIC = 0.80 mg/mL), SA MR (MIC = 0.25 mg/mL), SA GMR (MIC = 0.50 mg/mL), SE (MIC = 0.50 mg/mL), and PA (MIC = 0.25 mg/mL); aqueous extract: activity against SA (MIC = 4.00 mg/mL), SA MR (MIC = 8.00 mg/mL), SA GMR (MIC = 8.00 mg/mL), SE (MIC = 16.00 mg/mL), no activity against PA (MIC > 16 mg/mL).	southern Africa	[32]
			Agar plate method	**Aerial parts** acetone extract: activity against MTB (H37Rv) (MIC = 0.5 mg/mL); water extract: no activity against MTB (H37Rv).	South Africa	[106]
			Microplate broth dilution method	**Aerial parts** *n*-hexane extract: activity against SA (MIC = 125 µg/mL), no activity against EF, KP, PA, and MS; dichloromethane extract: activity against SA, KP, and PA (MIC = 125 µg/mL each), no activity against EF and MS; ethylacetate extract: activity against SA (MIC = 250 µg/mL) and PA	Portugal	[31]

SNo.	FAMILY	PLANT NAME	METHOD OF ANTI-MICROBIAL ACTIVITY TEST EMPLOYED	ANTIMICROBIAL ACTIVITY OF EXTRACT	COUNTRY	BOOK OR JOURNAL REFERENCE
				(MIC = 125 μg/mL), no activity against EF, KP, and MS; methanol extract: activity against **SA (MIC = 31 μg/mL)**, no activity against EF, KP, PA, and MS.		
			Agar well-diffussion method (200 μg extract / well)	**Leaf** petroleum ether fraction: activity against SA, PV, and PA (IZD lower than standard antibiotics), no activity against SG, EC, and KP; dichloromethane, methanol, and residual water fractions: activity against SA (IZD lower than standard antibiotics), no activity against SG, EC, PA, PV, and KP; direct water extract: activity against SA (IZD greater than standard antibiotics), EC and PA (IZD lower than standard antibiotics), no activity against SG, PV, and KP.	Ethiopia	[105]
		Dysphania ambrosioides L. (syn. *Chenopodium ambrosioides* L.)	Microdilution method	**Leaf** essential oil: activity against SA (MIC = 256 μg/mL), and PA (MIC = 512 μg/mL).	Brazil	[107]
4.	Anacardiaceae	*Lannea schimperi* (Hoechst. ex A. Rich) Engl.	Microdilution method	**Stem bark** ethanol (80%) extract: activity against KP (MIC = 6.25 mg/mL) and ST (MIC = 1.6 mg/mL), no activity against EC and VC.	Tanzania	[108]
			Liquid dilution method	**Stem bark** methanol extract: activity against BC (MIC = 0.5 mg/mL), no activity against SA and KP; water extract: activity against BC (MIC = 1 mg/mL), no activity against SA.	Tanzania	[109]
5.		*Mangifera indica* L.	Disc diffusion method (800 μg extract / well)	**Leaf** water extract: activity against BB (IZD = 12 mm), no activity against PA, EC, SE, BL, BS, PF, and STM; methanol extract (20 mg/mL): activity against PF (IZD = 10 mm), BB and STM (IZD = 12 mm each), no activity against PA, EC, SE, BL, and BS.	India	[98]
			Agar disc diffusion assay (at 500 μg extract	**Leaf** ethanol extract: activity against CG (IZD = 17 ± 1 mm, MIC = 250 μL/disc).	Zimbabwe	[110]

SNo.	FAMILY	PLANT NAME	METHOD OF ANTI-MICROBIAL ACTIVITY TEST EMPLOYED	ANTIMICROBIAL ACTIVITY OF EXTRACT	COUNTRY	BOOK OR JOURNAL REFERENCE
			/disc) and microdilution method			
			BACTEC MGIT™ 960 system and disc diffusion method (15 mg extract/disc)	**Root** methanol extract: considerable activity against MTB H37Rv, activity against EC (IZD = 11.0 ± 0 mm, MIC = 234 μg), SA (IZD = 11.7 ± 0.3 mm, MIC = 117 μg), and CA (IZD = 12 ± 0.6 mm, MIC = 1875 μg).	Kenya	[111]
			REMA assay	**Root** *n*-hexane extract: no activity against MS and MU (MIC > 250 μg/mL each).<br>**Leaf** *n*-hexane extract: activity against MS (MIC = 250 μg/mL), no activity against MU (MIC > 250 μg/mL).	Ghana	[112]
			Agar diffusion method	**Stem bark** ethanol (80%) extract: activity against SA (MIC = 25 mg/mL), no activity against BS, EC, and PA.	India	[103]
6.		*Ozoroa insignis* Delile	Microdilution method	**Stem bark** ethanol (80%) extract: no activity against KP, ST, EC, and VC at concentrations < 25 mg/mL.	Tanzania	[108]
7.		***Ozoroa mucronata* (Bernh.) R. Fern & A. Fern.**	Microdilution method	**Leaf** acetone extract: activity against MB BCG (MIC = 0.625 ± 0 mg/mL), MB from field samples (MIC = 0.51 ± 0.17 mg/mL), MTB H37Ra (MIC = 0.625 ± 0 mg/mL), and MDR-TB (MIC = 0.51 ± 0.17 mg/mL).	South Africa	[113]
			n.d.	**Root bark** *n*-hexane extract: activity against SA and BS	Kenya	[114]
			Serial dilution assay with tetrazolium violet	**Leaf** crude (acetone (70%)-*n*-hexane) extract: activity against EC (MIC = 1250 μg/mL), EF (MIC = 625 μg/mL), PA (MIC = 312 μg/mL), and SA (MIC = 2500 μg/mL); *n*-hexane fraction: activity against EC (MIC = 78 μg/mL),	South Africa	[30]

SNo.	FAMILY	PLANT NAME	METHOD OF ANTI-MICROBIAL ACTIVITY TEST EMPLOYED	ANTIMICROBIAL ACTIVITY OF EXTRACT	COUNTRY	BOOK OR JOURNAL REFERENCE
				**EF (MIC = 39 μg/mL)**, PA (MIC = 156 μg/mL), and **SA (MIC = 19 μg/mL)**; dichloromethane fraction: activity against **EC and PA (MIC = 39 μg/mL each)**, **EF and SA (MIC = 19 μg/mL each)**; ethyl acetate fraction: activity against EC (MIC = 156 μg/mL), EF and SA (MIC = 625 μg/mL each), and PA (MIC = 312 μg/mL); butanol fraction: activity against EC (MIC = 312 μg/mL), EF and SA (MIC = 1250 μg/mL each), and PA (MIC = 2500 μg/mL).		
8.		*Pseudospondias microcarpa* (A. Rich.) Engl.	Disc diffusion method	**Leaf** petrol ether extract (2.25 mg/disc): activity against SA (IZD = 9.0 ± 0 mm), BS (IZD = 5.5 ± 0.7 mm), and EC (IZD = 7.5 ± 2.12 mm), no activity against PA; dichloromethane extract (4 mg/disc): activity against PA (IZD = 6.5 ± 0.7 mm), no activity against SA, BS, and EC; water extract (2.5 mg/disc): no activity against SA, BS, EC, and PA.	Tanzania	[115]
9.		*Rhus natalensis* Bernh. ex C. Krauss.	Disc diffusion method (disc diameter: 6 mm)	**Root** hot water extract (10 g/L): activity against EC (IZD = 9.7 ± 0.5 mm), SA (IZD = 12.0 ± 0.8 mm), and BS (IZD = 9.3 ± 1.2 mm).	Kenya	[116]
10.	Annonaceae	***Annona senegalensis* Pers.**	Cylinder plate technique (100 μg extract / well)	**Root bark** water extract: activity against SA (MIC = 1000 mg/mL, IZD = 18 mm), no activity against EC and PA; methanol extract: activity against SA (MIC = 62.5 mg/mL, IZD = 10 mm), no activity against PA and EC.	Uganda	[117]
			Disc diffusion method (0.5 mg extract /disc)	**Stem bark** extract: petrol fraction activity against **EC (IZD = 20-25 mm)**, and **SA (IZD > 25 mm)**; ether and chloroform fractions: no activity against SA and EC.	Tanzania	[118]
			REMA assay	**Stem** ethanol, **stem bark** dichloromethane, and **leaf** ethanol extracts: no activity against MS and MU (MIC > 250 μg/mL each).<br>**Twig** ethanol extract: no activity against MS and MU (MIC > 100 μg/mL each).	Cameroon	[112]

SNo.	FAMILY	PLANT NAME	METHOD OF ANTI-MICROBIAL ACTIVITY TEST EMPLOYED	ANTIMICROBIAL ACTIVITY OF EXTRACT	COUNTRY	BOOK OR JOURNAL REFERENCE
				**Stem** dichloromethane extract: activity against **MS (MIC = 31 μg/mL)** and MU (MIC = 250 μg/mL).		
			Disc diffusion method (2 mg extract / disc), microdilution	**Bark** ethanol extract: activity against AN (IZD = 4.5 mm, MIC = 1.6 mg/mL), AI (IZD = 5.0 mm, MIC = 3.1 mg/mL), SM (IZD = 3.0 mm, MIC = 12.5 mg/mL), and PG (IZD = 6.5 mm, MIC = 1.6 mg/mL), no activity against AA (IZD = 0.0 mm), and PI (IZD = 2.5 mm).	South Africa	[119]
11.		***Uvaria acuminata* Oliv.**	Disc diffusion method (0.5 mg extract /disc)	**Leaf** extract: ether fraction active against SA (IZD = 10 – 15 mm); petrol and chloroform fractions not active against SA.<br>**Root** extract: ether fraction active against **SA (IZD > 25 mm)**; petrol and chloroform fractions not active against SA.	Tanzania	[118]
12.	Apocynaceae	*Rauvolfia caffra* Sond.	Agar dilution method	**Root** aqueous and chloroform extracts: activity against EBC (MIC = 50 mg/mL).	South Africa	[120]
			Microdilution assay	**Leaf** *n*-hexane extract: activity against SA (MIC = 0.78 mg/mL), no activity against BS, EC, and KP; ethanol extract: activity against SA (MIC = 1.56 mg/mL), no activity against BS, EC, and KP; water extract: no activity against BS, EC, KP, and SA.	South Africa	[121]
			Two fold microdilution method	**Root** alkaloidal extract: activity against MMA and MIP (MIC = 1.25 mg/mL each); ethanol extract: activity against MMA and MIP (MIC = 2.5 mg/mL each).	Tanzania	[122]
			Micro-titer plate dilution technique	**Leaf** dichloromethane:methanol (1:1) extract: activity against SA (MIC = 2 mg/mL), SA MR (MIC = 4 mg/mL), SA GMR (MIC = 4 mg/mL), SE (MIC = 4 mg/mL), and PA (MIC = 2 mg/mL); aqueous extract: activity against SA (MIC = 8 mg/mL), SA MR (MIC = 8 mg/mL); SA GMR (MIC = 4 mg/mL), and SE (MIC = 16 mg/mL), no activity against PA (MIC > 16 mg/mL).	southern Africa	[32]

SNo.	FAMILY	PLANT NAME	METHOD OF ANTI-MICROBIAL ACTIVITY TEST EMPLOYED	ANTIMICROBIAL ACTIVITY OF EXTRACT	COUNTRY	BOOK OR JOURNAL REFERENCE
			Plate-hole diffusion method	**Bark** methanol and water extracts: no activity against SE and SA.	South Africa	[123]
13.	Asparagaceae	*Asparagus africanus* Lam.	Broth microdilution method	**Leaf** ethanol extract: activity against EF (MIC = 512 μg/mL), no activity against SA, EC, and PA.	Ethiopia	[124]
			Broth microdilution method	**Leaf** petroleum ether extract: activity against KP (MIC = 12.50 mg/mL), MA A+ (MIC = 6.25 mg/mL), and SA (MIC = 12.50 mg/mL); dichloromethane extract: activity against KP (MIC = 12.50 mg/mL), MA A+ (MIC = 6.25 mg/mL), and SA (MIC = 12.50 mg/mL); ethanol (80%) extract: activity against KP (MIC = 6.25 mg/mL), MA A+ (MIC = 0.39 mg/mL), and SA (MIC = 6.25 mg/mL).); water extract: activity against KP (MIC = 1.56 mg/mL), MA A+ (MIC = 6.25 mg/mL), and SA (MIC = 12.50 mg/mL)	South Africa	[125]
			Agar well-diffussion method (200 μg extract / well)	**Root** petroleum ether and dichloromethane fractions: no activity against SA, SG, EC, PV, PA, and KP; methanol fraction: activity against SA and PA (IZD lower than standard antibiotics), no activity against SG, EC, PV, and KP; residual water fraction: activity against SA, EC, and PA (IZD lower than standard antibiotics), no activity against SG, PV, and KP; direct water extract: activity against EC and PV (IZD lower than standard antibiotics), no activity against SA, SG, PA, and KP.	Ethiopia	[105]
14.		*Dracaena steudneri* Engl.	Agar dilution streak method	**Leaf** and **stem** methanol extracts: no activity against BS, MS, PA, SG and SA at concentrations of 1000 μg/mL.	Rwanda	[96]
15.	Bignoniaceae	***Kigelia africana* (Lam.) Benth.**	Liquid dilution method	**Stem bark** *n*-hexane extract: active against BC (MIC = 0.25 mg/mL) and SA (MIC = 0.5 mg/mL).	Tanzania	[109]

SNo.	FAMILY	PLANT NAME	METHOD OF ANTI-MICROBIAL ACTIVITY TEST EMPLOYED	ANTIMICROBIAL ACTIVITY OF EXTRACT	COUNTRY	BOOK OR JOURNAL REFERENCE
			In vitro broth microdilution screening assay	**Leaf** *n*-hexane extract: activity against MS (MIC = 0.63 mg/mL); dichloromethane extract: activity against MS (MIC = 1.67 mg/mL); acetone extract: activity against MS (MIC = 0.63 mg/mL); methanol extract: activity against MS (MIC = 0.63 mg/mL).	South Africa	[126]
			Microtiter plate assay	**Stembark** aqueous extract: activity against EC and SA (MLC = 2.5 mg/mL each); no activity against BS and KP.<br>**Stembark** ethanol extract: activity against BS (MLC = 0.63 mg/ml), EC, KP, and SA (MLC = 2.5 mg/mL each).<br>**Fruit** aqueous extract: no activity.<br>**Fruit** ethanol extract: activity against BS and KP (MLC = 1.25 mg/mL each).<br>**Fruit** ethyl acetate extract: activity against BS (MLC = 0.313 mg/mL).	South Africa	[127]
			Well-diffusion assay (well diameter: 4 mm)	**Bark** methanol extract (10 mg/mL): activity against SA (IZD = 3.00 ± 0.41 mm), *Streptococcus* group A (IZD = 5.00 ± 0.41 mm), EC (IZD = 1 ± 0 mmm), PA (IZD = 3.75 ± 0.50 mm).<br>**Fruit** methanol extract (10 mg/mL): activity against SA (IZD = 2.23 ± 0.50 mm), *Streptococcus* group A (IZD = 4.00 ± 0.41 mm), PA (IZD = 4.50 ± 0.58 mm), no activity against EC.<br>**Bark** methanol extract (10 mg/mL): activity against *Streptococcus* group A (IZD = 2.00 ± 0.41 mm), PA (IZD = 4.00 ± 0.82 mm), no activity against SA and EC.	Zimbabwe	[128]
			Microdilution method	**Bark** dichloromethane:methanol extract: activity against **SE (MIC = 10 μg/mL)**, BBA (MIC = 80 μg/mL), PA (MIC = 250 μg/mL), PBA (MIC = 310 μg/mL), SA GMR (MIC = 1000 μg/mL), BBL, SA MR, and EC (MIC = 2000 μg/mL each), no activity against SA (MIC > 8000 μg/mL); water extract: activity against SA GMR, SA MR, SE, and EC (MIC = 4000 μg/mL each), PBA (MIC = 6000 μg/mL), BBA and SA (MIC = 8000 μg/mL each), no activity against BBL and PA (MIC > 8000 μg/mL each).	South Africa	[129]

SNo.	FAMILY	PLANT NAME	METHOD OF ANTI-MICROBIAL ACTIVITY TEST EMPLOYED	ANTIMICROBIAL ACTIVITY OF EXTRACT	COUNTRY	BOOK OR JOURNAL REFERENCE
				**Fruit** dichloromethane:methanol extract: activity against BBA (MIC = 60 μg/mL), SE (MIC = 130 μg/mL), PA (MIC = 250 μg/mL), PBA (MIC = 310 μg/mL), BBL and SA GMR (MIC = 1000 μg/mL each), SA MR and EC (MIC = 2000 μg/mL each), no activity against SA; water extract: activity against PBA (190 μg/mL), BBA and SE (MIC = 250 μg/mL each), SA GMR and EC (MIC = 2000 μg/mL each), BBL (MIC = 8000 μg/mL), no activity against SA MR, SA, and PA (MIC > 8000 μg/mL each).		
			Micro-titer plate dilution technique	**Fruit** dichloromethane:methanol (1:1) extract: activity against SA, SA MR, and SA GMR (MIC = 4 mg/mL each), SE (MIC = 1.5 mg/mL), and PA (MIC = 2 mg/mL); water extract: activity against SA, and PA (MIC = 16 mg/mL each); no activity against SA MR, SA GMR, and SE (MIC > 16 mg/mL each).	southern Africa	[32]
			Inhibition zone (MIC)	**Leaf** methanol extract: activity against SA (5 mg/mL), BS (MIC = 2.5 mg/mL), EC (MIC = 5.5 mg/mL), and PA (MIC = 7.5 mg/mL).<br>**Stem bark** methanol extract activity against SA (5 mg/mL), BS (MIC = 5.5 mg/mL), EC (MIC = 5.25 mg/mL), and PA (MIC = 7.5 mg/mL).	Ghana	[130]
			Disc diffusion method (20 mg/disc), microdilution method	**Stem bark** methanol extract: activity against EC and PA (IZD = 6.0 ± 0.0 mm each, MIC = 0.83 ± 0.0 μg/mL each), SA and ST (IZD = 0.0 ± 0.0 mm each, MIC = 0.83 ± 0.0 μg/mL each).	Cameroon	[131]
			Microdilution method	**Fruit** methanol extract: activity against ABB (MIC = 1874 μg/mL), AF (MIC = 1247 μg/mL), AH (MIC = 141.7 μg/mL), CF (MIC = 1814 μg/mL), EA (MIC = 236 μg/mL), EC (MIC = 176.3 μg/mL), PM (MIC = 181.4 μg/mL), PF (MIC = 187.4 μg/mL), SRM (MIC = 1572 μg/mL), BC (MIC = 1189 μg/mL), SA (MIC = 2487 μg/mL), SE (MIC = 445.4 μg/mL).<br>**Fruit** water extract: activity against AF (MIC = 1786 μg/mL), AH (MIC = 1288 μg/mL), CF (MIC = 2060 μg/mL), EA (MIC = 173.7μg/mL), EC (MIC = 84.7 μg/mL), , PM (MIC = 206.2 μg/mL), PF (MIC = 206.3 μg/mL), SRM (MIC =	Australia	[132]

SNo.	FAMILY	PLANT NAME	METHOD OF ANTI-MICROBIAL ACTIVITY TEST EMPLOYED	ANTIMICROBIAL ACTIVITY OF EXTRACT	COUNTRY	BOOK OR JOURNAL REFERENCE
				2118 μg/mL), BC (MIC = 1974 μg/mL), SA (MIC = 4333 μg/mL), SE (MIC = 847.9 μg/mL), no activity against ABB.<br>**Fruit** ethyl acetate extract: active against EA (MIC = 423.6 μg/mL), PM (MIC = 623.3 μg/mL), PF (MIC = 687.3 μg/mL), SE (MIC = 1655 μg/mL), not active against ABB, AF, AH, CF, EC, SRM, BC, and SA.		
			Micro-titer plate method	**Fruit** acetone extract: activity against KP (MIC = 4000 μg/mL), MC (MIC = 1000 μg/mL), MS (MIC = 4000 μg/mL), and MA (MIC = 250 μg/mL); no activity against SA (MIC > 8000 μg/mL).	Central Africa	[133]
			Serial microplate dilution method	**Leaf** *n*-hexane extract: activity against SA (MIC = 2.50 mg/mL), EC (MIC = 1.25 mg/mL), PA (MIC = 0.60 mg/mL), and EF (MIC = 0.30 mg/mL); dichloromethane extract: activity against SA and EC (MIC = 0.60 mg/mL each), PA (MIC = 1.25 mg/mL), and EF (MIC = 0.08 mg/mL); acetone extract: activity against SA and EC (MIC = 0.60 mg/mL each), PA (MIC = 0.10 mg/mL), and EF (MIC = 0.42 mg/mL).	South Africa	[134]
			Broth dilution method	**Leaf** dichloromethane extract: activity against MA (MIC = 6.25 mg/mL); ethylacetate extract: no activity against MA (MIC = 12.5 mg/mL); ethanol extract activity against MA (MIC = 6.25 mg/mL).<br>**Bark** dichloromethane extract: activity against MA (MIC = 3.12 mg/mL); ethylacetate extract: no activity against MA (MIC = 12.5 mg/mL); ethanol extract activity against MA (MIC = 1.56 mg/mL).	Sudan	[135]
			Broth microdilution method	**Aerial parts** methanol (70%) extract: activity against PM, SRM, and KP (MIC = 1.80 mg/mL each), no activity against EC, SHF, SPT, SEN, and SA (MIC > 2.50 mg/mL each).	Togo	[136]
16.		*Stereospermum kunthianum* Cham.	Minimum inhibitory	**Chewing stick** methanol/dichloromethane (1:1) extract: activity against BC (MIC = 1.5 mg/mL), SA (MIC = 0.4 mg/mL), EF (MIC = 4.0 mg/mL), SM (MIC = 16 mg/mL), KP (MIC = 4.0 mg/mL), and EC (MIC = 1.0 mg/mL);	Ethiopia	[137]

SNo.	FAMILY	PLANT NAME	METHOD OF ANTI-MICROBIAL ACTIVITY TEST EMPLOYED	ANTIMICROBIAL ACTIVITY OF EXTRACT	COUNTRY	BOOK OR JOURNAL REFERENCE
			concentration assay	water extract: activity against BC (MIC = 0.4 mg/mL), SA (MIC = 1.0 mg/mL), EF and KP (MIC = 4.0 mg/mL each), SM (MIC = 2.0 mg/mL); no activity against EC.		
			Kirby-Bauer's disc diffusion method	**Leaf** methanol extract: activity against EF and SA (MIC = 0.67 mg/mL each), AB (MIC = 3.41 mg/mL), CF, EA, EC, and PM (MIC = 4.27 mg/mL each), KP and PA (MIC = 9.63 mg/mL each).	India	[138]
17.	Borangiaceae	*Cordia africana* Lam.	Agar dilution method	**Stem bark and leaf** distilled water extract: activity against NG, SP, and SPN, no activity against BC, SA, ST, STM, SHF, SD, and EC.	Ethiopia	[139]
			Agar well diffusion	**Leaf** methanol (80%) extract: no activity against EC and SA.	Ethiopia	[140]
			Agar disc diffusion assay (diameter: 6 mm)	**Leaf** ethanol extract (100 mg/mL): activity against SA (IZD = 7.3 ± 1.15 mm), STM (IZD = 9.1 ± 1.04 mm), PA (IZD = 5 ± 1.0 mm), SHS (IZD = 8 ± 0.0 mm), and EC (IZD = 11.6 ± 0.57 mm); methanol extract (100 mg/mL: activity against SA (IZD = 5.8 ± 0.76 mm), STM (IZD = 8.5 ± 0.5 mm), PA (IZD = 4 ± 1.0 mm), SHS (IZD = 6.8 ± 0.76 mm), and EC (IZD = 12.8 ± 1.25 mm); chloroform extract (100 mg/mL: no activity against SA, STM, PA, SHS, and EC.	Ethiopia	[141]
18.		***Tecomaria capensiss* (Thunb.) Spach.**	Microdilution assay	**Bark** *n*-hexane extract: no activity against BS, EC, KP, and SA; ethanol extract: activity against SA (MIC = 3.13 mg/mL), no activity against BS, EC, and KP; water extract: no activity against BS, EC, KP, and SA.	South Africa	[121]
			Disc diffusion method (0.5 mg extract/disc)	**Leaf** ether extract: activity against **SA (IZD = 15-20 mm)** and **EC (IZD = 15-20 mm)**; chloroform extract: no activity against SA and EC.	Tanzania	[118]
			Microplate broth dilution method	**Aerial parts** *n*-hexane extract: activity against PA and MS (MIC = 125 μg/mL each), and no activity against SA, EF, and KP; methanol extract:	Portugal	[31]

SNo.	FAMILY	PLANT NAME	METHOD OF ANTI-MICROBIAL ACTIVITY TEST EMPLOYED	ANTIMICROBIAL ACTIVITY OF EXTRACT	COUNTRY	BOOK OR JOURNAL REFERENCE
				activity against SA (MIC = 250 µg/mL), no activity against EF, KP, PA, and MS.		
			Agar plate diffusion method	**Leaf** methanol extract (2 mg/mL): activity against EC and BC (IZD = 16±1.63 mm each), SA (IZD = 14±0.82 mm), PA (IZD = 12±0.82 mm), and EF (IZD = 15±0.41 mm).	India	[142]
			Micro-titre plate dilution assay	**Leaf** dichloromethane:methanol extract: activity against SM (MIC = 0.67 mg/mL), SS (MIC = 2 mg/mL), LA (MIC = 2.67 mg/mL), LC (MIC = 3.33 mg/mL), PG (MIC = 1 mg/mL), and FN (MIC = 1.3 mg/mL); water extract: activity against SM (MIC = 2 mg/mL), SS, PG, and FN (MIC = 8 mg/mL each), no activity against LA and LC.<br>**Stem** dichloromethane:methanol extract: activity against SM (MIC = 2 mg/mL), SS (MIC = 4 mg/mL), PG (MIC = 1 mg/mL), and FN (MIC = 2.67 mg/mL), no activity against LA and LC; water extract: activity against SM (MIC = 2 mg/mL) and FN (MIC = 6 mg/mL), no activity against SS, LA, LC, and PG.	South Africa	[143]
19.		*Trichodesma zeylanicum* (Burm.f.) R.Br.	Broth microdilution technique	**Leaf** methanol (80%) extract: activity against SA, PA, and ST (MIC = 1.25 mg/mL), no activity against SP, BA, BC, KP, and EC.	Tanzania	[144]
20.	Canellaceae	***Warburgia salutaris* (Bertol.f.) Chiov.**	Broth microdilution method	**Leaf** acetone extract: activity against MA (MIC = 0.625 mg/mL), MTB (TB8104) (MIC = 0.156 mg/mL), MS and MF (MIC = 1.25 mg/mL each).	South Africa	[145]
			Resazurin microplate assay (REMA)	**Leaf** acetone extract: activity against **MTB (H37Ra) (MIC = 25±2 µg/mL)** and **MTB 2 (MIC = 25 ± 5 µg/mL)**.	South Africa	[26]

SNo.	FAMILY	PLANT NAME	METHOD OF ANTI-MICROBIAL ACTIVITY TEST EMPLOYED	ANTIMICROBIAL ACTIVITY OF EXTRACT	COUNTRY	BOOK OR JOURNAL REFERENCE
			Microdilution method	**Bark** methanol extract: activity against KP (MIC = 1.00 mg/mL), MC (MIC = 2.00 mg/mL), PA and SA (MIC = 1.66 mg/mL each).<br>**Bark** dichloromethane extract: activity against KP (MIC = 1.00 mg/mL), MC (MIC = 0.42 mg/mL), PA (MIC = 0.25 mg/mL), and SA (MIC = 0.50 mg/mL).	South Africa	[146]
			Well-diffusion assay (well diameter: 4 mm)	**Bark** methanol extract (10 mg/mL): activity against SA (IZD = 5.00 ± 0.82 mm), *Streptococcus* group A (IZD = 3.00 ± 0.41 mm), no activity against EC and PA.<br>**Leaf** methanol extract (10 mg/mL): activity against SA (IZD = 2 ± 0 mm), PA (IZD = 2 ± 0.82 mm), no activity against *Streptococcus* group A and EC.<br>**Root** methanol extract (10 mg/mL): activity against SA (IZD = 5.50 ± 0.41 mm), *Streptococcus* group A (IZD = 9.50 ± 0.58 mm), EC (IZD = 3.5 ± 0.58 mm), and PA (IZD = 2 ± 0.41 mm).	Zimbabwe	[128]
			Micro-titer plate dilution technique	**Bark** dichloromethane:methanol (1:1) extract: activity against SA (MIC = 0.40 mg/mL), SA MR, SE, and SA GMR (MIC = 0.50 mg/mL each), and PA (MIC = 0.10 mg/mL); aqueous extract: activity against SA, SA MR, SA GMR (MIC = 4.0 mg/mL, each), and SE (MIC = 8.0 mg/mL), no activity against PA (MIC > 16 mg/mL).<br>**Leaf** dichloromethane:methanol (1:1) extract: activity against SA (MIC = 0.8 mg/mL), SA MR (MIC = 2.0 mg/mL), SA GMR (MIC = 0.50 mg/mL), SE and PA (MIC = 1.0 mg/mL each); aqueous extract: activity against SA and SA MR (MIC = 4.0 mg/mL), SA GMR (MIC = 8.0 mg/mL), and SE (MIC = 16.0 mg/mL), no activity against PA (MIC > 16 mg/mL).	southern Africa	[32]
			Micro-titre plate dilution assay	**Leaf** dichloromethane:methanol extract: activity against SM and LA (MIC = 1 mg/mL each), SS (MIC = 1.67 mg/mL), LC (MIC = 2 mg/mL), PG (MIC = 8 mg/mL), and FN (MIC = 3.33 mg/mL); aqueous extract: active against SM and PG (MIC = 8 mg/mL each), FN (MIC = 4 mg/mL), no activity against SS, LA and LC.	South Africa	[143]

SNo.	FAMILY	PLANT NAME	METHOD OF ANTI-MICROBIAL ACTIVITY TEST EMPLOYED	ANTIMICROBIAL ACTIVITY OF EXTRACT	COUNTRY	BOOK OR JOURNAL REFERENCE
				**Bark** dichloromethane:methanol extract: activity against SM and SS (MIC = 2 mg/mL each), LA (MIC = 1.33 mg/mL), LC (MIC = 0.42 mg/mL), PG (MIC = 0.83 mg/mL), and FN (MIC = 4 mg/mL); aqueous extract: activity against SM (MIC = 6 mg/mL), SS and LC (MIC = 8 mg/mL each), PG (MIC = 2 mg/mL), and FN (MIC = 1 mg/mL), no activity against LA.<br>**Twigs** dichloromethane:methanol extract: activity against SS, LA and PG (MIC = 1.33 mg/mL each), LS (MIC = 1 mg/mL) and FN (MIC = 2 mg/mL), no activity against SM; aqueous extract: activity against SS, LA, and FN (MIC = 8 mg/mL), and PG (MIC = 4 mg/mL), no activity against SM and LC.		
			Broth microdilution method	**Stem bark** ethanol (80%) extract: activity against EF (MIC = 512 µg/mL), and SA (MIC = 256 µg/mL), no activity against EC and PA.	Uganda	[147]
			Plate-hole diffusion method	**Bark** methanol and water extracts: no activity against SE and SA.	South Africa	[148]
21.		*Warburgia ugandensis* Sprague	Disc-diffusion method (5 mg extract / disc)	**Leaf** freeze-dried methanol extract: activity against EC and SA (IZD = 9 mm), VS (IZD = 10 mm), and BC (IZD = 8 mm), air-dried methanol extract: EC, SA and VS (IZD = 10 mm), BC (IZD = 8 mm), both extracts no activity against ST, PA, PM, and SHF.	Tanzania	[149]
			Paper disc assay, agar assay	**Stem** water extract: no activity against SA and EC in paper disc assay, activity against SA and EC in agar assay; petroleum ether and ethanol extracts: no activity against SA and EC in both assays.	Uganda	[150]
			Agar dilution method	**Leaf** methanol extract (2000 µg/mL): activity against BC, NG, and SA no activity against SP, SPN ST, SHF, STM, SD, and EC.	Ethiopia	[139]

SNo.	FAMILY	PLANT NAME	METHOD OF ANTI-MICROBIAL ACTIVITY TEST EMPLOYED	ANTIMICROBIAL ACTIVITY OF EXTRACT	COUNTRY	BOOK OR JOURNAL REFERENCE
				**Stem bark** methanol extract (2000 µg/mL): activity against BC, NG, and SA no activity against SP, SPN ST, SHF, STM, SD, and EC.		
22.	Cannabaceae	*Trema orientalis* (L.) Blume	Minimum inhibitory concentration	**Bark** aqueous extract: activity against KP and BS (MIC = 0.625 mg/mL each), PA and PV (MIC = 1.25 mg/mL each), SA and EC (MIC = 2.5 mg/mL each).	India	[151]
			Broth microdilution method	**Leaf** acetone extract: activity against MS and MTB (MIC = 0.312 mg/mL each), MF (MIC = 0.625 mg/mL), and MA (MIC = 1.25 mg/mL).	South Africa	[145]
23.	Capparaceae	*Boscia angustifolia* A. Rich	Liquid dilution method	**Stem bark** *n*-hexane extract: activity against BC (MIC = 0.5 mg/mL) and SA (MIC = 1 mg/mL).	Tanzania	[109]
			Microtitre dilution technique	**Bark** methanol (80%) extract: activity against SA, BS, ST, EC, and PA (MIC = 3.75 mg/50 µL each).	Kenya	[152]
			Antimycobacterial susceptibility test (BACTEC MGIT™ 960 system)	**Bark** methanol extract: activity against MK (501 GUs at 0.5 mg/mL extract, 0 GUs at 1 mg/mL), MTB (183 GUs at 0.5 mg/mL, 0 GUs at 1 mg/mL), MF (618 GUs at 0.5 mg/mL, 0 GUs at 1 mg/mL), slight activity against MS (13,801 GUs at 0.5 mg/mL, 120 GUs at 1 mg/mL, 9 GUs at 2 mg/mL).	Kenya	[153]
24.		*Cadaba farinosa* Forssk.	Agar diffusion technique	**Leaf** dichloromethane extract: activity against SA and BC (MIC = 500 µg/mL each), and MCF (MIC = 250 µg/mL), no activity against EC, and PA; methanol extract: activity against BC (MIC = 250 µg/mL), and MCF (MIC = 1000 µg/mL), no activity against SA, EC, and PA; water extract: no activity against SA, BC, MCF, EC, and PA.	Yemen	[36]

SNo.	FAMILY	PLANT NAME	METHOD OF ANTI-MICROBIAL ACTIVITY TEST EMPLOYED	ANTIMICROBIAL ACTIVITY OF EXTRACT	COUNTRY	BOOK OR JOURNAL REFERENCE
25.		***Capparis erythrocarpos* Isert**	Agar well diffusion assay (well diameter: 10 mm) + serial dilution methods	**Leaf** petroleum ether (250 mg/mL): activity against **EC (IZD = 25.3±1.53 mm**, MIC = 320 µg/mL), **SA (IZD 26.3±0.58 mm**, MIC = 400 µg/mL), and PA (IZD = 13.0±0.00 mm, MIC = 500 µg/mL); methanol extract (250 mg/mL): activity against SA (IZD = 10.3±0.58 mm, MIC = 530 µg/mL), no activity against EC and PA (IZD = 0.0±0.00 mm each, MIC > 1600 µg/mL each).	Uganda	[154]
26.		***Capparis tomentosa* Lam.**	Disc diffusion method (3 mg extract / disc) + microdilution method	**Bark** acetone extract: activity against SE MR (IZD = 19 mm, MIC = 0.312 mg/mL), **SA MR (IZD = 23 mm**, MIC = 0.312 mg/mL), EC (IZD = 5 mm, MIC = 0.625 mg/mL), and EBC (IZD = 7 mm, MIC = 0.625 mg/mL); *n*-hexane extract: activity against **SE MR (IZD = 22 mm**, MIC = 0.156 mg/mL), **SA MR (IZD = 25 mm**, MIC = 0.156 mg/mL), EC (IZD = 8 mm, MIC = 0.625 mg/mL), and EBC (IZD = 6 mm, MIC = 0.625 mg/mL); methanol extract: activity against **SE MR (IZD = 26 mm**, MIC = 0.078 mg/mL), **SA MR (IZD = 30 mm**, MIC = 0.078 mg/mL), not active against EC and EBC; water extract: no activity against SE MR, SA MR, EC, and EBC.	South Africa	[155]
			Broth microdilution method	**Root** ethanol (70%) extract: activity against MTB H37Rv (MIC = 125 µg/mL), no activity against MS ATCC 607, MTB H37Ra, MS mc^2, MB BCG, MBA DSM 44156, and MBA DSM 44157.	Mozambique	[156]
			Plate-hole diffusion method	**Root** methanol and water extracts: no activity against SE and SA.	South Africa	[148]
27.	Celastraceae	***Elaeodendron buchananii* (Loes.) Loes.**	Broth microdilution method	**Stem bark** *n*-hexane extract: no activity against SA (MIC = 250 µg/mL), and SP, SAL, EC, VC, SD, and NM; ethyl acetate extract: activity against **SA and NM (MIC = 15.62 µg/mL each)**, SP and EC (MIC = 62.5 µg/mL each), **SAL (MIC = 31.25 µg/mL)**, no activity against VC (MIC = 125 µg/mL), and SD (MIC = 250 µg/mL); methanol extract: no activity against SA, SAL, and EC (MIC = 125 µg/mL each), SP, VC, and SD (MIC = 250 µg/mL each), and NM (MIC > 500 µg/mL).	Kenya	[34]

SNo.	FAMILY	PLANT NAME	METHOD OF ANTI-MICROBIAL ACTIVITY TEST EMPLOYED	ANTIMICROBIAL ACTIVITY OF EXTRACT	COUNTRY	BOOK OR JOURNAL REFERENCE
				According to the authors, values of > 62.5 μg/mL were considered not active.		
28.		*Maytenus senegalensis* (Lam.) Excell.	Microdilution method	**Stem bark** ethanol extract: activity against KP and EC (MIC = 12.5 mg/mL each), ST (MIC = 6.25 mg/mL), and VC (MIC = 0.8 mg/mL).	Tanzania	[108]
			In vitro broth microdilution screening assay	**Leaf** *n*-hexane extract: activity against MS (MIC = 0.104 mg/mL); dichloromethane extract: no activity against MS; acetone extract: activity against MS (MIC = 0.523 mg/mL); methanol extract: activity against MS (MIC = 1.25 mg/mL).	South Africa	[126]
			Agar plate method	**Aerial parts** acetone extract: activity against MTB (H37Rv) (MIC = 0.5 mg/mL), water extract: no activity against MTB.<br>**Root** acetone and water extracts: no activity against MTB (H37Rv).	South Africa	[106]
29		***Gymnosporia senegalensis* (Lam.) Loes** (syn. *Maytenus senegalensis* (Lam.) Excell.)	Microdilution assay	**Root** petroleum ether extract: activity against **BS (MIC = 0.39 μg/mL)**, **EC and KP (MIC = 1.56 μg/mL each)**, and **SA (MIC = 6.25 μg/mL)**; dichloromethane extract: activity against **BS and SA (MIC = 0.78 μg/mL** each), **EC (MIC = 3.13 μg/mL),** and **KP (MIC = 1.56 μg/mL)**; ethanol extract: activity against **EC (MIC = 0.10 μg/mL)**, **BS and KP (MIC = 1.56 μg/mL** each), and **SA (MIC = 0.78 μg/mL each)**; water extract: activity against **BS, EC, KP, and SA (MIC = 3.13 μg/mL each)**.	South Africa	[24]
			Well-diffusion assay (well diameter: 4 mm)	**Leaf** methanol extract (10 mg/mL): activity against SA (IZD = 2.50 ± 0.41 mm), *Streptococcus* group A (IZD = 3.00 ± 0.71 mm), EC (IZD = 4.50 ± 0.58 mm), and PA (IZD = 5.00 ± 0.82 mm).<br>**Root** methanol extract (10 mg/mL): activity against SA (IZD = 5.13 ± 0.63 mm), *Streptococcus* group A (IZD = 2 ± 0 mm), EC (IZD = 4.00 ± 0.41 mm), and PA (IZD = 1 ± 0 mm).	Zimbabwe	[128]

SNo.	FAMILY	PLANT NAME	METHOD OF ANTI-MICROBIAL ACTIVITY TEST EMPLOYED	ANTIMICROBIAL ACTIVITY OF EXTRACT	COUNTRY	BOOK OR JOURNAL REFERENCE
				**Twig** methanol extract (10 mg/mL): activity against SA (IZD = 4.00 ± 0.41 mm), *Streptococcus* group A and EC (IZD = 2 ± 0 mm each), and PA (IZD = 7.00 ± 0.41 mm).		
30.	Chrysobalanaceae	***Parinari curatellifolia* Planch. ex Benth.**	Zone of inhibition (20 mg extract / hole), MIC	**Stem** aqueous extract (100 mg/mL): activity against SA (IZD = 8.20 ± 0.06 mm, MIC = 3.125 mg/mL), SAU (IZD = 6.53 ± 0.03 mm, MIC = 6.25 mg/mL), SP (IZD = 6.73 ± 0.07 mm, MIC = 12.5 mg/mL), SM (IZD = 8.00 ± 0.06 mm, MIC = 6.25 mg/mL), and BS (IZD = 9.10 ± 0.06 mm, MIC = 25.00 mg/mL); ethanol extract (100 mg/mL): activity against SA (IZD = 17.63 ± 0.09 mm, MIC = 6.25 mg/mL), SAU (IZD = 16.2 ± 0.09 mm, MIC = 12.50 mg/mL), SP (IZD = 14.90 ± 0.06 mm, MIC = 25 mg/mL), SM (IZD = 18.20 ± 0.06 mm, MIC = 25 mg/mL), and BS (IZD = 11.30 ± 0.06 mm, MIC = 25.00 mg/mL).	Nigeria	[157]
			Broth microdilution method	**Aerial parts** methanol (70%) extract: activity against PM, SRM, SHF, and SPT (MIC = 1.80 mg/mL each), no activity against EC, SEN, KP, and SA (MIC > 2.50 mg/mL each).	Togo	[136]
			Agar diffusion method	**Stem** water extract (2.8 g/100 mL): activity against **PA and BAU (IZD = 25 mm** each), **ST (IZD = 27 mm)**, ***Klebsiella* spp. (IZD = 28 mm)**, **EC (IZD = 20 mm)**, **BS (IZD = 29 mm)**, and **SA (IZD = 36 mm)**; methanol extract (2.8 g/100 mL): activity against PA (IZD = 16 mm), ST and EC (IZD = 14 mm each), *Klebsiella* spp. (IZD = 13 mm), BAU (IZD = 19 mm), **BS (IZD = 21 mm)**, and **SA (IZD = 20 mm)**; *n*-hexane and diethylether extracts (2.8 g/100 mL each) showed no activity against PA, SP, *Klebsiella* spp, BAU, EC, BS, and SA.	Nigeria	[158]
			Disc diffusion method (2 mg extract / disc), microdilution	**Bark** ethanol extract: active against AN (IZD = 4.6 mm, MIC = 1.6 mg/mL), AI (IZD = 2.5 mm, MIC = 1.6 mg/mL), SM (IZD = 3.6 mm, MIC = 6.3 mg/mL), AA (IZD = 0.0 mm, MIC = 12.5 mg/mL), and PG (IZD = 2.4 mm, MIC = 3.1 mg/mL), not active against and PI (IZD = 3.0 mm).	South Africa	[159]

SNo.	FAMILY	PLANT NAME	METHOD OF ANTI-MICROBIAL ACTIVITY TEST EMPLOYED	ANTIMICROBIAL ACTIVITY OF EXTRACT	COUNTRY	BOOK OR JOURNAL REFERENCE
			Agar disc diffusion assay (500 μg extract / disc)	**Stem** ethanol extract: activity against MA (IZD = 18 ± 1 mm at 500 μg/disc, MIC = 8 μL/disc) and CG (IZD = 13 ± 1 mm at 500 μg/disc, MIC = 125 μL/disc).<br>**Leaf** ethanol extract: activity against MA (IZD = 18 ± 1 mm at 500 μg/disc, MIC = 63 μL/disc).	Zimbabwe	[110]
			Plate-hole diffusion method	**Bark** methanol and water extracts: no activity against SE and SA.	South Africa	[148]
31.	Combretaceae	*Combretum collinum* Fresen.	Microplate alamar blue assay (MABA)	**Leaf** ethanol extract: no activity against MS (MIC > 1 mg/mL) and MTB (H37Rv) (MIC > 1 mg/mL), weak activity against PBA (MIC = 0.5 mg/mL).	South Africa	[160]
32.		***Combretum fragrans* F. Hoffm.**	Agar diffusion method (200 μL)	**Root** methanol extract (50 mg/mL): activity against ***Sarcina* sp. (IZD = 29 mm,** MIC = 7.3 mg/mL), **SA (IZD = 26 mg/mL)**, **EA (IZD = 30 mm)**, **SE (IZD = 22 mm)**, **BS (IZD = 23 mm)**, and **ML (IZD = 38 mm)**, no activity against EC (IZD = 0 mm).<br>**Leaf** methanol extract (50 mg/mL): activity against **SA and BS (IZD = 22 mg/mL each)**, **EA (IZD = 20 mm)**, SE (IZD = 18 mm), **ML (IZD = 34 mm)**, and ***Sarcina* sp. (IZD = 21 mm)**, no activity against EC (IZD = 0 mm).	Tanzania	[161]
33.		***Combretum zeyheri* Sond.**	Agar diffusion method (10 mg extract / test)	**Fruit** methanol extract: activity against SA, EA, SE, and BS (IZD = 18 mm each), and *Sarcina* sp. (IZD = 15 mm), no activity against EC and ML (IZD = 0 mm each).<br>**Root** methanol extract: activity against **SA (IZD = 22 mm)**, **EA (IZD = 23 mm)**, SE (IZD = 16 mm), BS (IZD = 19 mm), **ML (IZD = 28 mm)**, and ***Sarcina* sp. (IZD = 20 mm)**, no activity against EC (IZD = 0 mm).	Tanzania	[161]

SNo.	FAMILY	PLANT NAME	METHOD OF ANTI-MICROBIAL ACTIVITY TEST EMPLOYED	ANTIMICROBIAL ACTIVITY OF EXTRACT	COUNTRY	BOOK OR JOURNAL REFERENCE
				**Stem bark** methanol extract: activity against **SE (IZD = 20 mm)**, BS (IZD = 18 mm), **ML (IZD = 33 mm)**, and ***Sarcina* sp. (IZD = 24 mm)**, no activity against EC (IZD = 0 mm).		
			Broth dilution method	**Leaf** alkaloid extract: activity against MS (MIC = 125 μg/mL).	Zimbabwe	[162]
			Microplate method	**Leaf** methanol extract: activity against EC and BS (MIC = 10 ± 0.5 mg/mL each); water extract: activity against EC (MIC = 18.8 ± 0.7 mg/mL), and BS (MIC = 37 ± 0.5 mg/mL).	Zimbabwe	[163]
34.		*Terminalia kilimandscharica* Engl.	Broth dilution method	**Bark** methanol (70%) extract: activity against ML (MIC = 100 mg/mL), BC (MIC = 15.6 mg/mL), PA (MIC = 75 mg/mL), and EC (MIC = 150 mg/mL).	Kenya	[164]
35.	Compositae (Asteraceae)	*Ageratum conyzoides* (L.) L.	Tetrazolium microplate assay	**Whole plant** methanol (80%) extract: weak activity against MTB (H37Rv) (MIC = 1.6 mg/mL).	Malaysia	[165]
			REMA assay	**Leaf** *n*-hexane extract: no activity against MS and MU (MIC > 250 μg/mL each).	Ghana	[112]
			Well diffusion method	**Leaf** methanolic extract: no activity against SA BS6a, SA BS11, SA BS12, SA BS14, EC BS3, EC VT7b PA SH2b, *Proteus* spp. JH1a, JH5a, and SH9a, and *Shigella* spp. BS16.	Nigeria	[166]
			Agar well diffusion method	**Leaf** methanol (95%) extract: no activity against SA, SE, BC, EC, PA, and KP.	India	[167]
			Disc diffusion method (100 μg/disc)	**Leaf** methanol/chloroform extract: activity against SA (IZD = 19.5 ± 2.4 mm), EC (IZD = 8.1 ± 0.5 mm), no activity against BS, ST, EA, and BBS; water extract: no activity against SA, EC, BS, ST, EA, and BBS.	Pakistan	[168]

SNo.	FAMILY	PLANT NAME	METHOD OF ANTI-MICROBIAL ACTIVITY TEST EMPLOYED	ANTIMICROBIAL ACTIVITY OF EXTRACT	COUNTRY	BOOK OR JOURNAL REFERENCE
			Agar dilution method	**Whole plant** ethanol extract (2000 µg/mL): activity against SP and NG, no activity against BC, SPN, ST, SHF, STM, SA, SD, and EC.	Ethiopia	[139]
36.		***Solanecio mannii* (Hook.f.) C. Jeffre**y (syn. *Crassocephalum mannii* (Hook.f.) Milne-Redh.)	Disc diffusion method (50 µg/disc), microbroth dilution method	**Leaf** cyclohexane extract: activity against **SA** (IZD = 5 mm, **MIC = 6.3 µg/mL**), **SF and BC** (IZD = 2 mm each, **MIC = 12.5 µg/mL each)**, PA (IZD = 1 mm, MIC = 100 µg/mL), **SD** (IZD = 3 mm, **MIC = 25 µg/mL**), **SHF** (IZD = 2 mm, **MIC = 50 µg/mL**), no activity against BS and EC (IZD = 0 mm each), and KP (IZD = 1 mm).	Cameroon	[35]
37		*Crassocephalum mannii* (Hook.f.) Milne-Redh. (syn. *Solanecio mannii* (Hook.f.) C. Jeffrey)	Two fold microdilution method	**Stem bark** ethanol extract: activity against MMA (MIC = 0.625 mg/mL), and MIP (MIC = 1.25 mg/mL).	Tanzania	[169]
38.		*Microglossa pyrifolia* (Lam.) Kunze	Agar well diffusion method (500 µg extract / well), microdilution assay	**Root bark** ethanol extract: activity against BS (IZD = 0 mm, MIC = 500 µg/mL), SF (IZD = 13 mm, MIC = 1000 µg/mL), SA (IZD = 12 mm, MIC > 1000 µg/mL), MCF (IZD = 13 mm, MIC > 1000 µg/mL), no activity against EC, SAB and KA (IZD = 0 mm each, MIC = 0 µg/mL each).	Ghana	[170]
			Agar diffusion method	**Leaf** water extract: activity against EC (IZD = 6.5 mm (original extract) to 11 mm (dilution 10^{8})), and SA (IZD = 6 mm for dilution 10^{0}-10^{10}).	Kenya	[171]
39.		*Senecio discifolius* Oliv.	Liquid dilution method	**Whole plant** *n*-hexane extract: weak activity against BC (MIC = 1 mg/mL), no activity against SA.	Tanzania	[109]
40.		***Vernonia adoensis* Sch. Bip. ex Walp**	Agar disc diffusion assay	**Leaf** ethanol extract: activity against **MA (IZD = 28 ± 1 mm**, MIC = 31 µL/disc), and CG (IZD = 15 ± 1 mm, MIC = 250 µL/disc).	Zimbabwe	[110]

SNo.	FAMILY	PLANT NAME	METHOD OF ANTI-MICROBIAL ACTIVITY TEST EMPLOYED	ANTIMICROBIAL ACTIVITY OF EXTRACT	COUNTRY	BOOK OR JOURNAL REFERENCE
			(500 μg extract / disc)			
			Disc diffusion method	**Leaf** petrol ether extract (1.75 mg/disc): no activity against SA, BS, EC, and PA; dichloromethane extract (4 mg/disc): no activity against SA, BS, EC, and PA; water extract (5 mg/disc): activity against SA (IZD = 20 ± 0 mm), BS (IZD = 10 ± 0 mm), and **EC (IZD = 46.00 ± 1.41 mm)**, no activity against PA.	Tanzania	[115]
41.		*Vernonia cinerea* (L.) Less	Agar diffusion method (1 mg extract / test)	**Leaf** *n*-hexane extract: activity against SA (IZD = 10 ± 0.76 mm, MIC = 1.56 mg/mL), EC (IZD = 11.0 ± 0.57 mm, MIC = 6.25 mg/mL), KP (IZD = 11.8 ± 1.83 mm; MIC = 3.13 mg/mL), PA (IZD = 9.6 ± 0.44 mm, MIC = 3.13 mg/mL), PV (IZD = 10.5 ± 0.50 mm, MIC = 3.13 mg/mL); chloroform extract: active against SA (IZD = 10 ± 0.28 mm, MIC = 3.13 mg/mL), BC (IZD = 12.6 ± 1.36 mm, MIC = 6.25 mg/mL), EC (IZD = 12.0 ± 0.50 mm, MIC = 3.13 mg/mL), KP (IZD = 11.7 ± 0.72 mm, MIC = 3.13 mg/mL), PA (IZD = 12.8 ± 0.72 mm, MIC = 3.13 mg/mL), PV (IZD = 12.0 ± 0.28 mm, MIC = 1.56 mg/mL); ethyl acetate extract: active against SA (IZD = 10.2 ± 0.44 mm, MIC = 6.25 mg/mL), BC (IZD = 13.0 ± 0.76 mm, MIC = 3.13 mg/mL), EC (IZD = 13.5 ± 2.02 mm, MIC = 3.13 mg/mL), KP (IZD = 12.8 ± 0.44 mm, MIC = 3.13 mg/mL), PA (IZD = 12.7 ± 0.72 mm, MIC = 6.25 mg/mL), PV (IZD = 12.8 ± 1.09 mm, MIC = 3.13 mg/mL).	Nigeria	[172]
			Agar diffusion method	**Plant** hot water extract: activity against SM serotype c (MT 5091) and serotype d (OMZ 176) (MIC = 46.9 mg/mL each).	Taiwan	[173]
			Disc diffusion method (one seed per test)	**Seed**: activity against PC (IZD = 1 mm), no activity against BS, EC, and STM.	India	[174]
			Agar diffusion method	**Leaf and stem** ethanol (80%) extract: activity against EC, PA, and SA (MIC = 25 mg/mL each) and BS (MIC = 12.5 mg/mL).	India	[103]

SNo.	FAMILY	PLANT NAME	METHOD OF ANTI-MICROBIAL ACTIVITY TEST EMPLOYED	ANTIMICROBIAL ACTIVITY OF EXTRACT	COUNTRY	BOOK OR JOURNAL REFERENCE
			Disc diffusion assay (250 μg extract / disc), agar and broth dilution methods	**Aerial part** methanol extract: activity against SA and MM (IZD = 8 mm each, MIC = 6.25 mg/mL each), PA and EA (IZD = 10 mm each, MIC = 3.13 mg/mL each), CF (IZD = 14 mm, MIC = 6.25 mg/mL), EC (IZD = 12 mm, MIC = 3.13 mg/mL), AC (IZD = 13 mm, MIC = 6.25 mg/mL), ABA (IZD = 14 mm, MIC = 6.25 mg/mL), BL and *Micrococcus* spp. (IZD = 12 mm each, MIC = 6.25 mg/mL each), SE (IZD = 9 mm, MIC = 3.13 mg/mL), *Erwinia* spp. (IZD = 12 mm, MIC = 3.13 mg/mL), BC (IZD = 17 mm, MIC = 3.13 mg/mL), SRM (IZD = 11 mm, MIC = 3.13 mg/mL), SSP (IZD = 10 mm, MIC = 6.25 mg/mL), YE (IZD = 13 mm, MIC = 3.13 mg/mL), SHS (IZD = 15 mm, MIC = 3.13 mg/mL), no activity against KP, ST, and BS; chloroform extract: activity against SA, BL, and *Micrococcus* spp. (IZD = 11 mm each, MIC = 6.25 mg/mL each), PA, EC, BC, and SHS (IZD = 11 mm each, MIC = 3.13 mg/mL each), CF (IZD = 10 mm, MIC = 6.25 mg/mL), AC, ABA, and MM (IZD = 12 mm each, MIC = 6.25 mg/mL each), SE, *Erwinia* spp and YE (IZD = 10 mm each, MIC = 3.13 mg/mL each), SRM (IZD = 8 mm, MIC = 3.13 mg/mL), SSP (IZD = 9 mm, MIC = 6.25 mg/mL), EA (IZD = 11 mm, MIC = 12.5 mg/mL), no activity against KP, ST, and BS. More activities of diethyl ether, ethyl acetate, and butanol extracts against stated microorganisms see reference.	Malaysia	[175]
			Microdilution method	**Leaf** methanol extract: activity against BC and SA (MIC = 0.31 mg/mL each), and EA (MIC = 0.62 mg/mL).	India	[176]
42.		***Vernonia colorata* (Willd.) Drake var. *colorata***	Microplate alamar blue assay (MABA)	**Root** ethanol extract: activity against MTB (H37Rv) (MIC = 0.5 mg/mL), no activity against PBA (MIC > 0.5 mg/mL).	South Africa	[160]
			Microplate broth dilution method	**Aerial parts** *n*-hexane extract: activity against SA (MIC = 125 μg/mL) and no activity against EF, KP, PA, and MS; dichloromethane extract: activity against SA (MIC = 125 μg/mL), EF (MIC = 250 μg/mL), and **MS (MIC = 31 μg/mL**), no activity against KP and PA; ethylacetate extract: activity	Mozambique	[31]

SNo.	FAMILY	PLANT NAME	METHOD OF ANTI-MICROBIAL ACTIVITY TEST EMPLOYED	ANTIMICROBIAL ACTIVITY OF EXTRACT	COUNTRY	BOOK OR JOURNAL REFERENCE
				against SA and MS (MIC = 125 µg/mL each), no activity against EF, KP, and PA: methanol extract: activity against SA (MIC = 62.5 µg/mL), EF (MIC = 250 µg/mL), and MS (MIC = 125 µg/mL), no activity against KP and PA.		
43		***Vernonia hildebrandtii* Vatke**	Disc diffusion method (0.5 mg extract/disc)	**Leaf and stem** extract: ether fraction active against SA (IZD = 10-15 mm); chloroform fraction active against **SA (IZD = 10-20 mm)**; petrol fraction not active against SA.	Tanzania	[118]
44.	Convolvulaceae	*Ipomoea biflora* (L.) Pers. (syn. *Ipomoea sinensis* (Desr.) Choisy)	Agar-well diffusion method (10 mg extract / well)	**Not specified** methanol extract: activity against BC (IZD = 7 mm), BS and PA (IZD = 3 mm each), BM and KP (IZD = 5 mm), SA and EF (IZD = 6 mm each), ST (IZD = 10 mm, MIC = 2 mg/mL), no activity against SE, ML, EC, and SPT.	India	[177]
45.	Costaceae	*Costus afer* Ker Gawl.	Agar well diffusion method (0.3 mL/well)	**Stem sap**: activity against EC (IZD = 10.00 ± 1.56 mm), SA (IZD = 11.33 ± 1.33 mm), BS (IZD = 12.67 ± 0.88 mm), and PA (IZD = 8.66 ± 0.66 mm).<br>**Leaf sap**: activity against EC (IZD = 9.33 ± 0.33 mm), SA (IZD = 10.00 ± 0.58 mm), BS (IZD = 9.67 ± 0.33 mm), and PA (IZD = 7.33 ± 0.33 mm).	Nigeria	[178]
46.	Crassulaceae	***Bryophyllum pinnatum* (Lam.) Oken**	Broth dilution technique	**Leaf** ethanol extract: activity against SA ATCC 25923 (MIC = 0.78 mg/mL), SA1 (MIC = 1.56 mg/mL), SA2 and PM (MIC = 3.12 mg/mL each), EC ATCC25922 (MIC = 6.25 mg/mL), EC1, EC2 and KP1 (MIC = 12.5 mg/mL each); methanol extract: activity against SA ATCC 25923, and SA1 (MIC = 1.56 mg/mL each), SA2, EC ATCC25922 and PM (MIC = 3.12 mg/mL each), EC1 and KP1 (MIC = 12.5 mg/mL each), EC2 (MIC = 25 mg/mL).	India	[179]
			Agar well diffusion assay (5 mg extract / well)	**Leaf** methanol extract: resistant against MTB, activity against SA (IZD = 3 mm), no activity against EC, KP, PA, and ST; *n*-hexane fraction: **sensitive against MTB at 25 µg/mL**, no activity against SA, EC, KP, PA, and ST; dichloromethane fraction: **sensitive against MTB at 25 µg/mL**, activity	Nigeria	[27]

SNo.	FAMILY	PLANT NAME	METHOD OF ANTI-MICROBIAL ACTIVITY TEST EMPLOYED	ANTIMICROBIAL ACTIVITY OF EXTRACT	COUNTRY	BOOK OR JOURNAL REFERENCE
			and proportion method	against EC and ST (IZD = 5 mm), KP (IZD = 3 mm), PA (IZD = 7 mm) all at 5 mg, no activity against SA; ethyl acetate fraction: sensitive against MTB at 40 µg/mL, resistant at 25 µg/mL, activity against SA (IZD = 17 mm), EC (IZD = 19 mm), **KP (IZD = 20 mm)**, **PA (IZD = 23 mm)**, and **ST (IZD = 20 mm)** all at 5 mg; water extract: sensitive against MTB at 40 µg/mL, resistant at 25 µg/mL, activity against SA (IZD = 9 mm), and PA (IZD = 10 mm), no activity against EC, KP, and ST.		
			Micro dilution method	**Whole plant** methanol extract: activity against SA (MIC = 256 µg/mL), PA (MIC = 512 µg/mL), and SP (MIC = 64 µg/mL); ethyl acetate extract: activity against SA (MIC = 64 µg/mL), PA (MIC = 128 µg/mL), and **SP (MIC = 32 µg/mL)**; *n*-hexane extract: activity against SA (MIC = 512 µg/mL), PA (MIC = 1024 µg/mL), and SP (MIC = 256 µg/mL).	Cameroon	[53]
47.	Dennstaedtiaceae	***Pteridium aquilinum* (L.) Kuhn**	Agar well method	**Leaf** ethanol extract (100 µg/mL): activity against EC, PV; amd BS (IZD = 18 mm each), **SA (IZD = 20 mm)**, no activity against PA; petroleum ether extract (100 µg/mL): activity against EC and PV (IZD = 16 mm), **SA (IZD = 20 mm)**, **BS (IZD = 22 mm)**, no activity against PA; methanol and chloroform extracts (100 µg/mL): no activity against EC, SA, PV, BS, and PA.	India	[180]
48.	Ebenaceae	***Diospyros mespiliformis* Hochst. ex DC.**	Disc diffusion method (0.5 mg extract/disc)	**Leaf** extract: ether and chloroform fractions active against SA (IZD = 10-15 mm each); petrol fraction not active against SA and EC.	Tanzania	[118]
			Cup plate agar diffusion method (2 mg extract /	**Leaf** water extract: no activity against MS; methanol extract: activity against MS (IZD = 12 ± 0.35 mm, MIC = 167 µg/mL).<br>**Root** water extract: no activity against MS; methanol extract: activity against **MS (IZD = 21.3 ± 0.50 mm**, MIC = 250 µg/mL).	Nigeria	[181]

SNo.	FAMILY	PLANT NAME	METHOD OF ANTI-MICROBIAL ACTIVITY TEST EMPLOYED	ANTIMICROBIAL ACTIVITY OF EXTRACT	COUNTRY	BOOK OR JOURNAL REFERENCE
			hole) and agar-diffusion method			
			Disc diffusion method (20 µL extract / disc)	**Root** petroleum ether extract: activity against SA NCTC 6571, SA E3T, and EC (IZD: ++ each), and PA (IZD: +); chloroform extract: activity against SA NCTC 6571, SA E3T (IZD: +++ each), EC and PA (IZD: ++).	Nigeria	[46]
49.		***Euclea divinorum* Hiern.**	Disc diffusion method (2 mg extract / disc), microdilution	**Bark and leaf** ethanol extract: activity against AN (IZD = 5.5 mm, MIC = 6.2 mg/mL), AI (IZD = 4.6 mm, MIC = 12.5 mg/mL), SM (IZD = 6.0 mm, MIC = 25.0 mg/mL), AA (IZD = 0.0 mm, MIC = 6.3 mg/mL), and PG (IZD = 6.5 mm, MIC = 3.1 mg/mL), no activity against PI (IZD = 0.0 mm).	South Africa	[159]
			Disc-diffusion method (4 mg extract/disc), broth micro-dilution assay	**Root** methanol extract: activity against **SA and multiresistant strains SE 847 (IZD = 24 mm each)**, BC (IZD = 12 mm), MCF (IZD = 18 mm), EC (IZD = 11 mm), PA (IZD = 15 mm), SH 535 (IZD = 16 mm), and **SA NGR (IZD = 26 mm)**; hot water extract: activity against SA (IZD = 16 mm), and multiresistant SE 847 (IZD = 12 mm), and **SA NGR (IZD = 20 mm)**, not active against BC, MCF, EC, PA, and multiresistant strain SH 535.	Yemen	[182]
			Agar dilution method	**Fruit** methanol extract (2000 µg/mL): activity against NG, no activity against BC, SP, SPN SA, ST, STM, SHF, SD, and EC.<br>**Leaf** methanol extract (2000 µg/mL): activity against NG, no activity against BC, SP, SPN SA, ST, STM, SHF, SD, and EC.	Ethiopia	[139]
50.	Euphorbiaceae	***Acalypha fruticosa* Forssk.**	Disc diffusion method (0.5 mg extract /disc)	**Leaf** extract: chloroform fractions active against **SA (IZD = 10-20 mm)** and not active against EC; ether and petrol fractions not active against SA and EC.	Tanzania	[118]

SNo.	FAMILY	PLANT NAME	METHOD OF ANTI-MICROBIAL ACTIVITY TEST EMPLOYED	ANTIMICROBIAL ACTIVITY OF EXTRACT	COUNTRY	BOOK OR JOURNAL REFERENCE
51.		*Croton dichogamus* Pax	Two fold microdilution method	**Stem bark** ethanol extract: activity against MMA and MIP (MIC = 1.25 mg/mL each).	Tanzania	[169]
52.		*Euphorbia heterochroma* Pax	Agar well-diffussion method (200 μg extract / well)	**Milky exudate** petroleum ether fraction: activity against SA, SG, PA and KP (IZD lower than standard antibiotics for all four cases), no activity against EC and PV; dichloromethane fraction (1000 μg/mL): activity against SA (IZD greater than standard antibiotics), SG, PA, and KP (IZD lower than standard antibiotics), no activity against EC and PV; methanol fraction (1000 μg/mL): activity against SA (IZD lower than standard antibiotics), no activity against SG, EC, PV, PA, and KP; residual water fraction (1000 μg/mL): activity against EC (IZD lower than standard antibiotics), no activity against SA, SG, , PV, PA, and KP; direct water extract (1000 μg/mL): activity against SA, EC, PV, and KP (IZD lower than standard antibiotics), PA (IZD equal to the standard antibiotics), no activity against SG.	Ethiopia	[105]
53.		***Euphorbia hirta* L.**	Well diffusion antimicrobial activity (5 μg extract/well)	**Leaf/stem** methanol extract: activity against PA and SA ATC29213 (IZD = 0.5 cm each), and BS (IZD = 1.0 cm); medium activity against SA NCRL, poor activity against EC.	Uganda	[183]
			Agar well diffusion method (10 μg extract / well) and LRP assay	**Leaf** methanol extract: activity against BS (IZD = 15 mm), **EC (IZD = 20 mm)**, and VC (IZD = 10 mm), no activity against SA and EF, 32.02% reduction of MTB H37Rv at 250 μg/mL; *n*-hexane extract: no activity against BS, SA, EC, VC, and EF, 10.57% reduction of MTB H37Rv at 250 μg/mL; ethyl acetate extract: activity against BS (IZD = 12 mm) and EF (IZD = 10 mm), no activity against SA, EC, and VC, 42.24% reduction of MTB H37Rv at 250 μg/mL.	India	[184]

SNo.	FAMILY	PLANT NAME	METHOD OF ANTI-MICROBIAL ACTIVITY TEST EMPLOYED	ANTIMICROBIAL ACTIVITY OF EXTRACT	COUNTRY	BOOK OR JOURNAL REFERENCE
			Disc diffusion method (0.5 mg extract /disc)	**Whole plant** extract: chloroform fraction active against **SA (IZD = 15-20 mm)**; ether and petrol fractions not active against SA.	Tanzania	[118]
54.		***Jatropha curcas* L.**	Tetrazolium microplate assay	**Leaf** methanol (80%) extract: weak activity against MTB (H37Rv) (MIC = 1.6 mg/mL).	Malaysia	[165]
			REMA assay	**Leaf** *n*-hexane extract: no activity against MS and MU (MIC > 250 μg/mL each).	Ghana	[112]
			Agar well method (10 mg extract / well)	**Leaf** methanol extract (50 mg/mL):activity against SA (IZD = 19.3± 1.2 mm), SRM (IZD = 17.3 ± 0.6 mm at 10 mg/mL), BS (IZD = 14.0 ± 0 mm), MP (IZD = 12.0 ± 0 mm); *n*-hexane extract (50 mg/mL): activity against SA (IZD = 13.0± 0 mm), SRM (IZD = 14.3 ± 1.2 mm at 10 mg/mL), BS (IZD = 15.0 ± 0 mm), MP (IZD = 14.3 ± 0.6 mm); ethyl acetate extract (50 mg/mL): activity against **SA (IZD = 21.3± 0.3 mm)**, SRM (IZD = 19.0 ± 1.0 mm at 10 mg/mL), **BS (IZD = 21.7 ± 0.6 mm)**, **MP (IZD = 24.3 ± 1.2 mm)**.	Philippines	[184]
			Tetrazolium microplate assay	**Leaf and stem** ethanol (95%) extract: activity against MTB H37Rv (MIC = 200 μg/mL).	Peru	[185]
			Disc diffusion method	**Leaf** petrol ether extract (3.75 mg/disc): activity against SA and PA (IZD = 8.5 ± 0.7 mm each), BS (IZD = 7.0 ± 0.0 mm), no activity against EC; dichloromethane extract (3.25 mg/disc): activity against EC (IZD = 14.5 ± 0.7 mm), no activity against SA, BS, and PA; water extract (2.125 mg/disc): activity against **SA (IZD = 40 ± 0 mm)**, BS (IZD = 8.5 ± 0.7 mm), **EC (IZD = 39 ± 0 mm)**, and PA (IZD = 15.00 ± 1.41 mm).	Tanzania	[115]
			Agar well diffusion method	**Seed** water and ethanol extracts (50 mg/mL): no activity against SA, BS, and EC.	Somalia	[104]

SNo.	FAMILY	PLANT NAME	METHOD OF ANTI-MICROBIAL ACTIVITY TEST EMPLOYED	ANTIMICROBIAL ACTIVITY OF EXTRACT	COUNTRY	BOOK OR JOURNAL REFERENCE
			(hole diameter: 10 mm)			
			Microbial percent inhibition assay (50 μg extract / well)	**Leaf** methanol extract: activity against SA (IZD = 2 mm), no activity against EC; hexane: activity against EC (IZD = 1 mm), no activity against SA.	Tonga	[102]
55.		***Mallotus oppositifolius* (Geiseler) Müll. Arg.**	Disc diffusion assay (6 mg extract/disc), agar dilution assay	**Leaf** methanol extract: activity against EC 25922, EC 35922, and PA 27853 (IZD = 16 mm each, MIC ≥ 10 mg/mL each), EA 29751 and EBC HGY18 (IZD = 19 mm each, MIC ≥ 10 mg/mL each), EA 13048, KP HGY6 (IZD = 14 mm each, MIC ≥ 10 mg/mL each), KP HGY19 and AB HGY13 (IZD = 18 mm each, MIC ≥ 10 mg/mL each), KO U103 and SRM HGY4 (IZD = 15 mm each, MIC ≥ 10 mg/mL each), SMR HYG10 (IZD = 13 mm, MIC ≥ 10 mg/mL), AB HGY12 (IZD = 17 mm, MIC ≥ 10 mg/mL), EH 9790 (IZD = 17 mm, MIC = 1.25 mg/mL), ***Enterococcus* sp. P054 (IZD = 20 mm**, MIC = 2.5 mg/mL each), **SA 25923 and SA U127 (IZD = 26 mm each**, MIC = 2.5 mg/mL each), **SSP (IZD = 26 mm**, MIC ≤ 0.3 mg/mL).<br>**Seed** methanol extract: activity against EC 25922 (IZD = 15 mm, MIC = 5 mg/mL), EC 35922 and EBC HGY18 (IZD = 13 mm each, MIC ≥ 10 mg/mL each), EA 29751 and SMR HYG10 (IZD = 11 mm each, MIC = 10 mg/mL each), EA 13048 (IZD = 12 mm, MIC ≥ 10 mg/mL), KP HGY19 (IZD = 11 mm, MIC ≥ 10 mg/mL), KP HGY6 (IZD = 9 mm, MIC ≥ 10 mg/mL), KO U103 (IZD = 10 mm, MIC ≥ 10 mg/mL), SRM HGY4 (IZD = 13 mm, MIC = 10 mg/mL), PA 27853 (IZD = 14 mm, MIC = 10 mg/mL), AB HGY13 (IZD = 17 mm, MIC = 2.5 mg/mL), AB HGY12 (IZD = 17 mm, MIC = 5 mg/mL), EH 9790 (IZD = 18 mm, MIC = 5 mg/mL), ***Enterococcus* sp. P054 and SA U127 (IZD = 20 mm each**, MIC = 1.25 mg/mL each), **SA 25923 (IZD = 22 mm**, MIC ≤ 0.3 mg/mL), and **SSP (IZD = 25 mm**, MIC ≤ 0.3 mg/mL).	Cameroon	[186]

SNo.	FAMILY	PLANT NAME	METHOD OF ANTI-MICROBIAL ACTIVITY TEST EMPLOYED	ANTIMICROBIAL ACTIVITY OF EXTRACT	COUNTRY	BOOK OR JOURNAL REFERENCE
			Disc-diffusion (1 mg/disc) and microdilution assays	**Root** petroleum ether extract: no activity against EC, ML, KP, SA, and BS; dichloromethane extract: no activity against EC, ML, KP, SA, and BS; ethanol (80%) extract: activity against EC (IZD = 0.80 ± 0.01 mm, MIC = 0.39 mg/mL), and SA (IZD < 0.1 mm, MIC = 1.56 mg/mL), no activity against ML, KP, and BS.	Nigeria	[187]
				**Leaf** petroleum ether extract: activity against ML (IZD = 0.16 ± 0.00 mm, MIC = 0.195 mg/mL), SA (IZD = 1.89 ± 0.27 mm, MIC < 0.098 mg/mL), and BS (IZD = 0.40 ± 0.01 mm, MIC = 1.560 mg/mL), no activity against EC and KP; dichloromethane extract: activity against EC (bacteriostatic effect, MIC = 3.125 mg/mL), ML (IZD = 0.36 ± 0.01 mm, MIC = 6.250 mg/mL), SA (IZD = 2.36 ± 0.01 mm, MIC < 0.098 mg/mL), and BS (IZD = 0.43 ± 0.01 mm, MIC = 0.780 mg/mL), no activity against KP; ethanol (80%) extract: activity against EC (bacteriostatic effect, MIC = 1.560 mg/mL), ML (IZD = 0.48 ± 0.03 mm, MIC = 0.780 mg/mL), SA (IZD = 0.50 ± 0.00 mm, MIC = 0.390 mg/mL), and BS (IZD = 0.36 ± 0.02 mm, MIC = 0.780 mg/mL), no activity against KP.		
56.		*Ricinus communis* L	Disc diffusion method (0.5 mg extract /disc)	**Root** extract: ether fraction active against SA (IZD = 10 – 15 mm); petrol and chloroform fractions not active against SA.	Tanzania	[118]
			REMA assay	**Leaf** *n*-hexane extract: activity against MS (MIC = 125 μg/mL), no activity against MU (MIC > 250 μg/mL).	Ghana	[112]
57.		***Sapium ellipticum* (Hochst.) Pax** (syn. *Shirakiopsis elliptica* (Hochst.) Esser)	Standard broth dilution method	**Stem bark** water extract: activity against SL and KP (MIC = 6.2 mg/mL); ethyl acetate extract: activity against SL and KP (MIC = 12.5 mg/mL).	Tanzania	[188]
			Microdilution assay	**Stem bark** methanol extract: activity against clinical isolates of EC (MIC = 8 mg/mL), PA (MIC = 4 mg/mL), PS (MIC = 1 mg/mL), PV (MIC = 2 mg/mL), and SSP (MIC = 0.12 mg/mL).	Cameroon	[189]

SNo.	FAMILY	PLANT NAME	METHOD OF ANTI-MICROBIAL ACTIVITY TEST EMPLOYED	ANTIMICROBIAL ACTIVITY OF EXTRACT	COUNTRY	BOOK OR JOURNAL REFERENCE
			Agar well diffusion technique (10 mg extract/well)	**Leaf** water extract: activity against SE (IZD = 17 mm), **PM (IZD = 25 mm)**, PA (IZD = 14 mm), and EC (IZD = 16 mm); ethanol extract: activity against **SE (IZD = 24 mm)**, **PM (IZD = 32 mm)**, **PA (IZD = 30 mm)**, and **EC (IZD = 38 mm)**; methanol extract: activity against **SE (IZD = 34 mm)**, PM (IZD = 4 mm), PA (IZD = 13 mm), and EC (IZD = 19 mm); hexane, chloroform, and diethyl ether extracts: no activity against SE, PM, PA, and EC at conc. between 3.01-100 mg/mL.	Nigeria	[190]
58.		***Spirostachys africana* Sond.**	Microdilution assay	**Root/stem** *n*-hexane extract: no activity against BS, EC, KP, and SA; ethanol extract: activity against SA (MIC = 3.13 mg/mL), no activity against BS, EC, and KP; water extract: no activity against BS, EC, KP, and SA.	South Africa	[121]
			Micro-dilution technique	**Stem bark** ethanol extract: activity against SA, VC, and SHD (MIC = 156 μg/mL each), SHF (MIC = 312 mg/mL), and SHB (MIC = 625 μg/mL), no activity against ST, EC, and SHS (MIC > 625 μg/mL each).	South Africa	[54]
			Microdilution assay	**Bark** petroleum ether extract: activity against **EC (MIC = 0.10 μg/mL), BS (MIC = 0.78 μg/mL), KP (MIC = 1.56 μg/mL)**, and **SA (MIC = 0.39 μg/mL)**; dichloromethane extract: activity against **BS (MIC = 3.13 μg/mL), EC and KP (MIC = 1.56 μg/mL each), and SA (MIC = 3.13 μg/mL)**; ethanol extract: activity against **SA (MIC = 0.01 μg/mL)**, **BS (MIC = 0.20 μg/mL)**, **EC and KP (MIC = 1.56 μg/mL each**); water extract: activity against BS **(MIC = 0.39 μg/mL), EC (MIC = 0.78 μg/mL), KP (MIC = 1.56 μg/mL),** and **SA (MIC = 0.78 μg/mL)**.	South Africa	[24]
59.	Lamiaceae	***Plectranthus barbatus* Andrews** (syn. *Coleus barbatus* (Andrews) Benth. Ex G. Don, syn.	Disc diffusion method	**Leaf** petrol ether extract (3.25 mg/disc): activity against SA (IZD = 10.0 ± 1.41 mm), BS (IZD = 7.0 ± 0.0 mm), and PA (IZD = 19.00 ± 1.41 mm), no activity against EC; dichloromethane extract (1.75 mg/disc): activity against **PA (IZD = 21.00 ± 1.41 mm)**, no activity against SA, BS, and EC;	Tanzania	[115]

SNo.	FAMILY	PLANT NAME	METHOD OF ANTI-MICROBIAL ACTIVITY TEST EMPLOYED	ANTIMICROBIAL ACTIVITY OF EXTRACT	COUNTRY	BOOK OR JOURNAL REFERENCE
		Plectranthus comosus Sims)		water extract (1.5 mg/disc): activity against SA (IZD = 14.00 ± 1.41 mm), BS (IZD = 9.00 ± 2.82 mm), no activity against EC, and PA.		
			Agar well diffusion method (12.5 mg extract/well)	**Root** hot water extract: activity against SA (IZD = 18.67 ± 1.20 mm) and **BS (IZD = 25.33 ± 0.88 mm)**, no activity against EC (IZD < 8.00 mm).	Kenya	[191]
			Proportion method	**Stem and root** ethanol (95%) extracts: no activity against MTB.	Rwanda	[192]
60.		*Hoslundia opposita* Vahl.	Liquid dilution method	**Leaf** methanol extract: activity against BC (MIC = 0.5 mg/mL), no activity against SA.	Tanzania	[109]
			Well-diffusion antimicrobial activity (5 μg extract/well)	**Leaf/stem** methanol extract: activity against SA ATC29213, SA NCRL, SE, SF, and BS (IZD = 1.0 cm each).	Uganda	[183]
61.		*Hyptis pectinata* (L.) Poit.	Agar plate diffusion assay (2 mg extract/hole)	**Whole plant** methanol extract: activity against SA (IZD = 6 mm), and BS (IZD = 9 mm), no activity against EC and PA.	Mexico	[193]
62.		*Ocimum suave* Willd.	Agar dilution streak method	**Leaf** and **stem** methanol extracts: no activity against BS, MS, PA, SG and SA at concentration of 1000 μg/mL.	Rwanda	[194]
			Agar well diffusion method (12.5 mg extract/well)	**Leaf** hot water extract: no activity against SA, BC, and EC (IZD < 8.0 mm).	Kenya	[191]

SNo.	FAMILY	PLANT NAME	METHOD OF ANTI-MICROBIAL ACTIVITY TEST EMPLOYED	ANTIMICROBIAL ACTIVITY OF EXTRACT	COUNTRY	BOOK OR JOURNAL REFERENCE
			Well-diffusion antimicrobial activity (5 µg extract/well)	**Leaf/stem/root** methanol extract: activity against SE (IZD = 0.5 cm); medium activity against BS.	Uganda	[183]
			Microbroth dilution	**Leaf/stem/flower** chloroform extract: activity against SA (MIC = 62 µg/mL), and SF (MIC = 125 µg/mL), EC (MIC = 500 µg/mL), and PA (MIC = 500 µg/mL); petroleum ether extract: activity against EC (MIC = 250 µg/mL), PA (MIC = 250 µg/mL), SA and SF (MIC = 500 µg/mL each).	Uganda	[183]
			Broth dilution method	**Essential oil from leaves and terminal branchlets**: activity against EC (MIC = 900 µg/mL), ML (MIC = 700 µg/mL), SA (MIC = 800 µg/mL), and SC (MIC = 500 µg/mL).	Tanzania	[195]
63.		*Ocimum gratissimum* L. (syn. *Ocimum suave* Willd.)	Agar disc diffusion method (impregnation with 300 mg extract/mL), BACTEC MGIT™ 960 system	**Plant** methanol extract: activity against MK, MTB, MF, and MS (complete inhibition at a conc. of 2 mg/mL), ST and SA (IZD = 8.66 mm each), EC (IZD = 7 mm), PA (IZD = 7.66 mm), and KP (IZD = 6.66 mm).	Kenya	[196]
64.	Leguminosae (Fabaceae)	*Abrus precatorius* L.	Microplate alamar blue assay (MABA)	**Aerial parts** and **seeds** ethanol extract: activity against MS (MIC = 1 mg/mL), MTB (H37Rv) (MIC = 0.25 mg/mL), no activity against PBA (MIC > 0.5 mg/mL).	South Africa	[160]
			Broth microdilution method	**Not specified** methanol extract: weak activity against MTB (MIC = 1838 µg/mL), high activity against MB (MIC = 500 µg/mL).	Nigeria	[197]

SNo.	FAMILY	PLANT NAME	METHOD OF ANTI-MICROBIAL ACTIVITY TEST EMPLOYED	ANTIMICROBIAL ACTIVITY OF EXTRACT	COUNTRY	BOOK OR JOURNAL REFERENCE
			Disc diffusion method (disc diaemeter: 6 mm)	**Leaf** hot water extract (10 g/L): activity against EC (IZD = 6.3 ± 1.3 mm), SA (IZD = 15.7 ± 0.5 mm), and BS (IZD = 8.7 ± 1.3 mm).<br>**Bark** hot water extract (10 g/L): activity against EC (IZD = 7.2 ± 0.8 mm), SA (IZD = 10.8 ± 1.0 mm), and BS (IZD = 10.7 ± 1.2 mm),	Kenya	[116]
			Agar diffusion method	**Leaf** ethanol (80%) extract: activity against SA (MIC = 25 mg/mL each), no activity against EC, PA, and BS.	India	[103]
			Disc diffusion assay (4 mg extract / disc)	**Root** ethanol (70%) extract: activity against SA (IZD = 9 ± 1 mm), BS (IZD = 12 ± 1 mm), BM (IZD = 8 ± 1 mm), EC (IZD = 2 – 19 mm for four strains), PA (IZD = 10 – 14 mm for two strains), PP (IZD = 4 ± 0 mm), KPC (IZD = 15 ± 2 mm), and CG (5 ± 1 mm); petroleum ether fraction: activity against SA (IZD = 6 ± 0 mm), BS (IZD = 8 ± 1.0 mm), BM (IZD = 7 ± 0 mm), EC (IZD = 0 – 9 mm for four strains), PP (IZD = 9 ± 1 mm), KPC (IZD = 10 ± 1.5 mm), and CG (6 ± 1 mm), no activity against PA (IZD = 0 mm for two strains); chloroform fraction: activity against SA (IZD = 14 ± 2 mm), BS (IZD = 11 ± 1 mm), BM (IZD = 7 ± 0 mm), EC (IZD = 8-14 mm for four strains), PA (IZD = 7-9 mm for two strains), PP (IZD = 10 ± 0 mm), KPC (IZD = 10 ± 1 mm), CG (5 ± 1 mm); ethanol fraction: activity against SA (IZD = 14 ± 1 mm), BS (IZD = 9 ± 1 mm), BM (IZD = 7 ± 1 mm), EC (IZD =5-13 mm for four strains), PA (IZD = 1-6 mm for two strains), PP (IZD = 6 ± 1 mm), KPC (IZD = 5 ± 1 mm), and CG (7 ± 1 mm); distilled water fraction: activity against SA (IZD = 1 ± 0 mm), BS (IZD = 2 ± 0 mm), BM (IZD = 1 ± 0 mm), EC (IZD = 1-6 mm for four strains), PP (IZD = 1 ± 0 mm), and KPC (IZD = 1 ± 0 mm), no activity against CG and two strains of PA (IZD = 0 mm each).	India	[198]
			Antimicrobial sensitivity test	**Root** ethanol extract: sensitive against SA, EC, and SAE, non-sensitive against SP and KP.<br>**Stem bark** ethanol extract: sensitive against SA, EC, and SP, non-sensitive against KP and SAE.	Nigeria	[199]

SNo.	FAMILY	PLANT NAME	METHOD OF ANTI-MICROBIAL ACTIVITY TEST EMPLOYED	ANTIMICROBIAL ACTIVITY OF EXTRACT	COUNTRY	BOOK OR JOURNAL REFERENCE
				**Leaf** ethanol extract: sensitive against SA, SAE, and KP, non-sensitive against EC and SP.		
65.		*Acacia brevispica* Harms	Liquid dilution method	**Leaf** *n*-hexane extract: activity against BC (MIC = 0.5 mg/mL), no activity against SA and KP; methanol extract: weak activity against BC (MIC = 1 mg/mL), no activity against SA.	Tanzania	[109]
66.		***Acacia mellifera* (M. Vahl) Benth.**	Disc diffusion method (0.5 mg extract /disc)	**Stem bark** extract: ether fraction active against SA (IZD = 10-15 mm); chloroform and petrol fractions not active against SA.	Tanzania	[118]
			Agar dilution method	**Whole plant** *n*-hexane extract (20 mg/mL): activity against SP (IZD = 13 mm), SA (IZD = 10 mm), EC (IZD = 18 mm), no activity against PF and KP; ethyl acetate extract (20 mg/mL): activity against SP (IZD = 12 mm), EC (IZD = 10 mm), no activity against SA, PF and KP; ethanol extract (20 mg/mL): activity against SP (IZD = 15 mm), SA (IZD = 13 mm), **EC (IZD = 36 mm)**, no activity against PF and KP; methanol extract (20 mg/mL): activity against SP (IZD = 14 mm), SA (IZD = 10 mm), PF (IZD = 12 mm), KB (IZD = 18 mm), and **EC (IZD = 20 mm)**.	India	[200]
67.		***Acacia nilotica* (L.) Delile**<br><br>(see also Rather et al., 2015 for more information)	Disc diffusion method (0.5 mg extract /disc)	**Whole plant** extract: petrol fraction active against **SA and EC (IZD = 20-25 mm each)**; ether and chloroform fractions active against SA (IZD = 10-15 mm) and not active against EC.	Tanzania	[118]
			Microtitre dilution technique	**Bark** methanol (80%) extract: activity against SA, BS, ST, EC (MIC = 1.875 mg/50 µL each), and PA (MIC = 0.9375 mg/50 µL).	Kenya	[152]
			Antimycobacterial susceptibility test (BACTEC	**Stem bark** methanol extract: slight activity against MK (3656 GUs at 1 mg/mL extract, 0 GUs at 2 mg/mL), MTB (19,613 GUs at 1 mg/mL, 0 GUs	Kenya	[153]

SNo.	FAMILY	PLANT NAME	METHOD OF ANTI-MICROBIAL ACTIVITY TEST EMPLOYED	ANTIMICROBIAL ACTIVITY OF EXTRACT	COUNTRY	BOOK OR JOURNAL REFERENCE
			MGIT™ 960 system)	at 2 mg/mL), activity against MF (0 mg/mL at 0.5 mg/mL), MS (0 GUs at 0.5 mg/mL).		
			Antitubercular activity	**Fruit** methanol (70%) extract: activity against MTB BCG strain (MIC = 1250 μg/mL); gradient column chromatographic separation of extract → fractions:FR.1 (100% *n*-hexane): activity against MTB BCG strain (MIC = 1468 μg/mL), FR.2, FR.3, FR.4: activity against MTB BCG strain (MIC = 78 μg/mL each), FR.5: activity against MTB BCG strain (MIC = 156 μg/mL), FR.6 (most polar): activity against MTB BCG strain (MIC = 312 μg/mL).	Nigeria	[201]
			Agar diffusion technique	**Leaf** dichloromethane extract: activity against SA and BC (MIC = 1000 μg/mL each), no activity against MCF, EC, and PA; methanol extract: activity against SA (MIC = 500 μg/mL), BC (MIC = 250 μg/mL), and MCF (MIC = 1000 μg/mL), no activity against EC and PA; water extract: activity against SA, BC, and MCF (MIC = 500 μg/mL each), and PA (MIC = 1000 μg/mL), no activity against EC.	Yemen	[36]
			Bacteria assay on agar plates	**Root** methanol extract: activity against BC, BP, BS, MCK, SA, and EBC (MIC = 0.5 mg/mL each), EA, EC, and PV (MIC = 1 mg/mL each), and SRM (MIC = 5 mg/mL); acetone extract: activity against BC, BP, BS, MCK, SA, EBC, and EA (MIC = 0.5 mg/mL each), EC (MIC = 1 mg/mL), and PV (MIC = 5 mg/mL), no activity against SRM; water extract: activity against BC (MIC = 0.5 mg/mL), BP, BS, MCK, SA, and EBC (MIC = 1 mg/mL each), and EA (MIC = 50 mg/mL), no activity against EC, PV, and SRM.	Zimbabwe	[202]
			Agar dilution method	**Seed** methanol extract (2000 μg/mL): activity against NG, no activity against BC, SA SP, SPN ST, SHF, STM, SD, and EC.	Ethiopia	[139]

SNo.	FAMILY	PLANT NAME	METHOD OF ANTI-MICROBIAL ACTIVITY TEST EMPLOYED	ANTIMICROBIAL ACTIVITY OF EXTRACT	COUNTRY	BOOK OR JOURNAL REFERENCE
68.		*Acacia polyacantha* Willd. (syn. *Acacia polyacantha* subsp. *campylacantha*)	Micro-titre plate dilution assay	**Leaf** dichloromethane:methanol extract: activity against SM (MIC = 2.67 mg/mL), SS (MIC = 1 mg/mL), PG and FN (MIC = 1 mg/mL each), no activity against LA; water extract: activity against SS (MIC = 3 mg/mL), PG and FN (MIC = 8 mg/mL each), no activity against SM, LA, and LC.<br>**Stem** dichloromethane:methanol extract: activity against SM and FN (MIC = 2.67 mg/mL each), SS (MIC = 1 mg/mL), and PG (MIC = 0.5 mg/mL), no activity against LA and LC; water extract: activity against SM and SS (MIC = 2 mg/mL each), PG (MIC = 8 mg/mL), FN (MIC = 4 mg/mL), no activity against LA and LC.	South Africa	[143]
69.		*Afrormosia angolensis* (Baker) de Wild. (syn. *Pericopsis angolensis* (Baker) Meeuwen)	Well diffusion assay	**Bark** cold water extract: activity against *Shigella* spp. (MIC = 1.526 mg/mL), ST and EC (MIC = 0.781 mg/mL each), ethanol extract: activity against *Shigella* spp. (MIC = 12.5 mg/mL), ST (MIC = 0.196 mg/mL), and EC (MIC = 0.098 mg/mL); water extract obtained by the traditional extraction method: activity against *Shigella* spp. (MIC = 0.0915 mg/mL), ST (MIC = 0.781 mg/mL), no activity against EC.	Zimbabwe	[203]
70.		*Afzelia quanzensis* Welw.	Plate-hole diffusion method	**Bark** methanol and water extracts: no activity against SE and SA.	South Africa	[148]
71.		*Albizia anthelmintica* Brongn.	Micro-titre dilution technique	**Root/bark** methanol (80%) extract: activity against SA (MIC = 1.875 mg/50 µL), BS, ST, EC (MIC = 3.75 mg/50 µL each), and PA (MIC = 1.875 mg/50 µL).	Kenya	[152]
			Antimycobacterial susceptibility test (BACTEC MGIT™ 960 system)	**Bark** methanol extract: slight activity against MK (1,702 GUs at 1 mg/mL extract, 0 GUs at 2 mg/mL), activity against MTB (1,603 GUs at 0.5 mg/mL, 0 GUs at 1 mg/mL), MF (701 GUs at 0.5 mg/mL, 0 GUs at 1 mg/mL), and MS (14,761 GUs at 0.5 mg/mL, 0 GUs at 1 mg/mL).	Kenya	[196]

SNo.	FAMILY	PLANT NAME	METHOD OF ANTI-MICROBIAL ACTIVITY TEST EMPLOYED	ANTIMICROBIAL ACTIVITY OF EXTRACT	COUNTRY	BOOK OR JOURNAL REFERENCE
			Microplate serial dilution method	**Root** ethanol extract: activity against MA (MIC = 0.156 mg/mL).<br>**Leaf** ethanol extract: activity against MA (MIC = 0.625 mg/mL).	Botswana	[204]
			Disc diffusion method (disc diaemeter: 6 mm)	**Bark** hot water extract (10 g/L): activity against EC (IZD = 6.3 ± 0.3 mm), SA (IZD = 6.8 ± 0.3 mm), and BS (IZD = 11.3 ± 1.8 mm).	Kenya	[116]
72.		*Albizia versicolor* Oliv.	Microdilution method	**Bark** dichloromethane:methanol extract: activity against BBA, SE, EC, PA (MIC = 1000 μg/mL each), SA MR (MIC = 2000 μg/mL), SA GMR (MIC = 3000 μg/mL), and PBA (MIC = 8000 μg/mL); not active against BBL and SA (MIC > 8000 μg/mL each).<br>**Bark** aqueous extract: activity against PBA (MIC = 130 μg/mL), PA (MIC = 2000 μg/mL), SA MR (MIC = 8000 μg/mL); no activity against BBA, BBL, SA GMR, SA, EC (MIC > 8000 μg/mL each).	South Africa	[129]
			Two-fold serial dilution microplate method	**Leaf** acetone extract: no activity against EC, EF, and PA (MIC > 6.3 mg/mL).	South Africa	[205]
			Plate-hole diffusion and broth microdilution methods	**Bark** methanol extract: no activity against SE and SA; water extract: activity against SE (MIC = 3.25 mg/mL), no activity against SA.	South Africa	[148]
73.		***Bauhinia reticulata* DC.**	Disc diffusion method (0.5 mg extract /disc)	**Whole plant** extract: petrol fraction active against **SA (IZD = 15-20 mm)**, and EC (IZD = 10-15 mm); ether fraction active against **EC (IZD = 15-20**	Tanzania	[118]

SNo.	FAMILY	PLANT NAME	METHOD OF ANTI-MICROBIAL ACTIVITY TEST EMPLOYED	ANTIMICROBIAL ACTIVITY OF EXTRACT	COUNTRY	BOOK OR JOURNAL REFERENCE
				**mm)** and not active against SA; chloroform fraction not active against SA and EC.		
74.		*Cajanus cajan* (L.) Druce	Liquid dilution streak method	**Leaf** methanol extract: activity against BS, MS and SA, no activity against PA and SG at concentrations of 1000 µg/mL.<br>**Stem** methanol extract: no activity against BS, MS, PA, SG and SA at concentrations of 1000 µg/mL.	Rwanda	[96]
			Disc diffusion method	**Leaf** methanol extract: strong activity against NG, NM, weak activity against SP, no activity against SA at concentrations of 1000 µg/mL.	Rwanda	[206]
			Tetrazolium microplate assay	**Leaf and stem** ethanol (95%) extract: activity against MTB H37Rv and MDR MTB (MIC = 100 µg/mL each).	Peru	[185]
			Serial two-fold dilution method	**Leaf** ethanol (80%) extract: activity against SE, SA, BS, and PV (MIC = 2.50 mg/mL each), PA (MIC = 10 mg/mL), no activity against EC (MIC > 20 mg/mL); supercritical fluid extract: activity against SE, SA, and PV (MIC = 0.039 mg/mL each), BS (MIC = 0.02 mg/mL), PA and EC (MIC > 2.5 mg/mL each).	China	[207]
			Micro-broth dilution method	**Leaf** ethanol (95%) extract: activity against SE, SA, and BS (MIC = 2.50 mg/mL each); chloroform fraction: activity against SE, SA, and BS (MIC = 1.25 mg/mL).	China	[208]
			Disc diffusion method (1 seed per test)	**Seed**: activity against BS (IZD = 4 mm), EC and PC (IZD = 2 mm each), and STM (IZD = 1 mm).	India	[174]
75.		***Cassia abbreviata* Oliv**.	Broth microdilution method	**Stem bark** water and organic extracts: hot ethanol extract activity against **NG (MIC = 46.88 µg/mL)**, PA (MIC = 93.75 µg/mL), and KP (187.5 µg/mL); water extract: activity against NG and PA (MIC = 93.75 µg/mL each), no activity against KP; trichloromethane extract: activity against **KP (MIC = 46.88**	Zambia	[25]

SNo.	FAMILY	PLANT NAME	METHOD OF ANTI-MICROBIAL ACTIVITY TEST EMPLOYED	ANTIMICROBIAL ACTIVITY OF EXTRACT	COUNTRY	BOOK OR JOURNAL REFERENCE
				**μg/mL)**, no activity against NG and PA; dichloromethane and water extracts: activity against NG , PA, and KP (93.75 μg/mL each); cold ethanol extract: activity against **NG (MIC = 46.88 μg/mL)**, PA (MIC = 93.75 μg/mL), no active against KP; cold water extract: activity against NG (MIC = 93.75 μg/mL), **PA (MIC = 46.88 μg/mL)**, no activity against KP.		
			Microplate broth dilution method	**Stem bark** *n*-hexane extract: activity against KP (MIC = 250 μg/mL) and MS (MIC = 62.5 μg/mL each), no activity against SA, EF, and PA; dichloromethane extract: activity against EF and KP (MIC = 250 μg/mL each) and MS (MIC = 62.5 μg/mL each), no activity against SA and PA; ethylacetate exract: activity against EF and KP (MIC = 250 μg/mL each), and **MS (MIC = 31 μg/mL)**, no activity against SA and PA; methanol extract: activity against **SA (MIC = 15 μg/mL)**, EF (MIC = 250 μg/mL), KP and PA (MIC = 125 μg/mL each), and **MS (MIC = 31 μg/mL)**.	Mozambique	[31]
			Disc diffusion method (0.5 mg extract /disc)	**Root** extract: ether fraction active against **SA (IZD = 15-20 mm)**; petrol and chloroform fractions not active against SA.	Tanzania	[118]
			Well-diffusion assay (10 mg/mL)	**Bark** methanol extract (10 mg/mL): activity against SA (IZD = 3.00 ± 0.41 mm), *Streptococcus* group A (IZD = 4.50 ± 0.58 mm), no activity against EC and PA.<br>**Leaf** methanol extract (10 mg/mL): activity against *Streptococcus* group A (IZD = 2.00 ± 0.00 mm), no activity against SA, EC and PA.<br>**Root** methanol extract (10 mg/mL): activity against SA (IZD = 1.50 ± 0.41 mm), *Streptococcus* group A (IZD = 2.13 ± 0.63 mm), PA (IZD = 3.00 ± 0.41 mm), no activity against EC.	Zimbabwe	[128]
			Bacteria assay on agar plates	**Bark** methanol extract: activity against BC and EA (MIC = 1 mg/mL each), BP, BS, MCK, SA, and EBC (MIC = 0.5 mg/mL each), EC, PV, and SRM (MIC = 5 mg/mL each); acetone extract: activity against BC and EBC (MIC = 1	Zimbabwe	[202]

SNo.	FAMILY	PLANT NAME	METHOD OF ANTI-MICROBIAL ACTIVITY TEST EMPLOYED	ANTIMICROBIAL ACTIVITY OF EXTRACT	COUNTRY	BOOK OR JOURNAL REFERENCE
				mg/mL), BP, BS, MCK, and SA (MIC = 0.5 mg/mL each), EA, EC, and PV (MIC = 5 mg/mL each), no activity against SRM; water extract: activity against BP, MCK, and SA (MIC = 1 mg/mL each), BS, and EBC (MIC = 5 mg/mL each), no activity against BC, EBC, EC, PV, and SRM.		
76.		***Cassia mimosoides* L.** (syn. *Chamaecrista mimosoides* (L.) Greene)	Agar well diffusion method (2.5 mg extract/well)	**Whole plant** chloroform extract: activity against SA (IZD = 15.32 ± 0.52 mm); methanol extract: activity against SA (IZD = 14.50 ± 0.52 mm); water extract: activity against **SA (IZD = 30.00 ± 1.46 mm)**.	Kenya	[209]
			Liquid dilution method	**Whole plant** ethanol (80%) extract: activity against MF (MIC = 10 mg/mL), SA and SP (MIC = 5 mg/mL), not active against EC, KP, PV, PA, and SPT (MIC > 10 mg/mL).	Rwanda	[210]
			Diffusion method (10 mg extract/disc)	**Leaf** ethanol extract: activity against EC (IZD = 6.0 mm), *Salmonella* spp. (IZD = 3.0 mm), and *Shigella* spp (IZD = 1.0 mm); water extract: activity against SA and *Salmonella* spp. (IZD = 2.0 mm each), *Shigella* spp. (IZD = 4.0 mm).	Nigeria	[211]
77.		*Cassia singueana* Delile (syn. *Senna singueana* (Delile) Lock)	Disc diffusion method (disc diaemeter: 6 mm)	**Leaf** hot water extract (10 g/L): activity against EC (IZD = 8.5 ± 0.4 mm), SA (IZD = 10.8 ± 0.8 mm), and BS (IZD = 11.5 ± 0.4 mm).	Kenya	[116]
78.		***Dichrostachys cinerea* (L.)**	Microdilution method	**Twigs** dichloromethane:methanol extract: activity against **SE (MIC = 0.190 μg/mL)**, EC (MIC = 750 μg/mL), BBA, BBL, PBA (MIC = 1000 μg/mL each), SA GMR, SA MR (MIC = 2000 μg/mL each); water extract: activity against PBA (MIC = 4000 μg/mL), no activity against BBA, BBL, SA GMR, SA MR, SA, EC, and PA (MIC > 8000 μg/mL each).	South Africa	[129]
			Micro-titre plate dilution assay	**Leaf** dichloromethane:methanol extract: activity against SM (MIC = 1.33 mg/mL), SS (MIC = 4 mg/mL), LA (MIC = 6.67 mg/mL), LC (MIC = 5.33 mg/mL), PG (MIC = 2.67 mg/mL), FN (MIC = 2 mg/mL); water extract:	South Africa	[143]

SNo.	FAMILY	PLANT NAME	METHOD OF ANTI-MICROBIAL ACTIVITY TEST EMPLOYED	ANTIMICROBIAL ACTIVITY OF EXTRACT	COUNTRY	BOOK OR JOURNAL REFERENCE
				activity against SM and PG (MIC = 2 mg/mL each), SS (MIC = 4 mg/mL), LA and LC (MIC = 8 mg/mL each), no activity against FN.<br>**Stem** dichloromethane:methanol extract: activity against SM and PG (MIC = 2 mg/mL each), SS (MIC = 3.33 mg/mL), LC (MIC = 4 mg/mL), and FN (MIC = 0.67 mg/mL), no activity against LA; water extract: activity against PG (MIC = 2 mg/mL), and FN (MIC = 8 mg/mL), no activity against SM, SS, LA, and LC.		
			Microplate Alamar Blue Assay (MABA)	**Fruit** methanol extract: no activity against MTB H37Ra and MTB H37Rv (MIC > 2048 μg/mL).	Cameroon	[212]
			BACTEC MGIT™ 960 system and disc diffusion method (15 mg extract/disc)	**Root** methanol extract: considerable activity against MTB H37Rv no activity against EC, SA, and CA (IZD = 6 ± 0 mm, MIC not applicable).	Kenya	[111]
			Microplate serial dilution method	**Root** ethanol extract: activity against MA (MIC = 0.156 mg/mL), no activity against BS, BC, EC, SA, and PA.	Botswana	[110]
			Cup-plate agar diffusion (3 mg extract/cup)	**Stem bark** water extract: activity against BC (IZD = 9.5 mm), CBD (IZD = 10.0 mm), KP (IZD = 9.0 mm), PM (IZD = 10.5 mm), PA (IZD = 12.0 mm), ST (IZD = 12.0 mm), and SP (IZD = 9.5 mm); methanol extract: activity against BC and KP (IZD = 10.5 mm each), CBD (IZD = 11.0 mm), PM, PA, and ST (IZD = 10.0 mm each), and SP (IZD = 11.5 mm); ethyl acetate extract: activity against BC and PA (IZD = 8 mm each), KP (IZD = 9.5 mm), PM (IZD = 7.0 mm), and SP (IZD = 10.0 mm); no activity against CBD and ST.	Cameroon	[213]

SNo.	FAMILY	PLANT NAME	METHOD OF ANTI-MICROBIAL ACTIVITY TEST EMPLOYED	ANTIMICROBIAL ACTIVITY OF EXTRACT	COUNTRY	BOOK OR JOURNAL REFERENCE
			Well-diffusion assay (well diameter: 4 mm)	**Leaf** methanol extract (10 mg/mL): activity against SA (IZD = 2.13 ± 0.25 mm), *Streptococcus* group A (IZD = 2.50 ± 0.58 mm), PA (IZD = 4.75 ± 0.65 mm), no activity against EC.<br>**Root** methanol extract: activity against PA (IZD = 4.00 ± 0.82 mm), no activity against SA, *Streptococcus* group A and EC.	Zimbabwe	[128]
			Bacteria assay on agar plates	**Fruit** methanol extract: activity against BC, BP, BS, MCK, and SA (MIC = 1 mg/mL each), EBC, EC, and SRM (MIC = 5 mg/mL each), no activity against EA and PV; acetone extract: activity against BC and EBC (MIC = 5 mg/mL), BP, BS, MCK, SA, and EC (MIC = 1 mg/mL each), no activity against EA, PV, and SRM; water extract: no activity against BC, BP, BS, MCK, SA, EBC, EA, EC, PV, and SRM.	Zimbabwe	[202]
79.		*Dichrostachys glomerata* (Forssk.) Chiov. (syn. *Dichrostachys cinerea* (L.))	Well diffusion antimicrobial activity (100 µg extract/well)	**Seed** dichloromethane/methanol (1:1) extract: activity against STM (IZD = 13 mm), LM (IZD = 16 mm), EC (IZD = 14 mm), and SA (IZD = 18 mm).	Cameroon	[214]
			Liquid micro-broth dilution	**Fruit** methanol extract: activity against various strains of EC (MIC = 256-1024 µg/mL), various strains of EA (MIC = 128-1024 µg/mL), various strains of EBC (MIC = 1024 µg/mL for each strain), various strains of KP (MIC = 512-1024 µg/mL), various strains of PS (MIC = 256–1024 µg/mL), and various strains of PA (MIC = 512-1024 µg/mL).	Cameroon	[215]
80		*Dichrostachys cinerea* (L.) Wight et Arn. subsp. *africana* Brenan & Brummitt	Plate-hole diffusion method	**Bark** methanol and water extracts: no activity against SE and SA.	South Africa	[148]

SNo.	FAMILY	PLANT NAME	METHOD OF ANTI-MICROBIAL ACTIVITY TEST EMPLOYED	ANTIMICROBIAL ACTIVITY OF EXTRACT	COUNTRY	BOOK OR JOURNAL REFERENCE
81.		*Entada abyssinica* A. Rich	Microdilution method	**Stem bark** ethanol extract: activity against ST (MIC = 12.5 mg/mL), EC (MIC = 12.5 mg/mL), VC (MIC = 0.8 mg/mL), no activity against KP.	Tanzania	[108]
			Well diffusion antimicrobial activity (5 µg extract/well)	**Leaf/stem** methanol extract: activity against SA (IZD = 1.5 cm for ATC29213 and 1.0 cm for NCRL), SE and SF (IZD = 1.0 cm each), and BS (IZD = 2.0 cm)	Uganda	[183]
			Microbroth dilution	**Bark** chloroform fraction: poor activity against EC (MIC = 500 µg/mL), PA (MIC = 1000 µg/mL), SA (MIC = 500 µg/mL), and SF (MIC = 500 µg/mL); petroleum ether fraction: activity against EC (MIC = 250 µg/mL), PA (MIC = 62 µg/mL), SA (MIC = 125 µg/mL), and SF (MIC = 62 µg/mL).	Uganda	[183]
			Agar diffusion test (250 µg extract/test)	**Stem bark** acetone extract: activity against EC, *Proteus* sp., and SA (IZD = 8 mm each).	Cameroon	[216]
			Serial dilution assay	**Stem bark** methanol extract: activity against SA (MIC = 0.25-1 mg/mL), *Enterococci* (MIC = 0.06-2 mg/mL), PA (MIC = 0.13-0.5 mg/mL), *Klebsiella* (MIC = 1-8 mg/mL), and *Salmonella* (MIC = 0.5-2 mg/mL), no activity against MBC, MBA, MI, MT, and MTB at extract conc. of 0.5–2 mg/mL.	Kenya	[217]
			Two fold microdilution method	**Stem bark** ethanol extract: activity against MMA and MIP (MIC = 2.5 mg/mL each).	Tanzania	[169]
82.		*Entada leptostachya* Harms	Broth dilution method	**Tuber** methanol (70%) extract (10 g/L): activity against BC (MIC = 125 mg/mL), PA (MIC = 250 mg/mL each), no activity against ML and EC.	Kenya	[164]

SNo.	FAMILY	PLANT NAME	METHOD OF ANTI-MICROBIAL ACTIVITY TEST EMPLOYED	ANTIMICROBIAL ACTIVITY OF EXTRACT	COUNTRY	BOOK OR JOURNAL REFERENCE
			Disc diffusion method (disc diaemeter: 6 mm)	**Root** hot water extract (10 g/L): activity against EC (IZD = 10.2 ± 0.2 mm), SA (IZD = 11.5 ± 0.4 mm), and BS (IZD = 8.8 ± 0.2 mm).	Kenya	[116]
83.		*Erythrina abyssinica* DC.	Liquid dilution method	**Stem bark** methanol extract: activity against BC (MIC = 0.125 mg/mL), and SA (MIC = 0.25 mg/mL).	Tanzania	[109]
			Microtitre plate method	**Root bark** total crude methanol extract: activity against MTB H37RV and MBA (MIC = 0.39 ± 0.0 mg/mL each), MTB TMC 331 (MIC = 2.35 ± 1.11 mg/mL); serial methanol extract: activity against MTB H37RV (MIC = 4.69 ± 2.20 mg/mL), MTB TMC 331 (MIC = 1.17 ± 0.55 mg/mL), and MBA (MIC = 1.0 ± 0.0 mg/mL); chloroform extract: activity against MTB H37RV (MIC = 0.89 ± 0.83 mg/mL), MTB TMC 331 (MIC = 0.39 ± 0.0 mg/mL), and MBA (MIC = 0.3 ± 0.13 mg/mL).	Uganda	[218]
			Agar disc diffusion method (impregnation with 300 mg extract/mL), BACTEC MGIT™ 960 system	**Plant** methanol extract: activity against MK, MTB, MF, and MS (complete inhibition at a conc. of 2 mg/mL), ST and KP (IZD = 8.00 mm each), SA (IZD = 8.66 mm), EC (IZD = 7.33 mm), and PA (IZD = 10.33 mm).	Kenya	[196]
			Well diffusion antimicrobial activity (5 µg extract/well)	**Leaf/stem** methanol extract: activity against SF (IZD = 3.0 cm), medium activity against SA (ATC29213 and NCRL), SE, and BS; poor activity against EC.<br>**Stem bark** methanol extract (1 mg/mL): activity against SA (IZD = 1.0 cm for ATC29213 and 1.0 cm for NCRL), SE (IZD = 1.5 cm), SF (IZD = 3.0 cm), BS (IZD = 1.0 cm)	Uganda	[183]

SNo.	FAMILY	PLANT NAME	METHOD OF ANTI-MICROBIAL ACTIVITY TEST EMPLOYED	ANTIMICROBIAL ACTIVITY OF EXTRACT	COUNTRY	BOOK OR JOURNAL REFERENCE
			Microbroth dilution	**Leaf** chloroform fraction: activity against EC (MIC = 125 μg/mL), SA (MIC = 62 μg/mL), and SF (MIC = 62 μg/mL); poor activity against PA (MIC = 500 μg/mL); petroleum ether fraction: activity against EC and PA (MIC = 250 μg/mL each), and SF (MIC = 62 μg/mL); poor activity against SA (MIC = 500 μg/mL).<br>**Bark** chloroform fraction: activity against PA (MIC = 125 μg/mL), SA (MIC = 250 μg/mL), and SF (MIC = 62 μg/mL); poor activity against EC (MIC = 500 μg/mL); petroleum ether fraction: activity against PA (MIC = 250 μg/mL), SA (MIC = 125 μg/mL), and SF (MIC = 62 μg/mL); poor activity against EC (MIC = 500 μg/mL).	Uganda	[183]
			Agar dilution streak method	**Leaf** methanol extract: no activity against BS, MS, PA, SG and SA at concentrations of 1000 μg/mL	Rwanda	[96]
			Broth dilution method	**Root/bark** methanol (70%) extract: no activity against ML, BC, PA, and EC.	Kenya	[164]
84.		*Peltophorum africanum* Sond.<br><br>(see also Mongalo, 2013 for more information)	Resazurin microplate assay (REMA)	**Bark** acetone extract: no activity against MTB (H37Ra) (MIC > 100 ± 2.6 μg/mL) and MTB 2 (MIC > 100 ± 10.2 μg/mL).	South Africa	[26]
			Broth microdilution method	**Root** methanol extract: activity against SE (MIC = 0.50 mg/mL), and SA (MIC = 2 mg/mL); water extracts: activity against SE and SA (MIC = 3.61 mg/mL each).	South Africa	[148]
85.		***Tamarindus indica* L.**	Tetrazolium microplate assay	**Fruit** methanol (80%) extract: no activity against MTB (H37Rv) (MIC > 1.6 mg/mL)	Malaysia	[165]
			Agar diffusion technique	**Flowers** dichloromethane extract: activity against SA (MIC = 125 mg/mL), and BC (MIC = 500 μg/mL), no activity against MCF, EC, and PA; methanol extract: activity against **SA (MIC = 25 μg/mL)**, BC, EC, and PA (MIC = 125	Yemen	[36]

SNo.	FAMILY	PLANT NAME	METHOD OF ANTI-MICROBIAL ACTIVITY TEST EMPLOYED	ANTIMICROBIAL ACTIVITY OF EXTRACT	COUNTRY	BOOK OR JOURNAL REFERENCE
				μg/mL each), no activity against MCF; water extract: activity against SA, BC, MCF, EC, and PA (MIC = 125 μg/mL each).		
			Broth microdilution assay	**Fruit** acetone extract: activity against MS (MIC = 0.31 mg/mL), MF (MIC = 0.15 mg/mL each), and MA (MIC = 0.078 mg/mL).	Cameroon	[219]
			Broth microdilution assay	**Fruit** methanol extract: activity against two MDR EC strains MC4100 (MIC = 1024 μg/mL) and W311O (MIC = 512 μg/mL), MDR EA strain EA298 (MIC = 1024 μg/mL), two MDR EBC strains ECCI69 and BM67 (MIC = 1024 μg/mL each), MDR KP strain K2 (MIC = 1024 μg/mL), no activity against six MDR EC strains, five MDR EA strains, MDR EC strain BM47, four MDR KP strains, three MDR PC strains, and two MDR PA strains.	Cameroon	[220]
			Agar dilution method	**Leaf** ethanol (95%) extract: activity against five EC strains (MIC = 15.62 mg/mL)	Thailand	[221]
86.		*Tephrosia purpurea* subsp. *leptostachya* (DC.) Brummit	Disc diffusion method (1 seed per test)	**Seed**: activity against BS and PC (IZD = 1 mm each), and STM (IZD < 1 mm), no activity against EC.	India	[174]
87.	Loganiaceae	*Strychnos spinosa* Lam.	Microdilution method	**Fruit** dichloromethane:methanol extract: activity against BBA (MIC = 40 μg/mL), SE (MIC = 250 μg/mL), PA (MIC = 500 μg/mL), EC (MIC = 1000 μg/mL), BBL (MIC = 1500 μg/mL), SA MR, and PBA (MIC = 2000 mg/mL each), SA GMR and SA (MIC = 4000 μg/mL each).<br>**Fruit** water extract: activity against SA GMR (MIC = 1000 μg/mL), SA MR and SA (MIC = 8000 μg/mL each); no activity against BBA, BBL, SE, PBA, EC, and PA.	South Africa	[129]

SNo.	FAMILY	PLANT NAME	METHOD OF ANTI-MICROBIAL ACTIVITY TEST EMPLOYED	ANTIMICROBIAL ACTIVITY OF EXTRACT	COUNTRY	BOOK OR JOURNAL REFERENCE
			Broth microdilution assay	**Mature edible fruit** methanol (70%) extract: activity against STM (MIC = 25 mg/mL), BC (MIC = 1.6 mg/mL), and KP (MIC = 12.5 mg/mL), no activity against SP and PI.	South Africa	[222]
			Broth serial microdilution method with tetrazolium violet	**Leaf** acetone extract; activity against SA, EF (MIC = 0.16 mg/mL each), BC ((MIC = 0.32 mg/mL), and EC (MIC = 0.32-1.25 mg/mL); methanol extract: activity against EC (MIC = 0.63 mg/mL), no activity against SA, BC, and EF; dichloromethane/methanol extract: activity against SA. EF, and EC (MIC = 0.63 mg/mL each), no activity against BC; alkaloid extract: activity against SA (MIC = 0.16 mg/mL), BC (MIC ≥ 1.25 mg/mL), EF (MIC = 0.32 mg/mL), and EC (MIC = 0.16-1.25 mg/mL).<br>**Leaf** *n*-hexane fraction: activity against SA (MIC = 0.08 mg/mL), BC (MIC = 0.63-1.25 mg/mL), EF (MIC = 0.32 mg/mL), and EC (MIC = 0.08-1.25 mg/mL); chloroform fraction: activity against SA (MIC = 0.08 mg/mL), BC (MIC = 0.63 mg/mL), EF (MIC = 0.16 mg/mL), and EC (MIC = 0.16-0.32 mg/mL); ethyl acetate fraction: activity against SA (MIC = 0.16 mg/mL), BC (MIC = 0.63-1.25 mg/mL), EF (MIC = 0.16-0.32 mg/mL), and EC (MIC = 0.16-1.25 mg/mL); *n*-butanol fraction: activity against SA (MIC = 0.08-0.16 mg/mL), BC (MIC = 0.63 mg/mL), EF (MIC = 1.25 mg/mL), and EC (MIC = 0.63 mg/mL); water fraction: no activity against SA, BC, EF, and EC.	Nigeria	[223]
88.	Malvaceae	***Adansonia digitata* L.**	Broth microdilution assay	**Mature edible fruit** methanol (70%) extract: activity against STM (MIC = 6.3 mg/mL), SP (MIC = 6.3 mg/mL), BC (MIC = 1.6 mg/mL), and KP (MIC = 12.5 mg/mL), no activity against PI.	South Africa	[222]
			Broth microdilution assay	**Leaf** water extract: activity against SA, EC, KO, and SAE (MIC = 1.56 mg/mL each), and SHS (MIC = 6.25 mg/mL); methanol (100%) extract: activity against SA (MIC = 0.78 mg/mL), EC, SAE, and SHS (MIC = 1.56 mg/mL each), and KO (MIC = 0.39 mg/mL).	South Africa	[224]

SNo.	FAMILY	PLANT NAME	METHOD OF ANTI-MICROBIAL ACTIVITY TEST EMPLOYED	ANTIMICROBIAL ACTIVITY OF EXTRACT	COUNTRY	BOOK OR JOURNAL REFERENCE
				**Seed** water and methanol (100%) extracts: no activity against SA, EC, KO, SAE, and SHS (MIC > 12.5 mg/mL).<br>**Pulp** water and methanol (100%) extracts: no activity against SA, EC, KO, SAE, and SHS (MIC > 12.5 mg/mL).		
			Broth microdilution assay	**Fruit** acetone extract: activity against MS and MF (MIC = 1.25 mg/mL each), and MA (MIC = 0.31 mg/mL).<br>**Leaf** acetone extract: activity against MS, MF, and MA (MIC = 1.25 mg/mL each).	Cameroon	[219]
			Broth microdilution assay	**Leaf** methanol extract: activity against seven MDR EC strains (MIC = 256-1024 μg/mL), five MDR EA strains (MIC = 128 – 1024 μg/mL), three MDR EBC strains (MIC = 512 – 1024 μg/mL), five strains MDR KP (MIC = 128-1024 μg/mL), two MDR strains PS (MIC = 1024 μg/mL each), no activity against EC ATCC10536, EA EA27, PC ATCC 29914, and PA PA01 and PA124.	Cameroon	[220]
			Rapid INT colorimetric assay	**Fruit** methanol extract: activity against MDR EC strain ATCC10536 (MIC = 512 μg/mL), MDR EA strain EA298 (MIC = 256 μg/mL), MDR KP strain K2 (MIC = 1024 μg/mL), MDR PS Strain PS29645 (MIC = 256 μg/mL), no activity against seven MDR EC strains, five MDR EA strains, four MDR KP strains, two MDR PS strains, and three EBC strains.	Cameroon	[220]
			Serial dilution microplate bioassay	**Stem bark** dichloromethane extract: activity against SA MR (MIC = 0.156 mg/mL), SA ATCC6538, PA, and SE (MIC > 2.5 mg/mL each), no activity against EF; methanol extract: activity against SA ATCC6538, EF, and SE (MIC = 1.25 mg/mL each), and SA MR (MIC = 0.078 mg/mL), no activity against PA; water/ethanol extract: active against Sa ATCC6538 (MIC = 2.5 mg/mL), EF (MIC = 0.625 mg/mL), SA MR (MIC = 0.078 mg/mL), no active against PA.	Bénin	[225]

SNo.	FAMILY	PLANT NAME	METHOD OF ANTI-MICROBIAL ACTIVITY TEST EMPLOYED	ANTIMICROBIAL ACTIVITY OF EXTRACT	COUNTRY	BOOK OR JOURNAL REFERENCE
			Microdilution assay	**Bark** petroleum ether extract: activity against BS and EC (MIC = 3.125 mg/mL each), KP (MIC = 6.25 mg/mL), and Sa (MIC 12.5 mg/mL); dichloromethane extract: activity against BS, KP, and SA (MIC = 3.125 mg/mL each), and EC (MIC = 1.56 mg/mL); ethanol extract: activity against BS (MIC = 0.78 mg/mL), EC and KP (MIC = 3.125 mg/mL each), and SA (MIC = 1.56 mg/mL); water extract: activity against EC, KP, and SA (MIC = 3.125 mg/mL), no activity against BS (MIC < 12.5 mg/mL).	South Africa	[226]
			Agar well diffusion method (hole diameter: 10 mm)	**Root** ethanol extract (50 mg/mL): activity against **SA (IZD = 25 mm)**, no activity against EC.	Somalia	[104]
89.		*Grewia bicolor* Juss.	Antimicrobial sensitivity test	**Root** ethanol extract: sensitive against SA, EC, SP, SAE, and KP.<br>**Stem bark** ethanol extract: sensitive against SA, EC, and KP, non-sensitive against SP and SAE.<br>**Leaf** ethanol extract: sensitive against EC, SAE, and KP, non-sensitive against SA and SP.	Nigeria	[199]
90.		***Hibiscus micranthus* L.f.**	Agar hole-plate diffusion method (5 mg extract/hole) , agar dilution method	**Bark** methanol extract: activity against AV (IZD = 6.6 mm, MIC = 5 mg/mL), no activity against SM.<br>Wood methanol extract: activity against SM (IZD = 1.75 mm), no activity against AV.	Tanzania	[227]
			Agar-well diffusion method (hole diameter: 6 mm), macrotube	**Leaf** methanol (80%) extract (800 μg/mL): activity against **SA (IZD = 22.67 ± 1.202 mm**, MIC = 2.5 mg/mL), SPN (IZD = 13.00 ± 1.528 mm, MIC = 0.625 mg/mL), KP (IZD = 16.33 ± 1.202 mm, MIC = 2.5 mg/mL), no activity against SP, PA, EC, and PM.	Ethiopia	[228]

SNo.	FAMILY	PLANT NAME	METHOD OF ANTI-MICROBIAL ACTIVITY TEST EMPLOYED	ANTIMICROBIAL ACTIVITY OF EXTRACT	COUNTRY	BOOK OR JOURNAL REFERENCE
			serial dilution method			
91.		*Hibiscus surattensis* L.	Microdilution method	**Leaf** dichloromethane:methanol extract: activity against BBA (MIC = 750 µg/mL), SE, PBA. EC, and PA (MIC = 1000 µg/mL each), SA and SA GMR (MIC = 2000 µg/mL each), SA MR (MIC = 4000 µg/mL), and BBL (MIC = 8000 µg/mL).<br>**Leaf** aqueous extract: activity against SA GMR (MIC = 2000 µg/mL), SA MR (MIC = 4000 µg/mL), and PBA (MIC = 8000 µg/mL); no activity against BBA, BBL, SA, SE, EC, PA (MIC > 8000 µg/mL each).	South Africa	[129]
92.		*Pavonia urens* Cav.	Broth microdilution method	**Leaf** ethanol extract: no activity against EF, SA, EC, and PA.	Ethiopia	[124]
93.		*Sida rhombifolia* L. var. *rhombifolia* (syn. *Sida rhombifolia* L.)	Microplate alamar blue assay (MABA)	**Stem** and **leaf** ethanol extract: activity against PBA (MIC > 500 mg/mL), no activity against MTB (H37Rv) (MIC > 1 mg/mL) and MS (MIC = 1 mg/mL).	South Africa	[160]
		***Sida rhombifolia* L.**	Broth microdilution method	**Root** ethanol extract: no activity against EF, SA, EC, and PA.	Ethiopia	[124]
			Disc diffusion method C	**Root** petroleum ether extract: activity against EC (IZD = 16 ± 1 mm), SA (IZD = 16 ± 2 mm), PA (IZD = 10 ± 0 mm), and STM (IZD = 16.5 ± 0.5 mm); chloroform extract: activity against EC (IZD = 17 ± 0 mm), SA (IZD = 18 ± 1 mm), PA (IZD = 13.8 ± 0 mm), and STM (IZD = 14.5 ± 1.5 mm); methanol extract: activity against **EC (IZD = 20 ± 0 mm)**, SA (IZD = 15 ± 2 mm), PA (IZD = 16 ± 0 mm), and STM (IZD = 17.5 ± 2 mm).	Ethiopia	[229]

SNo.	FAMILY	PLANT NAME	METHOD OF ANTI-MICROBIAL ACTIVITY TEST EMPLOYED	ANTIMICROBIAL ACTIVITY OF EXTRACT	COUNTRY	BOOK OR JOURNAL REFERENCE
			Agar diffusion method	**Root** ethanol (80%) extract: activity against EC (MIC = 25 mg/mL), BS (MIC = 1.56 mg/mL), and SA (MIC = 6.25 mg/mL), no activity against PA.	India	[103]
94.		***Sida serratifolia* R. Wilczek & Steyaert**	Disc diffusion method (0.5 mg extract /disc)	**Leaf** extract: ether and chloroform fractions active against **SA and EC (IZD = 15-20 mm for each combination)**; petrol fraction not active against SA and EC.	Tanzania	[118]
95.		*Waltheria indica* L.	Liquid dilution method	**Shoot** *n*-hexane extract: activity against BC (MIC = 0.5 mg/mL), weak activity against SA (MIC = 1 mg/mL).	Tanzania	[109]
			Microdilution method	**Root** dichloromethane:methanol extract: activity against PA (MIC = 250 μL/mL), BBA (MIC = 310 μL/mL), BBL (MIC = 500 μL/mL), SE and EC (MIC = 1000 μL/mL each), SA (MIC = 2000 μL/mL), SA MR (MIC = 3000 mg/mL), and SA GMR (MIC = 4000 μL/mL); water extract: activity against SA MR (MIC = 2000 μL/mL); no activity against BBA, BBL, SA GMR, SA, SE, PBA, EC, PA (MIC > 8000 μL/mL each).	South Africa	[129]
			Microplate serial dilution method (500 μg extract / disc)	**Leaf** ethanol extract: no activity against BS, BC, EC, SA, and PA.	Botswana	[204]
			Antimicrobial sensitivity test	**Root** ethanol extract: sensitive against SA, EC, SP, SAE, and KP.<br>**Stem bark** ethanol extract: sensitive against SP, SAE EC, and KP, non-sensitive against SA.<br>**Leaf** ethanol extract: sensitive against SA, EC, and KP, non-sensitive against SAE and SP.	Nigeria	[199]
96.	Meliaceae	***Trichilia emetica* Vahl**	Micro-titer plate dilution technique	**Leaf** dichloromethane:methanol (1:1) extract: activity against SA, SA MR and SA GMR (MIC = 0.40 mg/mL each), SE (MIC = 0.20 mg/mL), and **PA**	southern Africa	[32]

SNo.	FAMILY	PLANT NAME	METHOD OF ANTI-MICROBIAL ACTIVITY TEST EMPLOYED	ANTIMICROBIAL ACTIVITY OF EXTRACT	COUNTRY	BOOK OR JOURNAL REFERENCE
				**(MIC = 0.03 mg/mL)**; water extract: no activity against SA, SA MR, SA GMR, SE, and PA (MIC > 16 mg/mL each).		
			Serial microplate dilution method	**Leaf** *n*-hexane extract: activity against SA and EC (MIC = 2.50 mg/mL each), PA (MIC = 1.25 mg/mL), and EF (MIC = 0.30 mg/mL); dichloromethane extract: activity against SA (MIC = 1.25 mg/mL), EC (MIC = 0.60 mg/mL), PA (MIC = 1.25 mg/mL), and EF (MIC = 0.08 mg/mL); acetone extract: activity against SA (MIC = 0.60 mg/mL), EC and **PA (MIC = 0.40 mg/mL each)**, and EF (MIC = 0.26 mg/mL).	South Africa	[134]
			Microplate broth dilution method	**Seed** *n*-hexane extract: activity against SA (MIC = 250 μg/mL), EF and MS (MIC = 125 μg/mL each), no activity against KP and PA; dichloromethane extract: activity against SA (MIC = 250 μg/mL), EF and MS (MIC = 125 μg/mL each), and **PA (MIC = 31 μg/mL)**, no activity against KP; ethylacetate extract: activity against SA (MIC = 250 μg/mL), EF and PA (MIC = 125 μg/mL each), no activity against KP and MS: methanol extract: activity against PA and MS (MIC = 125 μg/mL each), no activity against SA, EF, and KP.	Mozambique	[31]
			Agar dilution method	**Fruit** methanol extract (2000 μg/mL): activity against BC, NG, SA, and ST, no activity against SP, SPN, SHF, STM, SD, and EC.	Ethiopia	[139]
		Trichilia emetica Vahl *subsp. emetica*	MIC assay	**Leaf** dichloromethane:methanol extract: activity against KP (MIC = 1.67 mg/mL), MC (MIC = 1.40 mg/mL), MS (MIC = 2.67 mg/mL each), and SA (MIC = 0.83 mg/mL); water extract: activity against KP, MC, MS, and SA (MIC = 8.00 mg/mL each).	South Africa	[230]
97.	Menispermaceae	***Cissampelos pareira* L.**	Agar diffusion method	**Leaf and stem** ethanol (80%) extract: activity against EC, PA, and SA (MIC = 25 mg/mL each), and BS (MIC = 12.5 mg/mL).	India	[103]

SNo.	FAMILY	PLANT NAME	METHOD OF ANTI-MICROBIAL ACTIVITY TEST EMPLOYED	ANTIMICROBIAL ACTIVITY OF EXTRACT	COUNTRY	BOOK OR JOURNAL REFERENCE
			BACTEC MGIT™ 960 system and disc diffusion method (15 mg extract/disc)	**Root** methanol extract: considerable activity against MTB H37Rv, activity against EC (IZD = 15.3 ± 0.3 mm, MIC = 234 μg), SA (IZD = 11.0 ± 1.0 mm, MIC = 117 μg), and CA (IZD = 11.7 ± 0.3 mm, MIC = 468 μg).	Kenya	[111]
			Disc diffusion test (5 mg extract / disc)	**Root** methanol extract: activity against **SA (IZD = 20 ± 0.6 mm)**, STM (IZD = 17 ± 0.7 mm), KP (IZD = 14 ± 0.6 mm), and EC (IZD = 9 ± 0.6 mm), no activity against PV and SPN.	Kenya	[231]
98.	Moraceae	*Milicia excelsa* (Welw.) C.C. Berg	Agar diffusion method (0.3125 - 10 mg extract / well)	**Leaf** methanol extract: activity against diverse isolates of SA (IZD = 11-16 mm at 10 mg extract/well, no activity at conc. below 1.25 mg extract/well).	Nigeria	[232]
99.	Moringaceae	***Moringa oleifera* Lam.**	Agar well diffusion method	**Leaf** methanol (95%) extract: activity against BC (IZD = 10.7 ± 0.6 mm), and KP (IZD = 9.7 ± 0.6 mm), no activity against SA, SE, EC, and PA.	India	[167]
			Two folds broth microdilution method	**Leaf** petroleum ether extract: activity against MIP and MMA (MIC = 3.13 mg/mL each); ethyl acetate: activity against MIP (MIC = 1.51 mg/mL) and MM (MIC = 0.37 mg/mL); methanol extract: activity against MIP (MIC = 1.51 mg/mL) and MM (MIC = 3.13 mg/mL).<br>**Stem bark** petroleum ether extract: activity against MIP and MMA (MIC = 3.13 mg/mL each); ethyl acetate: activity against MIP (MIC = 3.13 mg/mL) and MM (MIC = 1.51 mg/mL); methanol extract: activity against MIP and MM (MIC = 3.13 mg/mL each).<br>**Root bark** petroleum ether extract: activity against MIP (3.13 mg/mL) and MMA (MIC = 6.25 mg/mL); ethyl acetate: activity against MIP (MIC = 1.51 mg/mL) and MM (MIC = 6.25 mg/mL); methanol extract: activity against MIP (MIC = 3.13 mg/mL) and MM (MIC = 6.25 mg/mL).	Tanzania	[233]

SNo.	FAMILY	PLANT NAME	METHOD OF ANTI-MICROBIAL ACTIVITY TEST EMPLOYED	ANTIMICROBIAL ACTIVITY OF EXTRACT	COUNTRY	BOOK OR JOURNAL REFERENCE
				**Seed** petroleum ether extract: activity against MIP (MIC = 1.51 mg/mL) and MMA (MIC = 3.13 mg/mL); ethyl acetate: activity against MIP and MMA (MIC = 0.37 mg/mL each); methanol extract: activity against MIP and MM (MIC = 1.51 mg/mL each).		
			Agar diffusion method	**Stem bark** ethanol (80%) extract: activity against PA (MIC = 25 mg/mL), BS and SA (MIC = 12.5 mg/mL), no activity against EC.	India	[103]
			Agar-well diffusion method (20 mg extract / well)	**Leaf** water extract: activity against SA (IZD = 7.3±0.3 mm), SE (IZD = 12.3±0.6 mm), BC (IZD = 7.7±0.4 mm), and PA (IZD = 9.6±0.3 mm), no activity against KP, EC, SAE, and PV; butanol extract: activity against SA (IZD = 10.3±0.3 mm), SE (IZD = 14.0±0.0 mm), BC (IZD = 9.0±0.6 mm), KP (IZD = 6.3±0.3 mm) and PA (IZD = 11.6±0.3 mm), no activity against EC, SAE, and PV; ethyl acetate extract: activity against SA (IZD = 13.6±0.3 mm), SE (IZD = 16.0±0.5 mm), BC (IZD = 10.2±0.7 mm), KP (IZD = 6.6±0.3 mm), EC (IZD = 7.0±0.0 mm); SAE (IZD = 6.6±0.3 mm), PV (IZD = 6.3±0.3 mm), and PA (IZD = 13.3±0.3 mm); chloroform extract: activity against SA (IZD = 11.0±0.5 mm), SE (IZD = 9.0±0.5 mm), and BC (IZD = 7.4±0.3 mm), no activity against KP, EC, SAE, PV, and PA.	Sudan	[234]
			Agar well diffusion method (10 mg extract / well), two-fold serial broth method	Comprehensive testing of various extracts (*n*-hexane, ethyl acetate, methanol, and distilled water) of many parts of the plant (leaf, stem, flower, fruit in four various stages of ripening, seed) against gram positive (BC, BS, SA, SE, ML, and EF) and gram negative (EC, ST, SPT, PA, KP, and SRM) bacterial strains.<br>Ethyl acetate extract showed maximum activity against all tested bacterial strains, followed by methanol, *n*-hexane and distilled water extracts in descending order.<br>Highest antimicrobial activities per extraction agent: **Leaf** fresh *n*-hexane extract: activity against EC (IZD = 11 mm, MIC = 8 mg/mL); **mature fruit**	India	[235]

SNo.	FAMILY	PLANT NAME	METHOD OF ANTI-MICROBIAL ACTIVITY TEST EMPLOYED	ANTIMICROBIAL ACTIVITY OF EXTRACT	COUNTRY	BOOK OR JOURNAL REFERENCE
				fresh ethyl acetate extract: activity against **SRM (IZD = 22 mm**, MIC = 2 mg/mL); mature fruit hot distilled water extract: activity against **SA (IZD = 27 mm**, MIC = 0.125 mg/mL); **stem** hot methanol extract: activity against **SA (IZD = 22 mm)**.		
			Inhibition halo technique (10 µg extract / disc)	**Essential oil from leaves**: activity against BS (IZD = 10.7±1.2 mm), EC (IZD = 6.7 ± 0.6 mm), and PA (IZD = 8.0 ± 1.3 mm), no activity against SA.	Mozambique	[236]
			Agar diffusion technique (4 mg extract/disc), tube dilution technique	**Seed** chloroform extract: activity against EA, SA, and PA (IZD = 6 mm each, MIC > 4 mg/mL each), *Shigella* sp. (IZD = 6 mm, MIC = 2 mg/mL), EC (IZD = 9 mm, MIC > 4 mg/mL), STM (IZD = 10 mm, MIC = 1 mg/mL), and ST (IZD = 6 mm, MIC = 2 mg/mL); ethanol extract (400 mg/mL): activity against EA, *Shigella* sp., PA, and ST (IZD = 6 mm each, MIC > 4 mg/mL each), SA (IZD = 11 mm, MIC = 4 mg/mL), EC (IZD = 9 mm, MIC > 4 mg/mL), and STM (IZD = 11 mm, MIC > 4 mg/mL).<br>**Leaf** chloroform extract (400 mg/mL): activity against EA, SA, *Shigella* sp., and PA (IZD = 6 mm each, MIC > 4 mg/mL each), EC (IZD = 8 mm, MIC = 2 mg/mL), STM (IZD = 10 mm, MIC = 1 mg/mL), and ST (IZD = 8 mm, MIC = 2 mg/mL); methanol extract (400 mg/mL): activity against EA (IZD = 7 mm, MIC = 2 mg/mL), SA (IZD = 9 mm, MIC = 2 mg/mL), *Shigella* sp. (IZD = 6 mm, MIC = 2 mg/mL), PA and EC (IZD = 8 mm each, MIC > 4 mg/mL each) STM and ST (IZD = 6 mm each, MIC > 4 mg/mL each).	Nigeria	[237]
100.	Myricaceae	*Myrica salicifolia* Hochst. ex A. Rich.	Agar dilution method	**Stem bark** methanol extract (2000µg/mL): activity against BC, NG, SA, and SD, no activity against SP, SPN ST, STM, SHF, and EC.	Ethiopia	[139]
101	Myrtaceae	*Eucalyptus globulus* Labill.	In vitro screening	**Leaf** methanol extract: no activity against MA (MIC >500 µg/mL) and MS (MIC > 500 µg/mL).	n.d	[101]

SNo.	FAMILY	PLANT NAME	METHOD OF ANTI-MICROBIAL ACTIVITY TEST EMPLOYED	ANTIMICROBIAL ACTIVITY OF EXTRACT	COUNTRY	BOOK OR JOURNAL REFERENCE
			REMA assay	**Leaf** ethanol extract: activity against MS (MIC = 125 μg/mL) and MU (MIC = 250 μg/mL).	Cameroon	[112]
102		***Psidium guajava* L.**	Tetrazolium microplate assay	**Leaf** methanol (80%) extract: no activity against MTB (H37Rv) (MIC > 1.6 mg/mL)	Malaysia	[165]
			MIC assay	**Leaf** dichloromethane:methanol extract: activity against KP and MC (MIC = 1.00 mg/mL each), MS (MIC = 2.00 mg/mL), and SA (MIC = 0.50 mg/mL); water extract: activity against KP and MC (MIC = 0.67 mg/mL each), MS (MIC = 4 mg/mL), and SA (MIC = 0.50 mg/mL).	South Africa	[230]
			Paper disc diffusion method	**Leaf** methanolic extract: activity against **EC and BS (MIC = 0.78 μg/ml each)**, and **SA (MIC = 25 μg/mL)**.	India	[37]
			Disc diffusion method (3 mg extract / disc) + microdilution method	**Leaf** acetone extract: activity against SE MR (IZD = 15 mm, MIC = 0.039 mg/mL), SA MR (IZD = 12 mm, MIC = 0.039 mg/mL), EC (IZD = 6 mm, MIC = 0.312 mg/mL), and EBC (IZD = 4 mm, MIC = 0.312 mg/mL); *n*-hexane extract: no activity against SE MR (IZD = 10 mm), SA MR (IZD = 12 mm), EC, and EBC; methanol extract: activity against **SE MR (IZD = 20 mm**, MIC = 0.078 mg/mL), **SA MR (IZD = 22 mm**, MIC = 0.078 mg/mL), EC (IZD = 10 mm, MIC = 0.312 mg/mL), and EBC (IZD = 13 mm, MIC = 0.625 mg/mL); water extract: no activity against SE MR (IZD = 3 mm), SA MR (IZD = 6 mm), EC, and EBC.	South Africa	[155]
103.		***Syzygium cordatum* Hochst. ex Krauss**	Microdilution method	**Bark** dichloromethane:methanol extract: activity against SA GMR (MIC = 60 μL/mL), BBA, PA (MIC = 250 μL/mL), PA (MIC = 310 μL/mL), EC (MIC = 750 μL/mL), SE (MIC = 1000 μL/mL), SA GMR, SA MR and SA (MIC = 2000 μL/mL each); water extract: activity against BBA and EC (MIC = 1000 μL/mL each), SA GMR, SA MR and SA (MIC = 2000 μL/mL each), SE and	South Africa	[129]

SNo.	FAMILY	PLANT NAME	METHOD OF ANTI-MICROBIAL ACTIVITY TEST EMPLOYED	ANTIMICROBIAL ACTIVITY OF EXTRACT	COUNTRY	BOOK OR JOURNAL REFERENCE
				PA (MIC = 4000 µL/mL each), PBA (MIC = 8000 µL/mL); no activity against BBL (MIC > 8000 µL/mL).		
			MIC assay	**Mature bark** dichloromethane:methanol extract: activity against KP (MIC = 1.00 mg/mL), MC (MIC = 0.83 mg/mL), MS (MIC = 1.33 mg/mL), and SA (MIC = 0.38 mg/mL); water extract: activity against KP and MC (MIC = 0.50 mg/mL each), MS (MIC = 2.00 mg/mL), and SA (MIC = 0.25 mg/mL).	South Africa	[230]
			Disc diffusion assay (200 µg extract / disc) + Broth microdilution assay	**Bark** water extract: activity against **HI (IZD = 22.51 ± 0.92 mm**, MIC = 1 mg/mL) and **SA (IZD = 21.16 ± 0.16 mm**, MIC = 0.5 mg/mL), no activity against MS and SP; methanol extract: activity against SA (IZD = 22.22 ± 0.38 mm, MIC = 0.5 mg/mL), no activity against HI, MS, and SP.	South Africa	[238]
			Microdilution assay	**Leaf** petroleum ether extract: activity against **BS (MIC = 3.13 µg/mL), EC (MIC = 1.56 µg/mL), KP and SA (MIC = 6.25 µg/mL each)**; dichloromethane extract: activity against **EC (MIC = 0.10 µg/mL)**, **BS (MIC = 0.195 µg/mL)**, **KP (MIC = 0.39 µg/mL)**, and **SA (MIC = 0.20 µg/mL)**; ethanol extract: activity against **SA (MIC = 0.01 µg/mL), BS (MIC = 0.20 µg/mL), EC and KP (MIC = 1.56 µg/mL each)**; water extract: activity against **BS and SA (MIC = 0.78 µg/mL each)**, **EC and KP (MIC = 0.39 µg/mL each)**.	South Africa	[24]
			Broth microdilution method	**Bark** methanol extract: activity against SE and SA (MIC = 3.75 mg/mL each); water extracts: activity against SE and SA (MIC = 2.50 mg/mL each).	South Africa	[148]

SNo.	FAMILY	PLANT NAME	METHOD OF ANTI-MICROBIAL ACTIVITY TEST EMPLOYED	ANTIMICROBIAL ACTIVITY OF EXTRACT	COUNTRY	BOOK OR JOURNAL REFERENCE
104.	Phyllanthaceae	*Antidesma membranaceum* Müll. Arg.	Two fold microdilution method	**Stem bark** ethanol extract: activity against MMA (MIC = 1.25 mg/mL), and MIP (MIC = 0.3125 mg/mL).	Tanzania	[169]
			Two fold microdilution method	**Root** ethanol extract: activity against MMA (MIC = 0.62 mg/mL) and MIP (MIC = 1.25 mg/mL).	Tanzania	[78]
105.		*Antidesma venosum* E. Mey. ex.Tul.	Microplate alamar blue assay (MABA)	**Root bark** ethanol extract: activity against PBA (MIC = 0.25 mg/mL), no activity against MS (MIC > 1 mg/mL).<br>**Leaf** ethanol extract: no activity against MS (MIC > 1 mg/mL) and PBA (MIC > 0.5 mg/mL).	South Africa	[160]
			In vitro broth microdilution screening assay	**Leaf** *n*-hexane extract: activity against MS (MIC = 1.25 mg/mL); dichloromethane extract: activity against MS (MIC = 1.25 mg/mL); acetone extract: activity against MS (MIC = 0.260 mg/mL); methanol extract: activity against MS (MIC = 0.90 mg/mL).	South Africa	[126]
106.		***Bridelia micrantha* (Hochst.) Baill.**	Micro-titer plate dilution technique	**Bark** dichloromethane:methanol (1:1) extract: activity against SA (MIC = 2 mg/mL), SA MR (MIC = 2 mg/mL), SA GMR (MIC = 2 mg/mL), SE (MIC = 2 mg/mL), and PA (MIC = 2 mg/mL); water extract: activity against SA (MIC = 8 mg/mL), SA MR (MIC = 6 mg/mL), SA GMR (MIC = 4 mg/mL), SE (MIC = 8 mg/mL), no activity against PA ((MIC > 16 mg/mL).<br>**Leaf** dichloromethane:methanol (1:1) extract: activity against SA (MIC = 2 mg/mL), SA MR (MIC = 1 mg/mL), SA GMR (MIC = 1 mg/mL), SE (MIC = 2 mg/mL), and PA (MIC = 2 mg/mL); water extract: activity against SA (MIC = 8 mg/mL), SA MR (MIC = 4 mg/mL), SA GMR (MIC = 16 mg/mL), SE (MIC = 16 mg/mL), no activity against PA ((MIC > 16 mg/mL).	southern Africa	[32]
			Disc diffusion assay (6 mg	**Stem bark** methanol extract: activity against EC 25922 (IZD = 15 mm, MIC = 1.25 mg/mL), EC 35922, EA 29751 and EA 13048 (IZD = 14 mm each, MIC	Cameroon	[186]

SNo.	FAMILY	PLANT NAME	METHOD OF ANTI-MICROBIAL ACTIVITY TEST EMPLOYED	ANTIMICROBIAL ACTIVITY OF EXTRACT	COUNTRY	BOOK OR JOURNAL REFERENCE
			extract/disc), agar dilution assay	= 10 mg/mL each), EBC HGY18 (IZD = 16 mm, MIC = 2.5 mg/mL), KP HGY19 (IZD = 15 mm, MIC = 10 mg/mL), KP HGY6 and KO U103 (IZD = 16 mm each, MIC = 10 mg/mL each), SRM HGY4 and SMR HYG10 (IZD = 16 mm each, MIC = 5 mg/mL each), PA 27853 (IZD = 16 mm, MIC = 1.25 mg/mL), AB HGY13 (IZD = 15 mm, MIC = 1.25 mg/mL), AB HGY12 (IZD = 17 mm, MIC = 2.5 mg/mL), **EH 9790 (IZD = 27 mm**, MIC = 5 mg/mL), *Enterococcus* sp. P054 (IZD = 15 mm, MIC = 1.25 mg/mL each), SA 25923 25922 (IZD = 16 mm, MIC = 1.25 mg/mL), **SA U127 (IZD = 25 mm,** MIC = 1.25 mg/mL), and SSP (IZD = 17 mm, MIC = 2.5 mg/mL).		
			Resazurin microplate assay (REMA)	**Bark** acetone extract: activity against **MTB (H37Ra) (MIC = 25 ± 0 µg/mL)** and **MTB 2 (MIC = 25 ± 1 µg/mL)**.	South Africa	[28]
			Broth microdilution method	**Bark** methanol extract: activity against SE and SA (MIC = 4 mg/mL each); water extract: activity against SE (MIC = 1.25 mg/mL), and SA (MIC = 5 mg/mL).	South Africa	[148]
107.		*Flueggea virosa* (Roxb. ex Willd.) Royle	Broth microdilution method	**Leaf** acetone extract: activity against MS (MIC = 0.625 mg/mL), MF (MIC = 2.5 mg/mL), MA (MIC = 1.25 mg/mL) and MTB (TB8104) (MIC = 0.312 mg/mL)	South Africa	[145]
			Well diffusion antimicrobial activity (5 µg extract/well)	**Leaf/stem** methanol extract: activity against SA (IZD = 0.5 cm for ATC29213, and ZD = 1.0 cm for NCRL), and BS (IZD = 1.0 cm); medium activity against SE, poor activity against EC.	Uganda	[183]
		**Securinega virosa (Roxb. ex Willd.) Baill.)** (syn.	Disc diffusion method (0.5 mg extract /disc)	**Fruit pulp** extract: ether fraction active against **SA (IZD = 15-20 mm)**; ether and petrol fractions not active against SA.	Tanzania	[118]

SNo.	FAMILY	PLANT NAME	METHOD OF ANTI-MICROBIAL ACTIVITY TEST EMPLOYED	ANTIMICROBIAL ACTIVITY OF EXTRACT	COUNTRY	BOOK OR JOURNAL REFERENCE
		Flueggea virosa (Roxb. ex Willd.) Royle				
108.		***Phyllanthus amarus* Schumach. & Thonn.** (see also Patel et al., 2011 for more information)	Disc-diffusion (1 mg extract / disc) and microdilution assays	**Root** petroleum ether extract: activity against KP (IZD = 0.14 ± 0.00 mm, MIC > 12.50 mg/mL), and SA (IZD = 0.32 ± 0.00 mm, MIC = 6.25 mg/mL), no activity against EC, ML, and BS; dichloromethane extract: activity against EC (bacteriostatic effect, MIC = 1.56 mg/mL), and SA (IZD = 0.57 ± 0.03 mm, MIC = 1.560 mg/mL), no activity against ML, KP, and BS; ethanol (80%) extract: activity against EC (IZD = 0.83 ± 0.02 mm, MIC = 0.78 mg/mL), ML (IZD = 0.61 ± 0.00 mm, MIC = 0.195 mg/mL), SA (IZD 0.05 ± 0.20 mm, MIC = 1.95 mg/mL), and BS (IZD < 0.10 mm, MIC = 0.78 mg/mL), no activity against KP.<br>**Leaf** petroleum ether and dichloromethane extracts: no activity against EC, ML, KP, SA, and BS; ethanol (80%) extract: activity against EC (bacteriostatic effect, MIC = 0.195 mg/mL), KP (bacteriostatic effect, MIC = 0.780 mg/mL), SA (IZD = 0.21 ± 0.12 mm, MIC = 0.390 mg/mL), and BS (IZD < 0.10 mm, MIC = 1.56 mg/mL), no activity against ML.	Nigeria	[187]
			Disc diffusion method (10 mg extract / disc), microdilution method	**Leaf** water extract (10 mg): activity against **PA** (IZD = 14.35 mm, **MIC = 30 μg/mL)**, **PA NCTC10662** (IZD = 10.85 mm, **MIC = 30 μg/mL), SA** (IZD = 18.20 mm, **MIC = 20 μg/mL), SA NCTC6571** (IZD = 13.39 mm, **MIC 20 = μg/mL), SP** (IZD = 16.8 mm, **MIC = 20 μg/mL)**, and SPN (IZD = 17.6 mm); ethanol extract (10 mg): activity against **PA** (IZD = 13.86 mm, **MIC = 30 μg/mL), PA NCTC10662** (IZD = 10.94 mm, **MIC = 35 μg/mL), SA** (IZD = 17.82 mm, **MIC = 20 μg/mL), SA NCTC6571** (IZD = 13.84 mm, **MIC = 20 μg/mL), SP** (IZD = 16.58 mm, **MIC = 20 μg/mL)**, and **SPN** (IZD = 16.79 mm, **MIC = 20 μg/mL)**.	Nigeria	[33]
			MIC	**Leaf** water extract: activity against MTB H37RV (MIC = 64 mg/mL), MTB RF94 (MIC = 16 mg/mL), and MU 02003 (MIC = 64 mg/mL).	Côte d'Ivoire	[239]

SNo.	FAMILY	PLANT NAME	METHOD OF ANTI-MICROBIAL ACTIVITY TEST EMPLOYED	ANTIMICROBIAL ACTIVITY OF EXTRACT	COUNTRY	BOOK OR JOURNAL REFERENCE
			Well diffusion technique (well diameter: 6 mm)	**Leaf/bark** water extract (60 mg/mL): activity against ST (IZD = 11 mm), SA (IZD = 10 mm), KP (IZD = 9 mm), and EC (IZD = 7 mm); ethanol extract (60 mg/mL): activity against ST (IZD = 12 mm), SA and KP (IZD = 10 each), and EC (IZD = 11 mm); ethyl acetate extract (60 mg/mL): activity against ST (IZD = 7 mm), SA and KP (IZD = 10 mm), and EC (IZD = 8 mm); *n*-hexane extract (60 mg/mL): activity against ST (IZD = 6 mm), SA (IZD = 8 mm), KP (IZD = 5 mm), and EC (IZD = 10 mm).	Nigeria	[240]
109.		***Pseudolachnostylis maprouneifolia* Pax**	Dilution method	**Stem bark** water extract: activity against EC (MIC = 62.50 μg/mL), **SHF (MIC = 15.62 μg/mL)**, **SD and STM (MIC = 31.25 μg/mL each)**, SEN (MIC = 62.50 μg/mL), no activity against SA (MIC > 500 μg/mL).	Democratic Republic of Congo	[241]
110.	Polygalaceae	***Securidaca longipedunculata* Fresen.**	Cylinder plate technique (100 μg extract / well)	**Root bark** water extract: activity against SA and EC (MIC = 1000 mg/mL each, ZD = 12 mm each), and PA (MIC = 1000 mg/mL, IZD = 13 mm): methanol extract: activity against EC (MIC = 1000 mg/mL, IZD = 8 mm), no activity against PA and SA.	Uganda	[117]
			Resazurin microplate assay (REMA)	**Root** acetone extract: no activity against MTB (H37Ra) (MIC > 100 ± 1.2 μg/mL) and MTB 2 (MIC > 100 ± 1.5 μg/mL).	South Africa	[26]
			Broth microdilution method	**Root** *n*-hexane extract: activity against MS ATCC 607 (MIC = 500 μg/mL), MTB H37Rv (MIC = 125 μg/mL), MTB H37Ra and MS mc^2 (MIC = 62.5 μg/mL), MB BCG (MIC = 31.2 μg/mL), MBA DSM 44156 (MIC = 250 μg/mL), no activity against MBA DSM 44157 (MIC > 250 μg/mL).	Mozambique	[156]
			Microplate serial dilution method (500 μg extract / disc)	**Root** ethanol extract: no activity against MA.<br>Root bark ethanol extract: activity against PA (IZD = 1 ± 0 mm), no activity against BS, BC, EC, and SA.	Botswana	[204]

SNo.	FAMILY	PLANT NAME	METHOD OF ANTI-MICROBIAL ACTIVITY TEST EMPLOYED	ANTIMICROBIAL ACTIVITY OF EXTRACT	COUNTRY	BOOK OR JOURNAL REFERENCE
			Disc diffusion method (10 mg extract / disc)	**Root** petroleum ether, dichloromethane, and ethanol extracts: no activity against SA, EC, and PA.	Tanzania	[242]
			Agar diffusion test (disc diameter: 6 mm)	**Root bark** *n*-hexane extract (200 mg/mL): activity against BS (IZD = 14 mm), EC (IZD = 12 mm), and PA (IZD = 10 mm), no activity against SA; chloroform extract: activity against **BS (IZD = 20 mm)**, SA (IZD = 12 mm), **EC (IZD = 30 mm)**, and PA (IZD = 11 mm); acetone extract: activity against BS (IZD = 32 mm), **SA (IZD = 26 mm)**, **EC (IZD = 20 mm)**, and **PA (IZD = 32 mm)**; methanol extract: activity against **BS (IZD = 30 mm)**, SA (IZD = 26 mm), **EC (IZD = 34 mm)**, and **PA (IZD = 28 mm)**.	Ethiopia	[243]
			Well-diffusion assay (well diameter: 4 mm)	**Root** methanol extract (10 mg/mL): activity against SA (IZD = 1.50 ± 0.41 mm), *Streptococcus* group A (IZD = 3.50 ± 0.58 mm), no activity against EC and PA.	Zimbabwe	[128]
			Disc diffusion assay (200 µg extract / disc) + Broth microdilution assay	**Root** aqueous extract: activity against **SP (IZD = 20.84 ± 0.22 mm**, MIC = 0.25-1 mg/mL as per strain), no activity against HI, MS, and SA.	South Africa	[140]
			Disc diffusion method (disc diaemeter: 6 mm)	**Stem** hot water extract (10 g/L): activity against EC (IZD = 7.2 ± 0.6 mm), SA (IZD = 12.5 ± 2.2 mm), and BS (IZD = 12.5 ± 0.4 mm).	Kenya	[116]
			Broth microdilution method	**Not specified** methanol extract: no activity against MTB (MIC > 2500 µg/mL), weak activity against MB (MIC = 2125 µg/mL).	Nigeria	[197]

SNo.	FAMILY	PLANT NAME	METHOD OF ANTI-MICROBIAL ACTIVITY TEST EMPLOYED	ANTIMICROBIAL ACTIVITY OF EXTRACT	COUNTRY	BOOK OR JOURNAL REFERENCE
111.	Polygonaceae	*Rumex usambarensis* (Dammer) Dammer	Agar dilution streak method	**Leaf** methanol extract: activity against BS, MS and SA, inactive against PA and SG at concentrations of 1000 µg/mL.<br>**Stem** and **root** methanol extracts: no activity against BS, MS, PA, SG and SA at concentrations of 1000 µg/mL	Rwanda	[96]
			Proportion method	**Leaf** ethanol (95%) extracts: no activity against MTB.	Rwanda	[192]
			Disc diffusion method	**Root** methanol extract: no activity against NG, NM, SP and SA at concentrations of 1000 µg/mL.	Rwanda	[206]
112.	Polypodiaceae	*Phymatosorus scolopendria (Burm. f.) Pic. Serm. (syn Phymatodes scolopendria (Burm. f.) Ching.)*	Microbial percent inhibition assay (50 µg extract / well)	**Leaf** methanol extract: activity against SA (IZD = 7 mm), no activity against EC; hexane: activity against SA (IZD = 2 mm), no activity against EC.	Tonga	[102]
113.	Ranunculaceae	*Clematis brachiata* Thunb.	Microplate alamar blue assay (MABA)	**Root/leaf/stem** ethanol extract: no activity against MS (MIC > 1 mg/mL), and PBA (MIC > 0.5 mg/mL)	South Africa	[160]
			MIC assay	**Leaf and stem** dichloromethane:methanol extract: activity against KP and MS (MIC = 2.00 mg/mL each), MC (MIC = 3.33 mg/mL), and SA (MIC = 6.67 mg/mL); water extract: activity against KP and MS (MIC = 16.00 mg/mL each), SA (MIC = 8 mg/mL), no activity against MC.	South Africa	[230]
			Agar dilution assay	**Leaf** acetone extract: activity against SA, SE, BC, MCK, SF, EC, PA, SHF, KP, and SM (MIC = 2.0 mg/mL each); methanol extract: activity against SA, SE, BC, MCK, SF, EC, KP, and SM (MIC = 2.0 mg/mL each), PA (MIC = 3 mg/mL), and SHF (MIC = 1 mg/mL); water extract: activity against PA and SHF (MIC = 10 mg/mL), no activity against SA, SE, BC, MCK, SF, EC, KP, and SM.	South Africa	[244]

SNo.	FAMILY	PLANT NAME	METHOD OF ANTI-MICROBIAL ACTIVITY TEST EMPLOYED	ANTIMICROBIAL ACTIVITY OF EXTRACT	COUNTRY	BOOK OR JOURNAL REFERENCE
			Micro-titre plate dilution assay	**Leaf** dichloromethane:methanol extract: activity against SM and PG (MIC = 2.00 mg/mL each), SS (MIC = 0.67 mg/mL), and FN (MIC = 1.33 mg/mL), no activity against LA and LC; water extract: activity against SM (MIC = 2 mg/mL), SS (MIC = 6 mg/mL), PG (MIC = 4 mg/mL), no activity against LA, LC and FN.<br>**Stem** dichloromethane:methanol extract: activity against SM and PG (MIC = 2.00 mg/mL each), SS (MIC = 1.67 mg/mL), LA (MIC = 3.33 mg/mL), LC (MIC = 8 mg/mL), and FN (MIC = 2.67 mg/mL), no activity against SS; water extract: activity against SM (MIC = 4 mg/mL), LA, PG, and FN (MIC = 8 mg/mL), no activity against SS and LC.<br>**Flowers** dichloromethane:methanol extract: activity against SM and FN (MIC = 2.00 mg/mL each), SS (MIC = 0.83 mg/mL), LC and PG (MIC = 8 mg/mL each), no activity against LA; water extract: activity against SM and SS (MIC = 4 mg/mL each), FN (MIC = 8 mg/mL), no activity against LA, LC, and PG.	South Africa	[143]
114.		*Clematis hirsuta* Guill. & Perr. var. *hirsuta*	Agar dilution streak method	**Leaf** and **stem** methanol extracts: no activity against BS, MS, PA, SG and SA at concentrations of 1000 µg/mL	Rwanda	[96]
115.	Rhamnaceae	*Rhamnus prinoides* L'Hér.	Broth microdilution method	**Leaf** acetone extract: activity against MS and MTB (TB8104) (MIC = 0.625 mg/mL each), MF (MIC = 2.5 mg/mL), no activity against MA (MIC > 2.5 mg/mL)	South Africa	[145]
			Resazurin based microtitre dilution technique	**Leaf** methanol (80%) extract: activity against SA (MIC = 2.03 mg/mL), SP (MIC = 4.06 mg/mL), SPN (MIC = 8.13 mg/mL), SHF (MIC = 4.06 mg/mL for standard strain and 5.42 ± 1.36 mg/mL for clinically isolated strain), EC (MIC = 8.13 mg/mL), and ST (MIC = 4.06 mg/mL); methanol fraction: activity against SA and SP (MIC = 8.13 mg/mL each), SPN, SF, PA, and ST (MIC = 16.25 mg/mL each), and EC (MIC = 32.50 mg/mL); chloroform fraction: activity against SA and SP (MIC = 8.13 mg/mL for standard strain	Ethiopia	[245]

SNo.	FAMILY	PLANT NAME	METHOD OF ANTI-MICROBIAL ACTIVITY TEST EMPLOYED	ANTIMICROBIAL ACTIVITY OF EXTRACT	COUNTRY	BOOK OR JOURNAL REFERENCE
				and 16.2 mg/mL for clinically isolated strain each), SPN, SHF, and ST (MIC = 16.25 mg/mL), no activity against EC and PA.		
116.		***Scutia myrtina*** **(Burm. f.) Kurz**	Agar disc diffusion method	**Whole plant** extract: no activity against NG and HD.	India	[246]
			Agar diffusion method and two folds serial dilution method	**Entire plant** petroleum ether extract (IZD at conc. 1000 μg/mL): activity against **PA (IZD = 24 mm,** MIC = 125 μg/mL), **EC (IZD = 20 mm**, MIC = 125 μg/mL) **ST (IZD = 24 mm**, MIC = 500 μg/mL), SA (IZD = 18 mm, MIC = 500 μg/mL), SP (IZD = 19 mm), **BS (IZD = 20 mm)**; methanol extract (IZD at con. 1000 μg/mL): activity against: PA (IZD = 15 mm, MIC = 250 μg/mL), EC (IZD = 11 mm, MIC = 250 μg/mL) ST (IZD = 16 mm, MIC = 500 μg/mL), SA (IZD = 10 mm, MIC = 500 μg/mL), SP (IZD = 11 mm), BS (IZD = 11 mm);	India	[247]
117.		*Ziziphus abyssinica Hochst. ex A. Rich.*	n.d.	**Fresh fruit** methanol extract: activity against SA (IZD = 15.6 ± 0.7 mm for 200 mg/mL and 9.0 ± 0.8 mm for 100 mg/mL), PA (IZD = 11.3 ± 0.3 mm for 200 mg/mL and < 9 mm for 100 mg/mL), and EC (IZD = 12.7 ± 0.3 mm for 200 mg/mL and 10.7 ± 0.5 mm for 100 mg/mL, MIC = 25 mg/mL); water extract: active against SA (IZD = 14.7 ± 0.3 mm for 200 mg/mL and < 9 mm for 100 mg/mL, MIC = 3.1 mg/mL), PA (IZD < 9 mm, MIC = 50 mg/mL), and EC (IZD = 13.0 ± 0.6 mm for 200 mg/mL and 9.7 ± 0.5 for 100 mg/mL, MIC = 25 mg/mL).	Kenya	[248]
			Broth dilution method	**Root/bark** methanol (70%) extract: activity against BC (MIC = 28.75 mg/mL), no activity against ML, PA and EC.	Kenya	[164]
118.		***Ziziphus pubescens*** **Oliv**	Disc diffusion method (0.5 mg extract /disc)	**Leaf** extract: petrol and ether fractions active against SA (IZD = 10-15 mm each); chloroform fraction active against **SA (IZD = 20-25 mm**).	Tanzania	[118]

SNo.	FAMILY	PLANT NAME	METHOD OF ANTI-MICROBIAL ACTIVITY TEST EMPLOYED	ANTIMICROBIAL ACTIVITY OF EXTRACT	COUNTRY	BOOK OR JOURNAL REFERENCE
119.	Rubiaceae	*Crossopteryx febrifuga* (Afzel. ex G. Don) Benth.	Liquid dilution method	**Stem bark** *n*-hexane extract: activity against BC and SA (MIC = 0.5 mg/mL each), STM (MIC = 1 mg/mL), no activity against KP, PA and EC.	Tanzania	[109]
			Dilution assay	**Stem bark** methanol extract: active against EA, EC, and SA (MIC = 1024 μg/mL), and KP (MIC= 512 μg/mL).	Cameroon	[109]
120.		*Pavetta crassipes* K. Schum.	Disc-plate method (750 μg extract / test)	**Leaf** ethanol extract (1-8 mg/mL): activity against SA (IZD = 8-13 mm) and PA (IZD = 8 mm at all conc.); no activity against KP (IZD = 8 mm at 1 mg/mL, otherwise 0 mm) and *Proteus* spp. (IZD = 7 mm at 1 mg/mL, otherwise 0 mm); chloroform extract (1-8 mg/mL): activity against SA (IZD = 7 mm at 5 and 8 mg/mL, otherwise 0 mm), *Proteus* spp. (IZD = 7-19 mm), no activity against KP and PA.	Nigeria	[249]
			Micro plate agar dilution method	**Leaf** *n*-hexane extract: no activity against MA (MIC > 1000 μg/mL); dichloromethane extract: no activity against MA (MIC > 1000 μg/mL); methanol extract: activity against MA (MIC = 250 μg/mL); water extract: no activity against MA (MIC > 1000 μg/mL).	Nigeria	[250]
			Well diffusion technique (well diameter: 6 mm)	**Leaf/bark** water extract (60 mg/mL): activity against ST, SA, and KP (IZD = 10 mm each), and EC (IZD = 7 mm); ethanol extract (60 mg/mL): activity against ST and SA (IZD = 10 each), KP (IZD 0 7 mm), and EC (IZD = 9 mm); ethyl acetate extract (60 mg/mL): activity against ST, SA, and KP (IZD = 7 mm), and EC (IZD = 8 mm); n-hexane extract (60 mg/mL): activity against ST (IZD = 8 mm), SA and KP (IZD = 9 mm each), and EC (IZD = 10 mm).	Nigeria	[240]
121.		*Rubia cordifolia* L.	Agar dilution streak method	**Whole plant** methanol extract: no activity against BS, MS, PA, SG and SA at concentrations of 1000 μg/mL	Rwanda	[96]
			Disc diffusion method	**Whole plant** methanol extract: weak activity against NG, NM, SP and SA at concentrations of 1000 μg/mL.	Rwanda	[206]

SNo.	FAMILY	PLANT NAME	METHOD OF ANTI-MICROBIAL ACTIVITY TEST EMPLOYED	ANTIMICROBIAL ACTIVITY OF EXTRACT	COUNTRY	BOOK OR JOURNAL REFERENCE
			Two fold microdilution method	**Stem bark** ethanol extract: activity against MMA (MIC = 2.5 mg/mL), and MIP (MIC = 1.25 mg/mL).	Tanzania	[169]
			Agar-disc diffusion method (20 μg extract / disc)	**Root** methanol (70%) extract: activity against SA (IZD = 6 ± 0.5 mm), BS (IZD = 16 ± 0.6 mm), SE (IZD = 7 ± 0.3 mm), SF (IZD = 14 ± 0.6 mm), BC (IZD = 14 ± 0.3 mm), VP (IZD = 10 ± 0.6 mm), PA (IZD = 7 ± 0.5 mm), STM (IZD = 6 ± 0.4 mm), and EC (IZD = 13 ± 0.4 mm).	China	[41]
122.	Rutaceae	***Citrus limon* (L.) Osbeck**	Micro-titre plate dilution assay	**Essential oil** micelle solution: activity against SA MSSA ATCC 29213, SA MRSA ATCC 43300, and EC (MIC = 6.3% each), EF, KP, and PA (MIC = 12.5% each); aqueous phase: activity against SA MSSA ATCC 29213 and KP (MIC = 25% each), SA MRSA ATCC 43300, EF, and EC (MIC = 50% each), no activity against PA (MIC > 50%).	n.d.	[251]
			Disc diffusion method (0.1 mL essential oil / disc)	**Unripe lemon peel oil**: activity against BS (IZD = 8.60 ± 0.52 mm), EC (IZD = 15.14 ± 0.24 mm), SA (IZD = 15.33 ± 0.70 mm), and SPM (IZD = 14.83 ± 0.09 mm).<br>**Ripened lemon peel oil**: activity against BS (IZD = 13.63 ± 0.55 mm), EC (IZD = 19.07 ± 0.12 mm), SA (IZD = 17.00 ± 0.53 mm), and SPM (IZD = 15.85 ± 0.49 mm).	Pakistan	[252]
			E-test method	**Leaf essential oil**: activity against SA and BS (MIC = 200 μg/mL each), and EC (MIC = 400 μg/mL), no activity against ST.	Iran	[253]
			Agar diffusion (hole diameter: 8 mm) and serial dilution methods	**Essential oil** (IZD at 100 μg/mL): activity against **SA (IZD = 22.1 ± 2.46 mm, MIC = 12.5 μg/mL), BS (IZD = 23.5 ± 2.32 mm, MIC = 6.25 μg/mL), PA** (IZD = 19.5 ± 1.62 mm, **MIC = 12.5 μg/mL),** and **EC** (IZD = 8.2 ± 1.30 mm, **MIC = 50 μg/mL)**.	India	[38]

SNo.	FAMILY	PLANT NAME	METHOD OF ANTI-MICROBIAL ACTIVITY TEST EMPLOYED	ANTIMICROBIAL ACTIVITY OF EXTRACT	COUNTRY	BOOK OR JOURNAL REFERENCE
			MIC assay	**Leaf** dichloromethane:methanol extract: activity against KP and MC (MIC = 2.67 mg/mL each), MS (MIC = 2.00 mg/mL), and SA (MIC = 5.33 mg/mL); water extract: no activity against KP, MC, MS, and SA (MIC > 16.00 mg/mL each).	South Africa	[230]
			Disc diffusion method	**Peel oil**: activity against EC and SA (IZD = 14 mm each), KP, BC, and MS (IZD = 13 mm each), ML (IZD = 12 mm), PV and LM (IZD = 16 mm=, and PA (IZD = 10 mm).		[254]
123.		***Fagara chalybaea* Engl. (Engl.)**	Disc diffusion method (0.5 mg extract /disc)	**Root bark** extract: petrol fraction active against SA (IZD = 10-15 mm); ether fraction active against **SA (IZD = 20-25 mm)**; chloroform fraction active against **SA (IZD = 10-20 mm)**.	Tanzania	[118]
124.		*Gardenia ternifolia* subsp. *jovis-tonantis* (Welw.) Verdc.	Micro-dilution method	**Leaf** ethanol (95%) and aqueous extract (obtained via repeated extraction by cold percolation): anthocyanins extract active against EC (MIC = 125 μg/mL), and SA (MIC = 62.5 μg/mL); organic acids extract active against EC (MIC = 125 μg/mL), and SA (MIC = 125 μg/mL).	Democratic Republic of the Congo	[255]
125.		***Harrisonia abyssinica* Oliv**	Liquid dilution method	**Root** *n*-hexane extract: activity against BC (MIC = 0.0625 mg/mL) and SA (MIC = 0.125 mg/mL). Root methanol extract: activity against BC (MIC = 0.125 mg/mL) and SA (MIC = 0.0625 mg/mL).	Tanzania	[109]
			Agar diffusion antimicrobial assay (10 μg extract / disc)	**Ripe berry** methanol-dichloromethane (1:1) extract: activity against **BC (IZD = 35 ± 1 mm)**, EC (IZD = 30 ± 2 mm), STM (IZD = 10 ± 1 mm), **SA (IZD = 20 ± 2 mm)**, and LC (IZD = 17 ± 1 mm).	Kenya	[256]

SNo.	FAMILY	PLANT NAME	METHOD OF ANTI-MICROBIAL ACTIVITY TEST EMPLOYED	ANTIMICROBIAL ACTIVITY OF EXTRACT	COUNTRY	BOOK OR JOURNAL REFERENCE
			Broth dilution method	**Leaf/bark** methanol (70%) extract: activity against ML (MIC = 25 mg/mL), BC (MIC = 15.6 mg/mL), PA (MIC = 37.5 mg/mL each), and EC (MIC = 150 mg/mL).	Kenya	[164]
126.		***Toddalia asiatica* (L.) Lam.**	Disc diffusion method (10 μL essential oil / disc) and turbidimetric or tube dilution method	**Essential oil**: activity against **BC (IZD = 25.33 ± 0.88 mm**, MIC = 3.13% essential oil), **SA (IZD = 21.67 ± 1.45 mm**, MIC = 25.00% essential oil), **EC (IZD = 34.00 ± 2.65 mm**, MIC = 12.25% essential oil), and **MR SA (IZD = 20.00 ± 1.15 mm**, MIC = 6.25% essential oil).	Kenya	[257]
			Agar disc diffusion method (impregnation with 300 mg extract/mL), BACTEC MGIT™ 960 system	**Plant** methanol extract: activity against MK, MTB, MF, and MS (complete inhibition at a conc. of 2 mg/mL), ST (IZD = 6.33 mm), SA (IZD = 8.66 mm), EC (IZD = 7 mm), PA (IZD = 10.66 mm), and KP (IZD = 6.00 mm).	Kenya	[196]
			Dics diffusion (250 μg extract / disc) and tube doubling dilution methods	**Root** petroleum ether fraction: activity against SA (IZD = 8.13 ± 0.18 mm, MIC = 50 mg/mL), SA MR (IZD = 9.21 ± 0.18 mm, MIC = 25 mg/mL), no activity against SA ESBLs; ethyl acetate fraction: activity against SA (IZD = 8.06 ± 0.13 mm, MIC = 50 mg/mL), SA MR (IZD = 8.00 ± 0.18 mm, MIC = 50 mg/mL), no activity against SA ESBLs; methanol fraction: activity against SA (IZD = 7.95 ± 0.28 mm, MIC = 50 mg/mL), no activity against SA MR and SA ESBLs.	China	[258]

SNo.	FAMILY	PLANT NAME	METHOD OF ANTI-MICROBIAL ACTIVITY TEST EMPLOYED	ANTIMICROBIAL ACTIVITY OF EXTRACT	COUNTRY	BOOK OR JOURNAL REFERENCE
			Disc diffusion test (250 μg extract / disc)	**Root** petroleum ether extract: activity against SA (IZD = 8 mm), MR SA (IZD = 9 mm); ethyl acetate extract: activity against SA (IZD = 8 mm), low or no activity against MR SA; DMSO extract: low or no activity against SA and MR SA. All extracts showed no activity against extended-spectrum β-lactamase positive SA.	China	[259]
			Agar disc diffusion assay	**Root** water extract (50 μg/disc): activity against EC, SP, and SAL (IZD = 16 mm each), SHF and SA (IZD = 17 mm), XM, PV, CV, and SP (IZD = 15 mm each); water extract (100 μg/disc): activity against EC, ST, SHF, CV, SA, and SAL (IZD = 18 mm each), XM, PV, and SP (IZD = 17 mm each).	India	[260]
			Disc diffusion method (1.25 – 5 mg extract / disc)	**Leaf** *n*-hexane extract: activity against **SA (IZD = 17-30 mm)** and **SE (IZD = 13-20 mm)**, no activity against BS, EF, EC, PA, KP, EAM, and PV; chloroform extract: activity against SA (IZD = 12-18 mm) and SE (IZD = 11-19 mm), no activity against BS, EF, EC, PA, KP, EAM, and PV; ethyl acetate extract: activity against **SA (IZD = 18-32 mm)**, **SE (IZD = 12-23 mm)**, BS (IZD = 11-16 mm), EF (IZD = no activity -12 mm), PA (IZD = 8-12 mm), no activity against EC, KP, EAM, and PV; methanol extract: activity against SA (IZD = no activity-15 mm), SE (IZD = no activity-16 mm), BS (IZD = no activity– 0 mm), EF (IZD = no activity–9 mm), no activity against EC, PA, KP, EAM, and PV; water extract: activity against SA (IZD = no activity-10 mm), SE (IZD = no activity–12 mm), no activity against BS, EF, EC, PA, KP, EAM, and PV.	India	[261]
127.		***Zanthoxylum chalybeum* Engl.**	Broth microdilution method	**Stem bark** ethanol (80%) extract: no activity against EF, SA, EC, and PA.	Uganda	[147]

SNo.	FAMILY	PLANT NAME	METHOD OF ANTI-MICROBIAL ACTIVITY TEST EMPLOYED	ANTIMICROBIAL ACTIVITY OF EXTRACT	COUNTRY	BOOK OR JOURNAL REFERENCE
			Two fold microdilution method	**Stem bark** dichloromethane extract: activity against MMA (MIC = 1.25 mg/mL) and MIP (MIC = 2.5 mg/mL); methanol extract: activity against MMA (MIC = 1.25 mg/mL) and MIP (MIC = 2.5 mg/mL).	Tanzania	[262]
			Agar well diffusion method (12.5 mg extract/well)	**Root** hot water extract: activity against **BC (IZD = 21.67 ± 1.20 mm)**, no activity against SA and EC (IZD < 8.00 mm).	Kenya	[191]
			Paper disc assay, agar assay	**Stem** water, petroleum ether and ethanol extracts: no activity against SA and EC in both assays.<br>**Seed** water, petroleum ether and ethanol extracts: no activity against SA and EC in both assays.	Uganda	[150]
128.	Smilacaceae	*Smilax anceps* Willd.	Agar dilution method	**Aerial part** methanol extract: activity against SA (MIC = 2 mg/mL), EC; PA, and ST (MIC = 8 mg/mL).	Mauritius	[263]
129.	Solanaceae	*Solanum incanum* L.	Agar dilution streak method	**Leaf** and **stem** methanol extracts: no activity against BS, MS, PA, SG and SA at concentrations of 1000 μg/mL	Rwanda	[96]
			Disc diffusion method	**Fruit** methanol extract: no activity against NG, NM, SP and SA at concentrations of 1000 μg/mL.	Rwanda	[206]
			Micro-titer plate dilution technique	**Leaf** dichloromethane:methanol (1:1) extract: activity against SA, SE, and PA (MIC = 0.5 mg/mL each), SA MR and SA GMR (MIC = 1.0 mg/mL each); water extract: no activity against SA, SA MR, SA GMR, and PA (MIC > 16 mg/mL each).	southern Africa	[32]
			Broth microdilution method	**Fruit** ethanol extract: no activity against EF, SA, EC, and PA.	Ethiopia	[124]

SNo.	FAMILY	PLANT NAME	METHOD OF ANTI-MICROBIAL ACTIVITY TEST EMPLOYED	ANTIMICROBIAL ACTIVITY OF EXTRACT	COUNTRY	BOOK OR JOURNAL REFERENCE
			Disc diffusion method (0.5 mg extract /disc)	**Whole plant** extract: petrol, ether and chloroform fractions not active against SA.	Tanzania	[118]
			Agar diffusion technique	**Fruit** dichloromethane extract: activity against BC (MIC = 1000 µg/mL), no activity against SA, MCF, EC, and PA; methanol extract: activity against BC (MIC = 1000 µg/mL), and MCF (MIC = 500 µg/mL), no activity against SA, EC, and PA; water extract: activity against BC, EC, and PA (MIC = 1000 µg/mL each), no activity against SA and MCF.	Yemen	[36]
			Bacteria assay on agar plates	**Fruit** methanol extract: activity against BC, BP, and BS (MIC = 0.5 mg/mL each), MCK, SA, EBC and SRM (MIC = 5 mg/mL each), no activity against EA, EC, and PV; acetone extract: activity against BP, BS, MCK, SA, and EBC (MIC = 5 mg/mL each), no activity against EA, EC, PV, and SRM; water extract: no activity against BC, BP, BS, MCK, SA, EBC, EA, EC, PV, and SRM.	Zimbabwe	[202]
			Agar diffusion method	**Fruit** hot water extract: activity against SM serotype c (MT 5091) and serotype d (OMZ 176) (MIC = 62.5 mg/mL each).	Taiwan	[173]
130.	Verbenaceae	***Lantana camara* L.**<br>(see Al-Snafi, 2019 for an overview)	Agar well diffusion method	**Leaf** methanol extract: activity against **MTB (H37Rv) (MIC = 20 µg/mL)**, and **MTB (TMC-331) and wild strain (MIC= 15 µg/mL)**.	Uganda	[29]
			Microdilution method	**Leaf** ethanol extract: activity against EC ATCC25922 and EC Ec27, and PA (MIC = 256 µg/mL each), PV and VC (MIC = 128 µg/mL), and SA Sa 358 (MIC = 512 µg/mL), no activity against SA ATCC 12692 (MIC ≥ 1,024 µg/mL).<br>**Root** ethanol extract: activity against EC ATCC 52922 (MIC = 512 µg/mL), PV (MIC = 64 µg/mL), and PA (MIC = 128 µg/mL), no activity against VC, SA ATCC 12692, EC Ec 27, and SA Sa 358 (MIC ≥ 1,024 µg/mL each).	Brazil	[264]

SNo.	FAMILY	PLANT NAME	METHOD OF ANTI-MICROBIAL ACTIVITY TEST EMPLOYED	ANTIMICROBIAL ACTIVITY OF EXTRACT	COUNTRY	BOOK OR JOURNAL REFERENCE
			Two-fold serial dilution microplate method	**Leaf** acetone extract: activity against EC (MIC = 3.1 mg/mL), EF (MIC = 1.6 mg/mL), and PA (MIC = 3.1 mg/mL).	South Africa	[205]
			Agar disc diffusion method (impregnation with 300 mg extract/mL), BACTEC MGIT™ 960 system	**Plant** methanol extract: activity against MK, MTB, MF, and MS (complete inhibition at a conc. of 2 mg/mL), ST (IZD = 6.66 mm), SA (IZD = 9 mm), EC (IZD = 6 mm), PA (IZD = 8.66 mm), and KP (IZD = 7.33 mm).	Kenya	[196]
			Agar-well diffusion method	**Leaf** methanol extract: activity against SA and PA (MIC = 5 mg/mL each), BS and KP (MIC = 8 mg/mL each); ethanol extract: activity against SA (MIC = 6.5 mg/mL), BS (MIC = 10 mg/mL), PA (MIC = 8 mg/mL), and KP (MIC = 12 mg/mL); water extract: active against SA (MIC = 8 mg/mL), and PA (MIC = 10 mg/mL).	Pakistan	[265]
			Agar diffusion method	**Leaf** ethanol (80%) extract: activity against EC (MIC = 25 mg/mL), PA (MIC = 12.5 mg/mL), BS and SA (MIC = 6.25 mg/mL each).	India	[103]
131.	Vitaceae	*Cyphostemma adenocaule* (Steud. ex A. Rich.) Desc. ex Wild & R.B.Drumm	Well diffusion antimicrobial activity (5 µg extract/well)	**Leaf and stem** methanol extract: poor activity against EC, SA; non-confluent microbial lawn growth in the area of the well in case of SF**.**	Uganda	[183]
			Agar well diffusion method	**Root** water extract (50 mg/mL): activity against SA (IZD = 15 mm), no activity against BS and EC.	Somalia	[104]

SNo.	FAMILY	PLANT NAME	METHOD OF ANTI-MICROBIAL ACTIVITY TEST EMPLOYED	ANTIMICROBIAL ACTIVITY OF EXTRACT	COUNTRY	BOOK OR JOURNAL REFERENCE
			(hole diameter: 10 mm)			
132.	Xanthorrhoeaceae	*Aloe secundiflora* Engl.	BACTEC MGIT™ 960 system and Agar disc diffusion method	**Leaf** methanol extract: high antimycobacterial activity against MK, MF, and MS (0 GUs each), and activity against MTB (157 GUs at 0.5 mg/mL); activity against ST, SA, EC and PA (IZD ≥ 9.00 mm); activity against ST, SA (MIC = 37.5 mg/mL each, MBC = 37.5 mg/mL each), EC (MIC = 18.75 mg/mL, MBC = 18.75 mg/mL), and PA (MIC = 9.375 mg/mL, MBC = 18.75 mg/mL).	Kenya	[153]
			Broth dilution method	**Whole plant** methanol (70%) extract: activity against BC (MIC = 7.8 mg/mL), PA and EC (MIC = 250 mg/mL each), no activity against ML.	Kenya	[164]
			Microtitre dilution technique	**Whole plant** methanol (80%) extract: activity against SA, BS, ST, EC, and PA (MIC = 3.75 mg/50 μL each).	Kenya	[152]
133.	Zygophyllaceae	***Balanites aegyptiaca* (L.) Delile**	Agar well diffusion (stock solutions for IZD detection: 250 mg/mL) / microdilution methods	**Bark** petroleum ether: activity against EC (IZD = 16.0±1.00 mm, MIC = 150 μg/mL), **SA (IZD = 22.0±1.00 mm**, MIC = 470 μg/mL), no activity against PA (IZD = 0.0±0.00 mm); methanol extract: activity against EC, SA and PA (IZD = 0.0±0.00 mm).	Uganda	[154]
			Liquid dilution method	**Leaf** *n*-hexane extract: activity against BC (MIC = 0.25 mg/mL) and SA (MIC = 1 mg/mL); methanol extract: activity against BC (MIC = 0.5 mg/mL), and SA (MIC = 1 mg/mL).<br>**Stem bark** *n*-hexane extract: activity against BC (MIC = 1 mg/mL), no activity against SA; methanol extract: no activity against BC and SA.	Tanzania	[109]

SNo.	FAMILY	PLANT NAME	METHOD OF ANTI-MICROBIAL ACTIVITY TEST EMPLOYED	ANTIMICROBIAL ACTIVITY OF EXTRACT	COUNTRY	BOOK OR JOURNAL REFERENCE
			Broth dilution method	**Leaf** dichloromethane extract: activity against MA (MIC = 12.5 mg/mL); ethylacetate extract: no activity against MA (MIC > 25 mg/mL); ethanol extract: activity against MA (MIC = 3.12 mg/mL).<br>**Bark** dichloromethane and ethanol extract: activity against MA (MIC = 12.5 mg/mL each); ethylacetate extract: no activity against MA (MIC > 25 mg/mL).<br>**Root** dichloromethane extract: activity against MA (MIC = 25 mg/mL); ethylacetate and ethanol extract: activity against MA (MIC = 12.5 mg/mL each).	Sudan	[266]
			Microtitre dilution technique	**Root** methanol (80%) extract: activity against SA, BS, ST, and PA (MIC = 1.875 mg/50 µL each), and EC (MIC = 3.75 mg/50 µL)	Kenya	[135]
			Broth microdilution method	**Aerial parts** methanol (70%) extract: activity against SRM (MIC = 0.90 mg/mL), no activity against EC, PM, SHF, SPT, SEN, KP, and SA (MIC > 2.50 mg/mL each).	Togo	[136]

Footnote:

1. Some non-respiratory causing bacteria were included if mentioned in the cited publication

2. Acronomy for bacteria are

AA = *Actinobacillus actinomycetemcomitans*, AB = *Acinetobacter baumannii*, ABA = *Acinetobacter anitratus*, AC = *Acinetobacter cacloaceticus*, AI = *Actinomyces israelii*, AN = *Actinomyces naeslundii*, AV = *Actinomyces viscosus*, BA= *Bacillus anthracis;* BAU = *Bacillus aureus*, BB =*Bacillus brevis;* BBS = *Bordetella bronchiseptica,* BC = *Bacillus cereus;* BL=*Bacillus licheniformis*, BM = *Bacillus megaterium,* BP = *Bacillus pumilus*, BS = *Bacillus subtilis*, BT = *Bacillus thuringiensis*, BBA = *Brevibacterium agri*, BBL = *Brevibacterium linens*, CD = *Chlamydia diphtheriae*, CF = *Citrobacter freundii*, CG = *Corynebacterium glutamicum*, CP = *Chlamydia pneumoniae*, , CV = *Chromobacterium violaecum*, EA = *Enterobacter aerogenes*, EAM = *Erwinia amylovora,* EBC = *Enterobacter cloacae*, EC = *Escherichia coli*, EF = *Enterococcus faecalis*, EH = *Enterococcus hirae*, FN = *Fusobacterium nucleatum*, HD = *Haemophilus ducreyi*, HI = *Haemophilus influenzae*, KA = *Klebsiella aerogenes*, KO = *Klebsiella oxytoca*, KP = *Klebsiella pneumoniae*, KPC = *Klebsiella planticola*, LA = *Lactobacillus acidophilus*, LC = *Lactobacillus cassei*, LM = *Listeria monocytogenes*, MA = *Mycobacterium aurum*, MB = *Mycobacterium bovis*, MBA = *Mycobacterium avium*, MBC = *Mycobacterium*

chelonae, MC = *Moraxella catarrhalis*, MCF = *Micrococcus flavus*, MCK = *Micrococcus kristinae*, MF = *Mycobacterium fortuitum*, MI = *Mycobacterium intracellulare*, MIP = *Mycobacterium indicus pranii*, MK = *Mycobacterium kansasii*, ML = *Micrococcus luteus*, MM = *Morganella morganii*, MMA = *Mycobacterium madagascariense*, MP = *Mycobacterium phlei*, MS = *Mycobacterium smegmatis*, MT = *Mycobacterium terrae*, MTB = *Mycobacterium tuberculosis*, MU= *Mycobacterium ulcerans*, NG = *Neisseria gonorrheoeae*, NM = *Neisseria meningitidis*, PA = *Pseudomonas aeruginosa*, PBA = *Propionibacterium acnes*, PC = *Pseudomonas cichorii*, PF = *Pseudomonas fluorescens*, PG = *Porphrymonas gingivalis*, PI = *Prevotella intermedia*, PM = *Proteus mirabilis*, PP = *Pseudomonas putida*, PS = *Providencia stuartii*, PV = *Proteus vulgaris*, SA = *Staphylococcus aureus*, SA GMR = Gentamycin-methicillin resistant *Staphylococcus aureus*, SA MR = Methicillin-resistant *Staphylococcus aureus*, SAB = *Salmonella abony*, SAE = *Salmonella enterica*, SAG = *Streptococus agalactiae*, SAL = *Staphylococcus albus*, SAU = *Staphylococcus auricularis*, SD = *Shigella dysenterae*, SE = *Staphylococcus epidermidis*, SE MR = Methicillin resistant *Staphylococcus epidermidis*, SEN = *Salmonella enteriditis*, SF = *Streptococcus faecalis*, SG = *Salmonella gallinarum*, SH = *Staphylococcus haemolyticus*, SHB = *Shigella boydii*, SHF = *Shigella flexneri*, SHS = *Shigella sonnei*, SL = *Streptococcus lactis*, SM = *Streptococcus mutans*, SP = *Streptococcus pyogenes*, SPN = *Streptococcus pneumoniae*, SPT = *Salmonella paratyphi*, SRM = *Serratia marcescens*, SS = *Streptococcus sanguis*, SSP = *Staphylococcus saprophyticus*, ST = *Salmonella typhi*, STM = *Salmonella typhimurium*, VC = *Vibrio cholerae*, XM = *Xanthomonas maltophilia*, YE = *Yersenia enterocolitica*

GFPMA = Green Fluorescent Protein Reporter Microplate Assay. MDR = multi drug resistant, MIC = minimum inhibitory concentration, MIQ = minimum inhibition quantities, n.d. = not defined, IZD = inhibition zone diameter

Table S3: Natural products with antimicrobial activity from selected plants traditionally used against infective diseases of respiratory tract in Tanzania (see Table S1)

Plants with high activity (MIC ≤ 50 μg/mL, IZD ≥ 20 mm) are in marked bold.

S. No.	Family	Plant name	Bioactivity of compound against tested microorganism	Book or Journal reference
1.	Anacardiaceae	***Mangifera indica* L.**	**Mangiferin** (20%): activity against **BP (IZD = 20 ± 0.40 mm)**, BC (IZD = 17 ± 0.54 mm), SA (IZD = 17 ± 0.40 mm), SC (IZD = 15 ± 0.20 mm), **SLA (IZD = 26 ± 0,30 mm)**, EC (IZD = 12 ± 0.20 mm at 35 % mangiferin, IZD = 0 mm at lower conc.), **KP (IZD = 20 ± 0.40 mm** at 30 % mangiferin, IZD = 0 mm at lower conc.), no activity of PA at any conc.	[43]
			**Mangiferin** (25%): activity against **BP (IZD = 23 mm)**, BC (IZD = 18 mm), **SLV (IZD = 29 mm)**, no activity against PA.	[44]
2.		***Ozoroa mucronata* (Bernh.) R. Fern & A. Fern.**	**Moronic acid**: activity against **SA (MIC = 6.25 μg/mL)** and **BS (MIC = 12.5 μg/mL)**; no activity against PV, EC, and PA.	[114]
3.		***Rhus natalensis* Bernh. ex C. Krauss. F.A.Barkley)**	**Rhuschromone** (3-(1-(2,4-dihydroxyphenyl)-3,3-bis(4-hydroxyphenyl)-1-oxopropan-2-yl)-7-methoxy-4H-chromen-4-one): activity against **SA (IZD = 21 mm)**, no activity against EC and PA.<br>**2′, 4′-Dihydroxychalcone-(4-O-5′′′)-4′′,2′′′,4′′′-Trihydroxychalcone**: activity against SA (IZD = 17 mm), no activity against EC and PA.<br>**3-((Z)-Heptadec-13-enyl) benzene-1, 2-diol**: activity against SA (IZD = 9 mm), no activity against EC and PA.	[267]
			**Epicatechin**: activity against BS (IZD = 14 ± 0.2 mm), SA (IZD = 15 ± 0.3 mm), PA (IZD = 10 ± 0.2 mm), and EC (IZD = 11 ± 0.3 mm).<br>**3β-Sitosterol**: activity against BS (IZD = 7 ± 0.3 mm), SA (IZD = 8 ± 0.2 mm), no activity against PA and EC.<br>**3β-Sitosterol glucoside**: activity against BS (IZD = 13 ± 0.3 mm), SA (IZD = 14 ± 0.2 mm), and EC (IZD = 10 ± 0.2 mm), no activity against PA.<br>**Stigmasterol**: activity against BS and SA (IZD = 8 ± 0.2 mm each), no activity against PA and EC.<br>**Lupeol**: activity against BS (IZD = 11 ± 0.2 mm), SA (IZD = 14 ± 0.4 mm), and EC (IZD = 8 ± 0.1 mm), no activity against PA.	[268]
4.	Annonaceae	***Annona senegalensis* Pers.**	**Catechin**: weak activity against SM (MIC = 2.0 mg/mL).<br>**Anonaine**: significant activity SM (MIC = 0.12 mg/mL)<br>**Asimilobine**: weak activity against SM (MIC = 2.0 mg/mL).<br>**Nornantenine**: activity against SM (MIC = 0.25 mg/mL).	[269]

S. No.	Family	Plant name	Bioactivity of compound against tested microorganism	Book or Journal reference
			**Kaurenoic acid** (kaur-16-en-19-oic acid): activity against **BS** (IZD = 16 ± 0 mm, **MIC = 30 μg/mL**), SA (IZD = 17 ± 0 mm, MIC = 150 μg/mL), no activity against PA, SPT, and EC.	[270]
5.	Apocynaceae	***Rauvolfia caffra* Sond.**	**Rauvolfianine**; activity against isoniaIZD resistant strain **MTB AC 45 (MIC = 7.8125 μg/mL)**.<br>**Oleanolic acid**: activity against **MTB AC45 (MIC = 31.25 μg/mL)**.<br>**Sitosterol-3-O-β-D-glucopyranoside**: activity against MTB AC45 (MIC = 62.5 μg/mL).<br>**Betulinic acid**: activity against MTB AC45 (MIC = 62.5 μg/mL).<br>**D-Fructofuranosyl-β-(2→1)-α-D-glucopyranoside:** activity against MTB AC45 (MIC = 125 μg/mL).	[271]
6.	Bignoniaceae	***Kigelia africana* (LAM.) BENTH.**<br>(Syn. *Kigelia pinnata* (Jacq.) DC	**Kigelinone**: activity against SA and BS (MIC = 100 μg/mL each), **CBD (MIC = 50 μg/mL)**.<br>**Isopinnatal, dehydro-α-lapachone, lapachol, *p*-coumaric acid, ferulic acid, caffeic acid**: all active against SA and BS (MIC = 200 μg/mL each), and CBD (MIC = 100 μg/mL).	[272]
			**Kojic acid**; activity against EC (IZD = 6 mm, MIC = 0.83 mg/mL), PA (IZD = 8 mm, MIC = 0.21 mg/mL), ST (IZD = 6 mm, MIC = 0.10 mg/mL), and SA (IZD = 11 mm, MIC = 0.21 mg/mL).<br>**2-acetylfuro-1,4-naphthoquinone**: activity against PA and SA (IZD = 6 mm each), no activity against EC and ST.<br>***p*-Coumaric acid**: activity against EC, PA, SA (IZD = 6 mm, MIC > 0.83 mg/mL), and ST (IZD = 7 mm, MIC = 0.41 mg/mL).<br>**Caffeic acid**: activity against EC (IZD = 6 mg/mL, MIC > 0.83 mg/mL), no activity against PA, SA, and ST.<br>**Nonacosanoic acid**: activity against EC and PA (IZD = 6 mm each, MIC > 0.83 mg/mL each), no activity against ST and SA.<br>**Kigelinol**: activity against EC (IZD = 6 mm), and SA (IZD = 8 mm), no activity against PA and ST.<br>**β-Friedelinol**: activity against PA and SA (IZD = 6 mm, MIC > 0.83 mg/mL), no activity against EC and ST.<br>**Pomolic acid**: activity against PA (IZD = 7 mm), no activity against EC, ST, and SA.<br>(IZD measured at 2 mg/disc)	[131]
7.		***Stereospermum kunthianum* Cham.**	**Ipolamiide**: activity against **SA, SPN, KP, EC, and ST (IZD = 21–29 mm each)**, no activity against SD and SA MR.	[273]
8.	Boraginaceae	*Cordia africana* Lam.	**Oleanolic acid**: moderate activity (IC_{50} = 14.44 μg/mL) against vancomycin-resistant ECF; no activity against SA MR, EC, PA, and KP.<br>**13 3-β-Lup-20(29)-en3-ol, stigmast-5,22-dien-3β-ol, 2-(2Z) -(3-hydroxy-3,7-dimethylocta-2,6-dienyl)-1,4-benzenediol, 17 benzaldehyde derivative, 4-hydroxy-3-methoxy-benzaldehyde, isoflavone 7-hydroxy-4′-methoxyisoflavone** and **ubiquinone-8, 1-octacosanol**: no activity against vancomycin-resistant ECF, SA MR, EC, PA, and KP.	[274]

S. No.	Family	Plant name	Bioactivity of compound against tested microorganism	Book or Journal reference
9.	Canellaceae	***Warburgia salutaris* (Bertol.f.) Chiov.**	**Drimenol**: activity against KP (MIC = 0.208 mg/mL), MC and SA (MIC = 0.062 mg/mL each), PA (MIC = 0.312 mg/mL).<br>**E-nerolidol**: activity against KP (MIC = 0.312 mg/mL), **MC (MIC = 0.031 mg/mL)**, PA (MIC = 0.260 mg/mL), and SA (MIC = 0.416 mg/mL).<br>**12α-Acetal-polygolial**: activity against KP (MIC = 0.156 mg/mL), MC and PA (MIC = 0.625 mg/mL each), and SA (MIC = 0.521 mg/mL).<br>**Polygolial**: activity against **KP (MIC = 0.025 mg/mL)**, no activity against MC; PA, and SA (MIC > 0.25 mg/mL each).<br>**Ugandensidial**: activity against KP and SA (MIC = 0.130 mg/mL each), MC (MIC = 0.104 mg/mL), and PA (MIC = 0.078 mg/mL).<br>**Warburganal**: activity against KP (MIC = 0.130 mg/mL), MC (MIC = 0.208 mg/mL), PA (MIC = 0.104 mg/mL), and SA (MIC = 0.156 mg/mL).	[146]
10.		***Warburgia ugandensis* Sprague**	**4(13),7-Coloratadien-12,11-olide**: activity against MA (MIC = 128 μg/mL), MF (MIC = 128 μg/mL), MS (MIC = 128 μg/mL), and not active against MP<br>**Muzigadial**: activity against **MA (MIC = 32 μg/mL)**, **MF (MIC = 16 μg/mL)**, MS (MIC = 64 μg/mL), and MP (MIC = 64 μg/mL)<br>**Muzigadiolide:** activity against MA (MIC = 128 μg/mL), MF (MIC = 128 μg/mL), MS (MIC = 128 μg/mL), and MP (MIC = 64 μg/mL)<br>**Linoleic acid**: activity against **MA (MIC = 4 μg/mL)**, **MF (MIC = 8 μg/mL)**, **MS (MIC = 16 μg/mL)**, and **MP (MIC = 4 μg/mL)**<br>(displayed only substances possessing activity against all mycobacteria tested)	[45]
			**7α-Acetoxyugandensolide**: activity against RS (MIC = 200 μg/mL), no activity against SI.<br>**Bemadienolide** and **Ugandensolide**: activity against SI (MIC = 100 μg/mL), no activity against RS.<br>**Polygodial, mukaadial** and **muzigadial**: activity against **RS (MIC = 25 μg/mL)** and **SI (MIC = 50 μg/mL)**.<br>**Warburganal**: activity against **RS and SI (MIC = 50 μg/mL each)**.<br>**Ugandensidial**: activity against RS (MIC = 100 μg/mL), and **SI (MIC = 25 μg/mL)**.<br>**6α-Hydroxymuzigadial**: activity against RS (MIC = 100 μg/mL), no activity against SI.<br>**Deacetoxyugandensolide**: activity against RS (MIC = 100 μg/mL), and SI (MIC = 200 μg/mL).<br>**Drimenin, 9-deoxymuzigadial, cinnamolide,** and **3β-acetoxycinnamolide**: no activity against SI and RS.	[275]
11.	Celastraceae	***Elaeodendron buchananii* (Loes.) Loes.**	**3β-Acetoxy-11α, 19α, 28-trihydroxyurs-12-en-23-oic acid**: activity against SAL (MIC = 125 μg/mL), no activity against SA, SP, and EC (MIC = 250 μg/m L each), and VC, SD, and NM (MIC > 250 μg/mL each).	[34]

S. No.	Family	Plant name	Bioactivity of compound against tested microorganism	Book or Journal reference
			**Elaeodendroside A**: activity against SA, SP, and EC (MIC = 125 µg/mL each), no activity against SAL, SD, and NM (MIC = 250 µg/mL each) and VC (MIC > 250 µg/mL).<br>**Elaeodendroside B**: activity against SP and EC (MIC = 125 µg/mL each), no activity against SA, SAL, VC, SD, and NM (MIC = 250 µg/mL each).<br>**Ursolic acid**: activity against SA and SAL (MIC = 62.5 µg/mL each), SP and EC (MIC = 125 µg/mL each), no activity against VC (MIC = 250 µg/mL), SD and NM (MIC > 250 µg/m L each).<br>**Canophyllol**: activity against **NM (MIC = 31.25 µg/mL)**, SA and SAL (MIC = 62.5 µg/mL each), SP, and EC (MIC = 125 µg/mL each), no activity against VC and SD (MIC = 250 µg/m L each).<br>**Friedelin**: activity against SA and SAL (MIC = 62.5 µg/mL each), SP. EC and NM (MIC = 125 µg/mL each), no activity against VC and SD (MIC > 250 µg/m L each).<br>**3β, 11α, 19α-Trihydroxyurs-12-en-23, 28-dioic acid, 3β-acetoxy-19α, 23, 28-trihydroxyurs-12-ene, 3-oxo-19α,28-dihydroxyurs-12-en-24-oic acid, umbilleferone, 3β-hydroxyfriedelin, coumarin, stigmasterol**: no activity against SA, SP, SAL, EC, VC, SD, and NM.<br>According to the authors, values of > 125 µg/mL were considered not active.	
12.		*Maytenus senegalensis* (Lam.) Excell.	**Maytenonic acid**: activity against BS, EC, and KP (MIC = 98 µg/mL each), and SA (MIC = 195 µg/mL).	[52]
13.		*Gymnosporia senegalensis* (Lam.) Loes (syn. *Maytenus senegalensis* (Lam.) Excell.)	**(2S)-1-O-(4′Z,7′Z,10′Z-octadecatrienoyl) glycerol** and **(2R)-methyl [(6′-O-galloyl)-β-D-glucopyranosyloxy]phenylacetate**: no growth inhibition of strains SA NBRC13276, BS NBRC 3134, and EC NBRC 3972 at 50 µM.	[42]
14.	Combretaceae	*Combretum adenogonium* Steud. ex A.Rich. (syn. *Combretum fragrans* F. Hoffm.)	**2,3,8-Trihydroxy-4,6-dimethoxyphenanthrene, 2,3,8-trihydroxy-4,6-dimethoxy-9,10-dihydrophenanthrene, derivative of 2,3,8-trihydroxy-4,6-dimethoxyphenanthrene condensation with methyl acetate, derivative of 2,3,8-trihydroxy-4,6-dimethoxy-9,10-dihydrophenanthrene condensation with methyl propionate**: activity against PA (MIC = 0.16 mg/mL each), no activity against EC and SA.	[276]
15.	Compositae	*Microglossa pyrifolia* (Lam.) Kunze	**2-β-D-Glucopyranosyloxy- 1 -hydroxy-trideca-3,5,7,9,1 1-pentayne**: activity against EC (MIC = 388 µg/mL), PA, EF, and SA ATCC 25923 (MIC = 194 µg/mL), and SA –ATCC 29213 (MIC = 97 µg/mL).<br>**3-β-D-Glucopyranosyloxy- 1-hydroxy-6(*E*)-tetradecene-8,10.12-triyne, 2- β-D-glucopyranosyloxy-1-hydroxy-3(*E*)-tridecene-5,7,9,1 1-tetrayne, 2- β-D-glucopyranosyloxy-1-hydroxy-5(*E*)-tridecene-7,9,1 1-triyne**: no activity against EC, PA, EF, and both strains of SA.	[277]

S. No.	Family	Plant name	Bioactivity of compound against tested microorganism	Book or Journal reference
16.		***Solanecio mannii* (Hook.f.) C. Jeffrey)** *(syn. Crassocephalum mannii* (Hook.f.) Milne-Redh.)	***n*-Hexacosanol**: activity against **SA** (IZD = 3 mm, **MIC = 6.3 μg/mL**), **SF** (IZD = 2 mm, **MIC = 12.5 μg/mL**), **BC** (IZD = 2 mm, **MIC = 6.3 μg/mL**), **PA** (IZD = 2 mm, **MIC = 12.5 μg/mL**), **SD** (IZD = 2 mm, **MIC = 12.5 μg/mL**), **SHF** (IZD = 2 mm, **MIC = 12.5 μg/mL**), no activity against BS, EC, and KP (IZD = 0 mm each).<br>(IZD measurement: 25 μg/disc)	[35]
17.		*Vernonia colorata* (Willd.) Drake	**Vernolide**: activity against SA (MIC = 0.5 mg/mL) and BS (MIC = 0.1 mg/mL); no activity against EC and KP (MIC > 8 mg/mL each).<br>**11β,13-Dihydrovernolide**: activity against BS (MIC = 4 mg/mL), no activity against SA, EC, and KP (MIC > 8 mg/mL).<br>**Vernodalin**: activity against SA (MIC = 0.25 mg/mL), BS (MIC = 0.1 mg/mL), EC and KP (MIC = 8 mg/mL each).	[278]
			**Vernolide**: activity against EC and SA (MIC = 0.3 mg/mL each), and EF (MIC = 0.4 mg/mL).	[279]
18.	Crassulaceae	***Bryophyllum pinnatum* (Lam.) Oken**	**Kaempferitrin**: activity against **SA, PA, and ST (MIC = 32 μg/mL each)**.<br>**Kaempferol 3-O-α-L-(3-acetyl)rhamnopyranoside-7-O-α-L-rhamnopyranoside**: activity against SA (MIC = 256 μg/mL), PA (MIC = 128 μg/mL), and ST (MIC = 64 μg/mL).<br>**Kaempferol 3-O-α-L-(4-acetyl)rhamnopyranoside-7-O-α-L-rhamnopyranoside**: activity against SA (MIC = 64 μg/mL), PA (MIC = 128 μg/mL), and **ST (MIC = 32 μg/mL)**.<br>**Kaempferol 3-O-α-D-glucopyranoside-7-O-α-L-rhamnopyranoside**: activity against **SA and PA (MIC = 4 μg/mL each)**, and **ST (MIC = 1 μg/mL)**.<br>**Afzelin**: activity against **SA (MIC = 8 μg/mL)**, **PA (MIC = 12 μg/mL)**, and **ST (MIC = 4 μg/mL)**.<br>**α-Rhamnoisorobin**: activity against **SA (MIC = 2 μg/mL), PA and ST (MIC = 1 μg/mL each)**.	[53]
			**5^{I} –Methyl-4^{I},5,7-trihydroxyflavone** (100 mg/mL): activity against SA (IZD = 7 mm, MIC = 12.5 mg/mL), PA (IZD = 8 mm, MIC = 2.5 mg/mL), KP (IZD = 8 mm, MIC = 5 mg/mL), no activity against EC.<br>**4^{I},3,5,7-Ttetrahydroxy -5-methyl-5^{I}-propenamine anthocyanidine** (100 mg/mL): activity against SA (IZD = 10 mm, MIC = 25 mg/mL), EC (IZD = 3 mm, MIC = 100 mg/mL), PA (IZD = 10 mm, MIC = 12.5 mg/mL), KP (IZD = 7 mm, MIC = 50 mg/mL).	[280]
			**1-ethanamino 7 Hex-1-yne-5^{I}-one phenanthrene** (100 mg/mL): activity against SA (IZD = 7.1 ± 0.01 mm, MIC = 50 mg/mL), EC (IZD =1.0 ± 0.01 mm, MIC = 100 mg/mL), PA (IZD = 3.2 ± 0.01 mm, MIC = 50 mg/mL), KP (IZD = 2.1 ± 0.01 mm, MIC = 100 mg/mL).	[281]

S. No.	Family	Plant name	Bioactivity of compound against tested microorganism	Book or Journal reference
19.	Dennstaedtiaceae	*Pteridium aquilinum (L.) Kuhn*	***p*-Coumaric acid**: activity against CBP and ECA (MIC = 100 ppm each), CBF (MIC = 500 ppm), and EAM (MIC = 50 ppm).<br>**Ferulic acid**: activity against CBP and CBF (MIC = 500 ppm each), EAM and ECA (MIC = 50 ppm each).<br>**Caffeic acid**: activity against CBP (MIC = 1000 ppm). CBF, EAM, and ECA (MIC = 100 ppm each).<br>***p*-Hydroxybenzoic acid**: activity against CBP (MIC = 1000 ppm), CBF (MIC = 500 ppm), ECA and EAM (MIC = 50 ppm each).	[282]
			**(2S)-Pteroside K**: moderate inhibition of 44.1% against MBM<br>**(2S)-Rhedynoside A**, **rhedynosin G**, **(2R)-pterosin**, **(2S)-pteroside A**, and **(2S)-pteroside A2**: moderate activity at a rate of 100 μM against **KP BAA 2146** compared to **(2S)-2-hydroxymethylpterosin** and **pteroside Z** (mild – moderate inhibition, 41.1-43.8%).<br>**(2S)-Pterosin D**: moderate inhibition of KP ATCC 13883 (46.6%).<br>**Rhedynosins B and D**: moderate inhibition of KP ATCC 13883 (40.1% and 40.5% respectively).<br>All compounds: no inhibition of SA MR at a rate of 100 μM.	[283]
20.	Ebenaceae	***Diospyros mespiliformis* Hochst. ex DC.**	**Diosquinone**: activity against **SA NCTC 6571 (MIC = 3 μg/mL)**, **SA E3T and BS (MIC = 5 μg/mL each)**, **SP and EC (MIC = 15 μg/mL each)**, **PA (MIC = 30 μg/mL)**, and **STM (MIC = 45 μg/mL)** at the highest conc. of inoculum (20 cfu per 20 μL applied);<br>**Plumbagin**: activity against SA NCTC 6571, and SA E3T (IZD: +++ each at conc. 400 μg), and EC (IZD: ++ at conc. 600 μg), no activity against PA.	[46]
21.	Euphorbiaceae	***Euphorbia hirta* L.**	**Caffeic acid**: activity against **PA (MIC = 31.3 μg/mL)**, no activity against SA, SD, ST, EC, and BS.<br>**(–)-Epicatechin 3-gallate**: activity against **PA (MIC = 15.6 μg/mL)**, no activity against SA, SD, ST, EC, and BS.	[284]
22.		***Jatropha curcas* L.**	**Methyl pheophorbide a analogue** (10 mg/mL): activity against MP (IZD = 14.7 ± 2.3 mm) and SRM (IZD = 19.0 ± 1.0 mm).<br>**Combination of pheophytin b analogue + methyl pheophorbide a analogue** (10 mg/mL): activity aginst **MP (IZD = 20.0 ± 0 mm)** and SRM (IZD = 14.3 ± 1.2 mm).	[184]
23.		***Spirostachys africana* Sond.**	**D-Friedoolean-14-en-oic acid (3-acetyl aleuritolic acid)**: activity against **SA, ST, VC, EC, and SHD (MIC = 50 μg/mL each)**, SHF and SHB (MIC = 100 μg/mL each), no activity against SHS (MIC > 200 μg/mL).<br>**Lupeol**: no activity against SA, ST, VC, EC, SHD, SHF, SHB, and SHS (MIC > 200 mg/mL each).<br>***ent*-2,6α-dihydroxy-norbeyer-1,4,15-trien-3-one (diosphenol 2)**: activity against SA, VC, SHD, and SHF (MIC = 100 μg/mL each), ST, EC, and SHB (MIC = 200 μg/mL each), no activity against SHS (MIC > 200 μg/mL).	[54]

S. No.	Family	Plant name	Bioactivity of compound against tested microorganism	Book or Journal reference
			***ent*-3β-hydroxy-beyer-15-ene-2-one**: activity against **SA, SHD, and SHF (MIC = 50 μg/mL each)**, ST, VC, EC, and SHB (MIC = 100 μg/mL each), no activity against SHS (MIC > 200 μg/mL).	
24.	Lamiaceae	***Hoslundia opposita* Vahl**	**Euscaphic acid**: active gainst **MTB H37Rv (MIC = 50 μg/mL)**.	[285]
25.		***Hyptis pectinata* (L.) Poit.**	**Hyptolide**: marginal activity against gram positive bacteria (BS: IZD = 5 mm, MIC = 100 μg/mL, no activity against SA, EC and PA)	[193]
			**Pectinolide A**: activity against **BS** (IZD = 10 mm, **MIC = 6.25 μg/mL**), and **SA** (IZD = 8 mm, **MIC = 12.5 μg/mL**).	[286]
			**Pectionolide A**: activity against **SA ATCC 25923 (MIC = 32 μg/mL)**, SA XU-212, SA SA-1199B and SA MR EMRSA-15 (MIC = 128 μg/mL each).	[287]
			**Pectionolide B**: activity against SA ATCC 25923 (MIC = 128 μg/mL), SA XU-212, SA SA-1199B and SA MR EMRSA-15 (MIC = 256 μg/mL each).	
			**Pectionolide C**: activity against SA ATCC 25923 (MIC = 64 μg/mL), SA XU-212 (MIC = 256 μg/mL), and SA SA-1199B and SA MR EMRSA-15 (MIC = 128 μg/mL each).	
			**Pectionolide H**: activity against **SA ATCC 25923 (MIC = 32 μg/mL)**, SA XU-212, SA SA-1199B and SA MR EMRSA-15 (MIC = 64 μg/mL each).	
26.		*Ocimum suave* Willd.	**Eugenol**: activity against EC and ML (MIC = 600 μg/mL each), SA (MIC = 700 μg/mL), and SC (MIC = 400 μg/mL).	[195]
27.	Leguminosae (Fabaceae)	***Abrus precatorius L.***	**Abruquinone B**: activity against **MTB H37Ra (MIC = 12.5 ± 0.0 μg/mL)**.	[288]
28.		***Acacia mellifera* (M. Vahl) Benth.**	**3-(Z)-*cis* coumaroylbetulin, botulin** (0.1 mg/disc): activity against SA (IZD = 10 mm), PA (IZD = 14 mm), and MG (IZD = 19 mm).	[289]
			**30-Hydroxylup-20 (29)-en-3β-ol** (0.1 mg/disc): activity against SA (IZD = 13 mm), PA (IZD = 17 mm), no activity against MG.	
			**(20S)-oxolupane-30al**: activity against SA (IZD = 10 mm) and **MG (IZD = 21 mm)**.	[290]
			**(20R)-oxolupane-30-al**: activity against SA (IZD = 10 mm), no activity against MG.	
			**Betulinic acid**: activity against SA (IZD = 9 mm), no activity against MG.	
29.		***Cajanus cajan* (L.) Millsp.**	**Cajanuslactone**: activity against SE and BS (MIC = 0.125 mg/mL each), and **SA (MIC = 0.031 mg/mL)**.	[208]
			**Pinostrobin**: activity against no activity against SE, SA, and BS.	
			**Cajaninstilbene acid**: activity against **SE (MIC = 0.013 mg/mL)**, **SA (MIC = 0.025 mg/mL)**, and **BS (MIC = 0.025 mg/mL)**.	

S. No.	Family	Plant name	Bioactivity of compound against tested microorganism	Book or Journal reference
30.		***Cassia abbreviata* Oliv.**	**Cassinidin A**: activity against **EC (MIC = 1 μg/mL)**, **BS and SA (MIC = 0.5 μg/mL each)**.<br>**Cassinidin B**: activity against **EC (MIC = 10 μg/mL)**, **BS and SA (MIC = 5 μg/mL each)**.	[47]
31.		***Cassia singueana* Delile**	**Torosachrysone**: activity against SA (MIC = 70 μg/mL)<br>**Germichrysone**: activity against **CE (MIC = 30 μg/mL)**<br>**Singueanol-I**: activity against **SA (MIC = 25 μg/mL)**<br>**Singueanol-II**: activity against **SA (MIC = 50 μg/mL)**	[291]
32.		*Dichrostachys cinerea* (L.)	**Tannins**: activity against SA and PA (MIC = 5.5 mg/mL each), SHB (MIC = 4.5 mg/mL), SHF (MIC = 4.0 mg/mL), and EC (MIC = 5.0 mg/mL).	[292]
33.		***Entada abyssinica* A. Rich**.	**Ursolic acid**: activity against **SA (MIC = 12.5 μg/mL)**, **BC (MIC = 6.25 μg/mL)**, and STM (MIC = 100 μg/mL), no activity against PA, EF, and EC.<br>**Quercitrin**: activity against **BC (MIC = 12.5 μg/mL)**, **STM (MIC = 3.12 μg/mL)**, **PA and EC (MIC = 50 μg/mL each)**, and **EF (MIC = 25 μg/mL)**.<br>**Quercetin 3-O-β-d-glucosyl (1→4)-α-l-rhamnoside**: activity against **SA, STM, and EC (MIC = 25 μg/mL each)**, **BC, PA, and EF (MIC = 50 μg/mL)**.<br>**(8S)-Kolavic acid 15-methyl ester**: activity against **SA and BC (MIC = 25 μg/mL each)**, and STM (MIC = 100 μg/mL), no activity against PA, EF, and EC.<br>**Methyl gallate**: activity against **SA, BC and STM (MIC = 50 μg/mL each)**, no activity against PA, EF, and EC.<br>**Entadanin**: activity against **SA and EC (MIC = 12.5 μg/mL each)**, **BC and PA (MIC = 25 μg/mL each)**, and **STM (MIC = 1.56 μg/mL)**, no activity against EF.<br>**13,14,15,16-Tetranor-3-clerodene-12,18-dioic acid** and **Bis-[(S)-(2,3-dihydroxypropyl)] hexacosanedioate**: no activity against SA, BC, STM, PA, EF, and EC each.	[48]
			**8S-Kolavic acid 18-methyl ester**: activity against EC, *Proteus* sp., and SA (IZD = 13 mm at 250 μg each).<br>**8S-Kolavic acid 15-methyl ester** and **8S-kolavenol**: no activity against EC, *Proteus* sp., and SA.	[216]
34.		*Erythrina abyssinica* DC.	**2'methoxy-nor-glycyrrisoflavanone**: activity against SA (IZD = 15 mm), no activity against PA.<br>**1,5,4′trihydroxy-5′-prenylchalcone** and **2',3′,4′,7-tetrahydroxy-5'-prenylflavanone**: no activity against SA an PA.	[293]
35.	Loganiaceae	*Strychnos spinosa* Lam.	**Sarracenin**: activity against **SA, EC, SD, and KP (MIC = 2.5 μg/mL), SP, ST, and PA (MIC = 5 μg/mL)**.	[49]
36.	Malvaceae	*Sida rhombifolia* L.	***n*-Hexacos-11-enoic acid** (50 mg/mL): activity against EC (IZD = 13 mm), SA (IZD = 12 mm), PA (IZD = 8 mm), and STM (IZD = 11 mm).	[294]

S. No.	Family	Plant name	Bioactivity of compound against tested microorganism	Book or Journal reference
			**Stigmasterol** (50 mg/mL): activity against EC (IZD = 14 mm), SA (IZD = 12.5 mm), PA (IZD = 9.5 mm), and STM (IZD = 13 mm).<br>**β-Sitosterol** (50 mg/mL): activity against EC (IZD = 13.5 mm), SA (IZD = 11 mm), PA (IZD = 8.5 mm), and STM (IZD = 12 mm).	
			**1,3-dilinoleoyl-2-oleine** (50 mg/mL): activity against PA (IZD = 9 mm), SA (IZD = 8 mm), no activity against STM and EC.<br>***n*-hexacosa-11-enoic acid** (50 mg/mL): activity against PA (IZD = 10 mm), SA (IZD = 14 mm), TPM (IZD = 15 mm), and EC (IZD = 11 mm).	[295]
37.	Moraceae	***Milicia excelsa* (Welw.) C.C. Berg**	**Cudraxanthone** activity against SA ATCC 25923 (IZD = 9.0±0.1 mm), SA ATCC 29213 (IZD = 7.6 ± 0.0 mm), no activity against EC ATCC 35218 and EC ATCC 25322.<br>**Neocyclomorusin** activity against SA ATCC 25923 (IZD = 7.5±0.1 mm), SA ATCC 29213 (IZD = 6.7 ± 0.1 mm), no activity against EC ATCC 35218 and EC ATCC 25322.<br>**(9βH)-3β-acetoxylanosta-7,24-diene** activity against SA ATCC 25923 (IZD = 6.2±0.2 mm), SA ATCC 29213 (IZD = 6.0 ± 0.1 mm), no activity against EC ATCC 35218 and EC ATCC 25322.	[50]
			**Atalantoflavone** activity against EC ATCC 8739 and EA ATCC 13048 (MIC = 256 µg/mL each), PS PS299645 (MIC = 128 µg/mL), no activity against EC AG102, EC AG100Atet, EA CM64, EA EA27, KP ATCC11296, KP KP55, PS ATCC29916, EBC BM47, EBC BM67, PA PA01, and PA PA124.<br>**2′-Hydroxyatalantoflavone** activity against EC ATCC 8739, EA ATCC 13048 and PS PS299645 (MIC = 256 µg/mL each), no activity against EC AG102, EC AG100Atet, EA CM64, EA EA27, KP ATCC11296, KP KP55, PS ATCC29916, EBC BM47, EBC BM67, PA PA01, and PA PA124.<br>**Neocyclomorusin** activity against **EC ATCC 8739, EA ATCC 13048, KP KP55, and PS ATCC29916 (MIC = 8 µg/mL), EC AG102, EC AG100Atet, EA CM64 , PS PS299645, and PA PA01 (MIC = 32 µg/mL each), EA EA27 and EBC BM67 (MIC = 16 µg/mL each), KP ATCC11296 and EBC BM47 (MIC = 4 µg/mL each)**, and PA PA124 (MIC = 64 µg/mL).<br>**2-(3,5-dihydroxyphenyl)benzofuran-5,6-diol** activity against EC ATCC 8739, EC AG102, EA ATCC 13048, EA CM64, EA EA27, PS ATCC29916, PS PS299645, and EBC BM47(MIC = 256 µg/mL), no activity against EC AG100Atet, KP ATCC11296, KP KP55, EBC BM67, PA PA01, and PA PA124.	[296]
38.	Moringaceae	***Moringa oleifera* Lam.**	All compounds had very high bactericidal activity at 5 mg/L against some of test pathogens SD, BC, EC, and ST.<br>**4-(β-d-glucopyranosyl-1→4-α-l-rhamnopyranosyloxy)-benzyl thiocarboxamide**: most potent with 99.2% inhibition toward SD and 100 % toward BC, EC, and ST within 4 h of contact.	[297]

S. No.	Family	Plant name	Bioactivity of compound against tested microorganism	Book or Journal reference
			**4-(α-l-rhamnopyranosyloxy)benzyl isothiocyanate**: activity against BC var. *mycoides*, BM, BC, MA, ML, and MP (IZD = 10-15 mm each), PM and SF (IZD = 5-10 mm each), EC and SED (IZD = 2-5 mm each), and SM (IZD = 1-2 mm), no activity against KP.	[298]
			**4-(α-L-Rhamnosyloxy)benzyl isothiocyanate** (10 mg/mL): activity against **SA (IZD = 21.13 ± 1.48 mm)**, SE (IZD = 19.70 ± 0.95 mm), BS (IZD = 14.23 ± 0.84 mm), no activity against EC, EA, KP, and PA.<br>**4-(4′-O-Acetyl-α-L-rhamnosyloxy)benzyl isothiocyanate**(10 mg/mL): activity against **SA (IZD = 23.10 ± 0.44 mm), SE (IZD = 22.00 ± 0.50 mm)**, BS (IZD = 14.50 ± 1.04 mm), no activity against EC, EA, KP, and PA	[299]
39.	Myrtaceae	*Psidium guajava* L.	**Morin-3-*O*-α-L-lyxopyranoside** activity against SEN (MIC = 200 μg/mL), and BC (MIC = 250 μg/mL.)<br>**Morin-3-*O*-α-L-arabopyranoside** activity against SE (MIC = 200 μg/mL), and BC (MIC = 300 μg/mL).	[51]
			**Pentapentacont-17,31-diol**: activity against SA (IZD = 9-11 mm), *Shigella* spp., EC, KP, and BS (IZD = 6.8-8.0 mm each), no activity against STM and PA.<br>**11-Hydroxy-tricont-35- pentatriacontanoate**: activity against SA (IZD = 12-14 mm) and Shigella spp. (IZD = 6.8-8.0 mm), no activity against STM, EC, KP, PA, and BS.<br>**34-Octahexacontanol**: activity against SA (IZD = 9-11 mm), EC, KP, and BS (IZD = 6.8-8.0 mm each), no activity against *Shigella* spp., STM, and PA.<br>**Heptatriacont-8-ol**: activity against SA (IZD = 9-11 mm), EC and KP (IZD = 6.8-8.0 mm each), no activity against *Shigella* spp., STM, PA, and BS.<br>**14,15-Dimethyl (cyclopropayl)-9-oloctadecayl-3-(4-hydroxyphenyl) propanoate**: activity against SA (IZD = 9-11 mm), *Shigella* spp. and PA (IZD = 6.8-8.0 mm each), no activity against STM, EC, KP, and BS.<br>**Hexaeicosan-16-ol**: activity against SA (IZD = 12-14 mm), *Shigella* spp., KP, and BS (IZD = 6.8-8.0 mm each), no activity against STM, EC, and PA.<br>**Pentatetracosan-10,25-diol**: activity against SA (IZD = 12-14 mm), *Shigella* spp. (IZD = 6.8-8.0 mm each), no activity against STM, EC, KP, PA and BS.<br>**Untricontan-11,19-diol**: activity against SA (IZD = 9-11 mm), KP (IZD = 6.8-8.0 mm), and no activity against *Shigella* spp., STM, EC, PA, and BS.<br>**Tricosan-17-ene-5-ol**: activity against SA (IZD = 12-14 mm), EC and PA (IZD = 6.8-8.0 mm each), no activity against *Shigella* spp. STM, KP, and BS.<br>**Nonacosan-23-ene-3-ol**: activity against SA (IZD = 12-14 mm), *Shigella* spp., STM, EC, and KP (IZD = 6.8-8.0 mm each), no activity against PA and BS.	[295]
40.	Phyllanthaceae	*Antidesma venosum* E. Mey. ex.Tul.	[redacted]**3R,4R,5S)-4-Hydroxy-5-methyl-3-tetradecanyl γ-lactone**: no activity against SA, SP, EF, BC, EC, PA, and SHF.	[300]

S. No.	Family	Plant name	Bioactivity of compound against tested microorganism	Book or Journal reference
			**Friedelin**: activity against SA and SP (MIC = 0.1875 mg/mL each), EC and SHF (MIC = 0.375 mg/mL each), PA (MIC = 0.75 mg/mL), .<br>**Lupeol**: activity against SA and SP (MIC = 1.25 mg/mL each), , no activity against EF, BC, EC, PA, and SHF.<br>**β-Sitosterol**; no activity against SA, SP, EF, BC, EC, PA, and SHF.	
41.		*Margaritaria discoidea* (Baill.) G. L. Webster	**Hydroxygenkwanin-8-C-[α-rhamnopyranosyl-(1 → 6)]-β-glucopyranoside**: activity against BS, SA, PV, and PA (MIC= 500 μg/mL each).<br>**Genkwanin-6-C-[α-rhamnopyranosyl-(1 → 6)]-β-glucopyranoside**: activity against BS, SA, PV, and PA (MIC= 500 μg/mL each).<br>**Kaempferol-3-O-α-rhamnopyranosyl-(1 → 2)-β-glucopyranoside-7-O-α-rhamnopyranoside**: activity against PV (MIC= 500 μg/mL), no activity against BS, SA, and PA (MIC > 500 μg/mL).<br>**Kaempferol-3-O-α-rhamnopyranosyl-(1 → 2)-[α-rhamnopyranosyl-(1 → 6)]-β-glucopyranoside-7-O-α-rhamnopyranoside**: activity against PV (MIC= 500 μg/mL), no activity against BS, SA, and PA (MIC > 500 μg/mL).	[301]
42.	Polygalaceae	*Securidaca longipedunculata* Fresen.	**(4-Methoxy-benzo[1,3]dioxol-5-yl)-phenylmethanone**: activity against PA (IZD = 4 ± 1.7 mm), no activity against SA and EC.<br>**1,7-Dihydroxy-4-methoxyxanthone**: activity against SA (IZD = 15.0 ± 0.7 mm), no activity against EC and PA.<br>**Benzyl 2-hydroxy-6-methoxybenzoate** and **methyl 2-hydroxy-6-methoxybenzoate**: no activity against SA, EC, and PA.<br>IZD determination: 10 mg compound/disc	[242]
			**3-Hydroxy benzoic acid** and **3-hydroxy- 4-methoxy benzoic acid**: no activities against BS, SA, EC, and PA.<br>**3, 4-Dimethoxy-7-hydroxyxanthone**: activity against BS and EC (IZD = 15 mm each), SA and PA (IZD = 11 m each).	[243]
43.	Rubiaceae	*Crossopteryx febrifuga* (Afzel. ex G. Don)	**Mixture of 18-epi-3β-urs-12,20(30)-diene-27,28-dioic acid and 3β-urs-12,20(30)-diene-27,28-dioic acid (1:3 ratio)**: activity against EA and EC (MIC = 128 μg/mL each), and KP and SA (MIC = 64 μg/mL each).<br>**Mixture of 18-epi-3β-D-glucopyranosyl-urs-12,20(30)-diene-27,28-dioic acid and 3β-D-glucopyranosyl-urs-12,20(30)-diene-27,28-dioic acid (1:3 ratio)**: activity against EA (MIC = 64 μg/mL), **EC and SA (MIC = 32 μg/mL each)**, and **KP (MIC = 8 μg/mL)**.	[302]
44.		***Pavetta crassipes* K. Schum.**	**Quercetin-3-O-rutinoside**: activity against EC, PA, and CU (MIC = 6.25 mg/mL each), SP, KP, and NG (MIC = 12.5 mg/mL each).	[303]
			**β-Sitosterol**: no activity against MTB H37Rv.	[304]

S. No.	Family	Plant name	Bioactivity of compound against tested microorganism	Book or Journal reference
			**Ursolic acid**: activity against MTB H37Rv (MIC = 200 µg/mL according to broth microdilution method, 0 µg/mL according to GFPMA).<br>**Methyl chlorogenate**; activity against MTB H37Rv (MIC = 100 µg/mL according to broth microdilution method, 200 µg/mL according to GFPMA).<br>**Ethyl chlorogenate**: activity against **MTB H37Rv (MIC = 50 µg/mL** according to broth microdilution method, 100 µg/mL according to GFPMA).<br>**Rutin and mannitol**: no activity against MTB H37Rv.	
45.		***Rubia cordifolia* L.**	**Rubiacordone A** (20 µg / disc): activity against SA (IZD = 13 ± 0.3 mm), **BS (IZD = 26 ± 0.3 mm)**, SE (IZD = 14 ± 0.4 mm), **SF (IZD = 29 ± 0.2 mm)**, **BC (IZD = 24 ± 0.6 mm)**, VP (IZD = 8 ± 0.5 mm), PA (IZD = 9 ± 0.5 mm), STM (IZD = 12 ± 0.2 mm), and EC (IZD = 10 ± 0.4 mm).<br>**1-Acetoxy-6-hydroxy-2-methylanthraquinone-3-O-[α-L-rhamnopyranosyl-(1→2)-β-D-glucopyranoside]** (20 µg / disc): activity against SA (IZD = 19 ± 0.6 mm), **BS (IZD = 27 ± 0.3 mm)**, SE (IZD = 11 ± 0.5 mm), **SF (IZD = 22 ± 0.5 mm)**, **BC (IZD = 26 ± 0.5 mm)**, VP (IZD = 11 ± 0.3 mm), PA (IZD = 8 ± 0.6 mm), STM (IZD = 8 ± 0.3 mm), and EC (IZD = 11 ± 0.5 mm).	[41]
46.	Rutaceae	*Harrisonia abyssinica* Oliv	**Harronin I (5-(ethan-1′′′-one)-4,6-dihydroxy-7-(3′′,3′′-dimethylallyl)-2S-(1′S-hydroxy-1′,5′-dimethylhex-4′-enyl)-2,3-dihydrobenzofuran):** activity against BC (IZD = 14 ± 1 mm), EC (IZD = 13 ± 2 mm), STM, SA, and LC (IZD = 12 ± 1 mm each).<br>**Harronin II (5-(2′′′-hydroxyethan-1′′′-one)-4,6-dihydroxy-7-(3′′,3′′-dimethylallyl)-2S-(1′S-hydroxy-1′,5′-dimethylhex-4′-enyl)-2,3-dihydrobenzofuran):** activity against BC (IZD = 10 ± 2 mm), EC (IZD = 11 ± 1 mm), STM (IZD = 9 ± 1 mm), SA (IZD = 8 ± 1 mm), and LC (IZD = 10 ± 1 mm).	[256]
47.		***Toddalia asiatica* (L.) Lam.**	**Flindersine**: activity against **BS and EF (MIC = 31.25 µg/mL each)**, SA and SE (MIC = 62.5 µg/mL), PA (MIC = 250 µg/mL), no activity against EC, KP, PV and EAM.	[261]
			**Ulopterol**: activity against BS (MIC = 250 µg/mL), SPT, PA, STM, ML, and SA (MIC = 125 µg/mL each), SHF and EA (MIC = 62.5 µg/mL), and **SE (MIC = 15.625 µg/mL)**.	[305]
			**Chelerythrine** (IZD at 52 µg/disc): activity against SA (IZD = 19.07 ± 0.19 mm, MIC = 0.156 mg/mL), SA MR (IZD = 18.12 ± 0.14 mm, MIC = 0.156 mg/mL), SA ESBLs (IZD = 16.93 ± 0.23 mm, MIC = 0.156 mg/mL).	[258]
			**Nelumol A**: activity against **MTB H37Ra (MIC = 50 µg/mL)**.<br>**8-Geranyloxy-5,7-dimethyloxycoumarin**: activity against **MTB H37Ra (MIC = 50 µg/mL)**.<br>**Norchelerythrine**: activity against **MTB H37Ra (MIC = 25 µg/mL)**.<br>**8-(3′,7′-Dimethyl-7′-hydroxy-2′E,5′E-octadienyl)oxy-5,7-dimethoxycoumarin, 8-(3′,7′-dimethyl-7′-hydroxy-2′E,5′Z-octadienyl)oxy-5,7-dimethoxycoumarin, 6-(3′-methyl-1′,3′-butadienyl)-5,7-dimethoxycoumarin, 4-O-geranylco-niferyl aldehyde, phellopterin , 5-methoxy-8-geranyloxylpsoralen, artanin, 5,7,8-trime-**	[306]

S. No.	Family	Plant name	Bioactivity of compound against tested microorganism	Book or Journal reference
			**thoxycoumarin, leptodactylone, toddalolactone, toddalolactone methyl ether, 1,2-seco-dihydromethylumbelliferone methyl ester and toddalosin**: no activity against MTB H37Ra.	
			**1-Dimethyl dicentrinone and dicentrinone**: activity against **SA (IZD = 8-24 mm**, MIC = 0.39-1.23 mg/mL) and **SE (IZD = 8-23 mm**, MIC = 0.12-1.31 mg/mL), no activity against EC, EBC, KP, PA, SD, SM, and SV.<br>**11-Hydroxy-10-methoxy-(2,3)-methylenedioxytetrahy-droprotoberberine**: activity against EC (IZD = 8 mm), **SA (IZD = 8-24 m**m, MIC = 0.39-1.23 mg/mL), **SE (IZD = 8-23 mm**, MIC = 0.12-1.31 mg/mL), KP and SD (IZD = 7 mm each), no activity against EBC, PA, SM, and SV.<br>**(2,3,10,11)-Dimethylenedioxytetrahydroprotoberberine**: activity against EC and KP (IZD = 7 mm each), **SA (IZD = 8-24 mm**, MIC = 0.39-1.23 mg/mL), **SE (IZD = 8-23 mm**, MIC = 0.12-1.31 mg/mL), SD (IZD = 8 mm), no activity against EBC, PA, SM, and SV.<br>**8-Methoxynorchelerythrin, 1-demethylrhoifoline, 8-methoxynitidine, 8-acetylnorchelerythrine, 8,9,10,12-tetramethoxynorchelerythrin, isointegriamide, rhoifoline B, pancorine, 8-methoxychelerythrine, arnottianamide, oxynitidine, oxysanguinarine, skimmianine and 5-methoxydictamnine**: activity against **SA (IZD = 8-24 mm**, MIC = 0.39-1.23 mg/mL), **SE (IZD = 8-23 mm**, MIC = 0.12-1.31 mg/mL), **EC (IZD = 10-24 mm)**, **EBC (IZD = 9-23 mm)**, **KP (IZD = 8-23 mm)**, **PA (IZD = 7-24 mm)**, **SD (IZD = 8-24 mm)**, **SM (IZD = 9-24 mm)**, **SV (IZD = 7-23 mm)**.<br>The most active substance was **8-acetylnorchelerythrine** giving the largest zones of inhibition in all cases.	[307]
48.	Verbenaceae	*Lantana camara* L.	**Acetyl linaroside**: activity against MTB H37Rv (MIC > 6.25 μg/mL).<br>Linaroside and lantanoside: no activity against MTB H37Rv.	[308]
49.	Xanthorrhoeaceae	***Aloe secundiflora* Engl.**	**5-hydroxy-3,6-dimethoxy-2-methylnaphthalene-1,4-dione**: activity against **MTB H37Rv (MIC = 23.5 μg/mL** determined by the Microplate Alamar Blue Assay (MABA) and **MIC = 23.1 μg/mL** determined by the Low Oxygen Recovery Assay(LORA)).<br>**Aloesaponarin I**: activity against **MTB H37Rv (MIC = 22.8 μg/mL** (MABA) **and MIC = 21.1 μg/mL** (LORA)).<br>**Aloesaponarin II, aloe-emodin** and **laccaic acid D methyl ester**: no activity against MTB H37Rv.	[309]

BC = *Bacillus cereus*, BM = *Bacillus megaterium*, BP = *Bacillus pumilus*, BS = *Bacillus subtilis*, CBD = *Corynebacterium diphtheria*, CBF = *Corynebacterium fasciens*, CBP = *Corynebacterium poinsettiae*,,CE = *Corynebacterium equi*, CU = *Corynebacterium ulcerans*, EAM = *Erwinia amylovora*, EBC = *Enterobacter cloacae*, EC = *Escherichia coli*, ECA = *Erwinia carotovora*, ECF = *Enterococcus faecium*, EF = *Enterococcus faecalis*, KP = *Klebsiella pneumoniae,* LC = *Lactobacillus cassei,* MA = *Mycobacterium aurum,* MBM = *Mycobacterium marinum,* MC = *Moraxella catarrhalis,* MF = *Mycobacterium fortuitum,* MG = *Microsporum gypseum,* ML = *Micrococcus luteus,* MP = *Mycobacterium phlei,* MS = *Mycobacterium smegmatis,* MTB = *Mycobacterium tuberculosis*, NG = *Neisseria gonorrheoeae* NM = *Neisseria meningitides,* PA = *Pseudomonas aeruginosa*, PM = *Proteus mirabilis*, PV = *Proteus vulgaris*, RS = *Ralstonia solanacearum*, SA = *Staphylococcus aureus*, SA MR = Methicillin resistant *Staphylococcus aureus*, SAG = *Streptococus agalactiae*, SAL = *Staphylococcus albus,* SCS = *Staphylococcus saprophyticus,* SC = *Staphylococcus citreus,* SD = *Shigella dysenterae,* SEN = *Salmonella enteriditis,* SE = *Staphylococcus epidermidis*, SED = *Salmonella edinburg*, SF =

Streptococcus faecalis, SHB = *Shigella boydii*, SHF = *Shigella flexneri*, SHS = *Shigella sonnei*, SI = *Streptomyces ipomoeae*, SLA = *Salmonella agona*, SLV = *Salmonella virchow*, SM = *Streptococcus mutans*, SP = *Streptococcus pyogenes*, SPN = *Streptococcus pneumoniae*, SPT = *Salmonella paratyphi*, SRM = *Serratia marcescens*, SSC = *Staphylococcus sciuri*, ST = *Salmonella typhi*, STM = *Salmonella typhimurium*, SV = *Streptococcus viridans*, SX = *Staphylococcus xylosus*, VC = *Vibrio cholera*, VP = *Vibrio parahaemolyticus*

MIC = minimum inhibitory concentration, IZD = inhibition zone diameter

References in Supplementary material to "An Annotated Inventory of Tanzanian Medicinal Plants Traditionally Used for the Treatment of Respiratory Bacterial Infections"

63. Moshi, M.J.; Otieno, D.F.; Mbabazi, P.K.; Weisheit, A. The Ethnomedicine of the Haya people of Bugabo ward, Kagera Region, north western Tanzania. *J. Ethnobiol. Ethnomed.* **2009**, *5*, 24.
64. Moshi, M.; Innocent, E.; Otieno, J.; Magadula, J.; Nondo, R.; Otieno, D.; Wensheit, A.; Mbabazi, P. Antimicrobial and brine shrimp activity of *Acanthus pubescens* root extracts. *Tanzan. J. Health Res.* **2010**, *12*, 155–158.
65. Orodho, J.A.; Kirimuhuzya, C.; Otieno, J.N.; Magadula, J.J.; Okemo, P. Local management of tuberculosis by traditional medicine practitioners in Lake Victoria Region. *Complement. Med. Res.* **2011**, *3*, 1–9.
66. Hedberg, I.; Hedberg, O.; Madati, P.J.; Mshigeni, K.E.; Mshiu, E.; Samuelsson, G. Inventory of plants used in traditional medicine in Tanzania. I. Plants of the families acanthaceae-cucurbitaceae. *J. Ethnopharmacol.* **1982**, *6*, 29–60.
67. Augustino, S.; Gillah, P.R. Medicinal plants in urban districts of Tanzania: Plants, gender roles and sustainable use. *Int. For. Rev.* **2005**, *7*, 44–58.
68. Watt, J.M.; Breyer-Brandwijk, M.G. The Medicinal and Poisonous Plants of Southern and Eastern Africa being an Account of their Medicinal and other Uses, Chemical Composition, Pharmacological Effects and Toxicology in Man and Animal. In *The Medicinal and Poisonous Plants of Southern and Eastern Africa Being an Account of Their Medicinal and Other Uses, Chemical Composition, Pharmacological Effects and Toxicology in Man and Animal*; E. & S. Livingstone Ltd.: Edinburgh, UK, 1962.
69. Chhabra, S.; Mahunnah, R.; Mshiu, E. Plants used in traditional medicine in Eastern Tanzania. II. Angiosperms (Capparidaceae to Ebenaceae). *J. Ethnopharmacol.* **1989**, *25*, 339–359.
70. Kisangau, D.P.; Lyaruu, H.V.; Hosea, K.M.; Joseph, C.C. Use of traditional medicines in the management of HIV/AIDS opportunistic infections in Tanzania: A case in the Bukoba rural district. *J. Ethnobiol. Ethnomed.* **2007**, *3*, 29.
71. Chhabra, S.; Mahunnah, B.; Mshiu, E. Plants used in traditional medicine in Eastern Tanzania. I. Pteridophytes and Angiosperms (Acanthaceae to Canellaceae). *J. Ethnopharmacol.* **1987**, *21*, 253–277.
72. Kokwaro, J.O. *Medicinal Plants of East Africa*; East African Literature Bureau: Nairobi, Kenya, 1976.
73. Mbunde, M.V.; Innocent, E.; Mabiki, F.; Andersson, P.G. Ethnobotanical survey and toxicity evaluation of medicinal plants used for fungal remedy in the Southern Highlands of Tanzania. *J. Intercult. Ethnopharmacol.* **2017**, *6*, 84–96.
74. Chhabra, S.; Mahunnah, R.; Mshiu, E. Plants used in traditional medicine in Eastern Tanzania. III. Angiosperms (Euphorbiaceae to Menispermaceae). *J. Ethnopharmacol.* **1990**, *28*, 255–283.
75. Maregesi, S.M.; Ngassapa, O.D.; Pieters, L.; Vlietinck, A.J. Ethnopharmacological survey of the Bunda district, Tanzania: Plants used to treat infectious diseases. *J. Ethnopharmacol.* **2007**, *113*, 457–470.
76. Sandberg, F. Étude sur les plantes médicinales et toxiques d'Afrique équatoriale. *Cah. Maboké* **1965**, *3.1*, 5–49.
77. Haerdi, F. The Indigenous Medicinal Plants of Ulanga District, Tanzania, East Africa. *Acta Trop.* **1964, 8,** 1–278.
78. Otieno, J.; Magadula, J.; Kakudidi, E.; Kirimhuzya, C.; Orodho, J.; Okemo, P. Use of ethnobotanical criteria for conservation assessment of plants used for respiratory diseases in Lake Victoria region, Tanzania. *Int. J. Biodivers. Conserv.* **2011**, *3*, 610–617.
79. Khan, M.; Nkunya, M. Antimicrobial activity of Tanzanian traditional medicinal plants. In *Ministry of Health, Proceedings of an International Conference on Traditional Medicinal Plants, Arusha, Tanzania, 18-23 Feb 1990*; Ministry of Health: Dodoma, Tanzania, 1990.
80. Orodho, J.; Okemo, P.; Tabuti, J.; Otieno, N.; Magadula, J.; Kirimuhuzya, C. Indigenous knowledge of communities around Lake Victoria Basin regarding treatment and management of tuberculosis using medicinal plants. *Int. J. Med. Med. Sci.* **2014**, *6*, 16–23.
81. Chhabra, S.C.; Mahunnah, R.; Mshiu, E.N. Plants used in traditional medicine in Eastern Tanzania. VI. Angiosperms (Sapotaceae to Zingiberaceae). *J. Ethnopharmacol.* **1993**, *39*, 83–103.
82. Burkill, H. *The Useful Plants of West Africa*; Royal Botanical Gardens, Kew: London, UK, 1985; p. 1.
83. Toyang, N.J.; Verpoorte, R. A review of the medicinal potentials of plants of the genus *Vernonia* (Asteraceae). *J. Ethnopharmacol.* **2013**, *146*, 681–723.
84. Hedberg, I.; Hedberg, O.; Madat, P.J.; Mshigeni, K.E.; Mshiu, E.; Samuelsson, G. Inventory of plants used in traditional medicine in Tanzania. II. Plants of the families Dilleniaceae—Opiliaceae. *J. Ethnopharmacol.* **1983**, *9*, 105–127.

85. Chhabra, S.; Mahunnah, R.; Mshiu, E. Plants used in traditional medicine in Eastern Tanzania. IV. Angiosperms (Mimosaceae to Papilionaceae). *J. Ethnopharmacol.* **1990**, *29*, 295–323.
86. Neuwinger, H. *Afrikanische Arzneipflanzen und Jagdgifte: Chemie, Pharmakologie, Toxikologie, Aloeaceae*; Wissenschaftliche Verlagsgesellschaft mbH: Stuttgart, Germany, 1998; pp. 1–7.
87. De Boer, H.J.; Kool, A.; Broberg, A.; Mziray, W.R.; Hedberg, I.; Levenfors, J.J. Anti-fungal and anti-bacterial activity of some herbal remedies from Tanzania. *J. Ethnopharmacol.* **2005**, *96*, 461–469.
88. Kerharo, J.; Adam, J.-G. *La Pharmacopée Sénégalaise Traditionnelle: Plantes Médicinales et Toxiques*; Vigot: Paris, France, 1974.

89. Hedberg, I.; Hedberg, O.; Madati, P.J.; Mshigeni, K.E.; Mshiu, E.; Samuelsson, G. Inventory of plants used in traditional medicine in Tanzania. Part III. Plants of the families Papilionaceaevitaceae. *J. Ethnopharmacol.* **1983**, *9*, 237–260.
90. Prinsloo, G.; Marokane, C.K.; Street, R.A. Anti-HIV activity of southern African plants: Current developments, phytochemistry and future research. *J. Ethnopharmacol.* **2018**, *210*, 133–155.
91. Nadkami, K.; Nadkarni, A. *Indian Materia Medica: With Ayurvedic, Unani-Tibbi, Siddha, Allopathic, Homeopathic, Naturopathic and Home Remedies, Appendices and Indexes*; Popular Prakashan Private Ltd: Bombay, India, 1976.
92. Chopra, R.N.; Nayar, S.L.; Chopra, I.C. *Glossary of Indian Medicinal Plants*; Council of Scientific & Industrial Research: New Delhi, India, 1956.
93. Chhabra, S.C.; Mahunnah, R. Plants used in traditional medicine by Hayas of the Kagera region, Tanzania. *Econ. Bot.* **1994**, *48*, 121–129.
94. Maroyi, A. Lippia javanica (Burm.f.) Spreng.: Traditional and Commercial Uses and Phytochemical and Pharmacological Significance in the African and Indian Subcontinent. *Evid. Based Complement. Altern. Med.* **2017**, *2017*, 34.
95. Chhabra, S.; Mahunnah, R.; Mshiu, E. Plants used in traditional medicine in Eastern Tanzania. V. Angiosperms (Passifloraceae to Sapindaceae). *J. Ethnopharmacol.* **1991**, *33*, 143–157.
96. Boily, Y.; Van Puyvelde, L. Screening of medicinal plants of Rwanda (Central Africa) for antimicrobial activity. *J. Ethnopharmacol.* **1986**, *16*, 1–13.
97. Samy, R.P.; Ignacimuthu, S.; Sen, A. Screening of 34 Indian medicinal plants for antibacterial properties. *J. Ethnopharmacol.* **1998**, *62*, 173–181.
98. Panda, S.K. Ethno-medicinal uses and screening of plants for antibacterial activity from Similipal Biosphere Reserve, Odisha, India. *J. Ethnopharmacol.* **2014**, *151*, 158–175.
99. Shendkar, C.; Chandrachood, P.; Lavate, S.; Jagtap, S.; Deshpande, N. Antibacterial screening of *Achyranthes aspera* Linn. root extracts. *Ann. Biol. Res.* **2012**, *3*, 2202–2204.
100. Naidu, P.L.; Kumar, K.K.; Kumar, C.M.; Gunesh, G.; Rao, M.N. Antimicrobial activity of *Achyranthes aspera*. *Biosci. Biotechnol. Res. Asia* **2006**, *3*, 171–174.
101. Newton, S.M.; Lau, C.; Gurcha, S.S.; Besra, G.S.; Wright, C.W. The evaluation of forty-three plant species for in vitro antimycobacterial activities; isolation of active constituents from *Psoralea corylifolia* and *Sanguinaria canadensis*. *J. Ethnopharmacol.* **2002**, *79*, 57–67.
102. Huish, R.D.; Huish, K.H.; Walker, T.J.; Shrestha, G. Traditional Tongan treatments for infections: Bioassays and ethnobotanical leads for activity. *J. Med. Plant Res.* **2014**, *8*, 1215–1222.
103. Valsaraj, R.; Pushpangadan, P.; Smitt, U.; Adsersen, A.; Nyman, U. Antimicrobial screening of selected medicinal plants from India. *J. Ethnopharmacol.* **1997**, *58*, 75–83.
104. Elmi, A.S.; Svendsen, A.B.; Sheffer, J.J.; Verpoorte, R. Screening of some Somalian medicinal plants for antimicrobial activity. *J. Ethnopharmacol.* **1986**, *17*, 283–288.
105. Desta, B. Ethiopian traditional herbal drugs. Part II: Antimicrobial activity of 63 medicinal plants. *J. Ethnopharmacol.* **1993**, *39*, 129–139.
106. Lall, N.; Meyer, J. In vitro inhibition of drug-resistant and drug-sensitive strains of *Mycobacterium tuberculosis* by ethnobotanically selected South African plants. *J. Ethnopharmacol.* **1999**, *66*, 347–354.

107. Bezerra, J.W.A.; Costa, A.R.; de Freitas, M.A.; Rodrigues, F.C.; de Souza, M.A.; da Silva, A.R.P.; Dos Santos, A.T.L.; Linhares, K.V.; Coutinho, H.D.M.; de Lima Silva, J.R. Chemical composition, antimicrobial, modulator and antioxidant activity of essential oil of *Dysphania ambrosioides* (L.) Mosyakin & Clemants. *Comp. Immunol. Microbiol. Infect. Dis.* **2019**, *65*, 58–64.

108. Haule, E.E.; Moshi, M.J.; Nondo, R.S.; Mwangomo, D.T.; Mahunnah, R.L. A study of antimicrobial activity, acute toxicity and cytoprotective effect of a polyherbal extract in a rat ethanol-HCl gastric ulcer model. *BMC Res. Notes* **2012**, *5*, 546.
109. Maregesi, S.M.; Pieters, L.; Ngassapa, O.D.; Apers, S.; Vingerhoets, R.; Cos, P.; Berghe, D.A.; Vlietinck, A.J. Screening of some Tanzanian medicinal plants from Bunda district for antibacterial, antifungal and antiviral activities. *J. Ethnopharmacol.* **2008**, *119*, 58–66.
110. Chimponda, T.; Mukanganyama, S. Antimycobacterial activities of selected medicinal plants from Zimbabwe against *Mycobacterium aurum* and *Corynebacterium glutamicum*. *Trop. Biomed.* **2010**, *27*, 595–610.
111. Njeru, S.N.; Obonyo, M.A. Potency of extracts of selected plant species from Mbeere, Embu County-Kenya against *Mycobacterium tuberculosis*. *J. Med. Plant Res.* **2016**, *10*, 149–157.
112. Tsouh Fokou, P.V.; Kissi-Twum, A.A.; Yeboah-Manu, D.; Appiah-Opong, R.; Addo, P.; Tchokouaha Yamthe, L.R.; Ngoutane Mfopa, A.; Fekam Boyom, F.; Nyarko, A.K. In vitro activity of selected West African medicinal plants against *Mycobacterium ulcerans* disease. *Molecules* **2016**, *21*, 445.
113. Kabongo-Kayoka, P.N.; Eloff, J.N.; Obi, C.L.; McGaw, L.J. Antimycobacterial activity and low cytotoxicity of leaf extracts of some African Anacardiaceae tree species. *Phytother. Res.* **2016**, *30*, 2001–2011.
114. Hostettmann–Kaldas, M.; Nakanishi, K. Moronic Acid, a Simple Triterpenoid Keto Acid with Antimicrobial Activity Isolated from *Ozoroa mucronata*. *Planta Med.* **1979**, *37*, 358–360.
115. Kisangau, D.P.; Hosea, K.M.; Joseph, C.C.; Lyaruu, H.V. In vitro antimicrobial assay of plants used in traditional medicine in Bukoba Rural district, Tanzania. *Afr. J. Tradit. Complement. Altern. Med.* **2007**, *4*, 510–523.
116. Kareru, P.; Gachanja, A.; Keriko, J.; Kenji, G. Antimicrobial activity of some medicinal plants used by herbalists in eastern province, Kenya. *Afr. J. Tradit. Complement. Altern. Med.* **2008**, *5*, 51–55.

117. Lino, A.; Deogracious, O. The in-vitro antibacterial activity of *Annona senegalensis*, *Securidacca longipendiculata* and *Steganotaenia araliacea*—Ugandan medicinal plants. *Afr. Health Sci.* **2006**, *6*, 31–35.
118. Khan, M. Antibacterial activity of some Tanzanian medicinal plants. *Pharm. Biol.* **2001**, *39*, 206–212.
119. More, G.K.; Tshikalange, T.E.; Lall, N.; Meyer, J.J.M.; Botha, F.S.; Hussein, A.A. Antimicrobial activity of medicinal plants against oral microorganisms. *S. Afr. J. Bot.* **2007**, *73*, 332.
120. Tshikalange, T.; Meyer, J.; Hussein, A. Antimicrobial activity, toxicity and the isolation of a bioactive compound from plants used to treat sexually transmitted diseases. *J. Ethnopharmacol.* **2005**, *96*, 515–519.
121. McGaw, L.; Jäger, A.; Van Staden, J. Antibacterial, anthelmintic and anti-amoebic activity in South African medicinal plants. *J. Ethnopharmacol.* **2000**, *72*, 247–263.
122. Erasto, P.; Lubschagne, A.; Mbwambo, Z.H.; Nondo, R.S.; Lall, N. Antimycobacterial, antioxidant activity and toxicity of extracts from the roots of *Rauvolfia vomitoria* and *R. caffra*. *Spatula DD* **2011**, *1*, 73–80.
123. Steenkamp, V.; Mathivha, E.; Gouws, M.; Van Rensburg, C. Studies on antibacterial, antioxidant and fibroblast growth stimulation of wound healing remedies from South Africa. *J. Ethnopharmacol.* **2004**, *95*, 353–357.
124. Teka, A.; Rondevaldova, J.; Asfaw, Z.; Demissew, S.; Van Damme, P.; Kokoska, L.; Vanhove, W. In vitro antimicrobial activity of plants used in traditional medicine in Gurage and Silti Zones, south central Ethiopia. *BMC Complement. Altern. Med.* **2015**, *15*, 1–7.
125. Madikizela, B.; Ndhlala, A.R.; Finnie, J.F.; Van Staden, J. Antimycobacterial, anti-inflammatory and genotoxicity evaluation of plants used for the treatment of tuberculosis and related symptoms in South Africa. *J. Ethnopharmacol.* **2014**, *153*, 386–391.
126. Mmushi, T.; Masoko, P.; Mde, L.; Mokgotho, M.; Mampuru, L.; Howard, R. Antimycobacterial evaluation of fifteen medicinal plants in South Africa. *Afr. J. Tradit. Complement. Altern. Med.* **2010**, *7*.
127. Grace, O.; Light, M.; Lindsey, K.; Mulholland, D.; Van Staden, J.; Jager, A.; Eloff, J. Antibacterial activity and isolation of active compounds from fruit of the traditional African medicinal tree *Kigelia africana*. *S. Afr. J. Bot.* **2002**, *68*, 220–222.
128. Viol, D.I.; Chagonda, L.S.; Moyo, S.R.; Mericli, A.H. Antibacterial, antifungal and phytochemical activities of some medicinal plants from Zimbabwe. *J. Biol. Act. Prod. Nat.* **2013**, *3*, 273–284.
129. Nciki, S.; Vuuren, S.; van Eyk, A.; de Wet, H. Plants used to treat skin diseases in northern Maputaland, South Africa: Antimicrobial activity and in vitro permeability studies. *Pharm. Biol.* **2016**, *54*, 2420–2436.
130. Agyare, C.; Dwobeng, A.S.; Agyepong, N.; Boakye, Y.D.; Mensah, K.B.; Ayande, P.G.; Adarkwa-Yiadom, M. Antimicrobial, antioxidant, and wound healing properties of *Kigelia africana* (Lam.) Beneth. and *Strophanthus hispidus* DC. *Adv. Pharmacol. Sci.* **2013**, *2013*, 692613.

131. Sidjui, L.; Toghueo, R.M.K.; Zeuko'o, E.M.; Mbouna, C.D.J.; Mahiou-Leddet, V.; Herbette, G.; Folefoc, G. Antibacterial activity of the crude extracts, fractions and compounds from the stem barks of *Jacaranda mimosifolia* and *Kigelia africana* (Bignoniaceae). *Pharmacologia* **2016**, *7*, 22–31.
132. Arkhipov, A.; Sirdaarta, J.; Rayan, P.; McDonnell, P.; Cock, I. An examination of the antibacterial, antifungal, anti-Giardial and anticancer properties of *Kigelia africana* fruit extracts. *Pharmacogn. Commun.* **2014**, *4*, 62-76.
133. Fomogne-Fodjo, M.; Van Vuuren, S.; Ndinteh, D.; Krause, R.; Olivier, D. Antibacterial activities of plants from Central Africa used traditionally by the Bakola pygmies for treating respiratory and tuberculosis-related symptoms. *J. Ethnopharmacol.* **2014**, *155*, 123–131.
134. Shai, L.; McGaw, L.; Masoko, P.; Eloff, J. Antifungal and antibacterial activity of seven traditionally used South African plant species active against *Candida albicans*. *S. Afr. J. Bot.* **2008**, *74*, 677–684.
135. Eldeen, I.M.; Van Heerden, F.R.; Van Staden, J. Isolation and biological activities of termilignan B and arjunic acid from *Terminalia sericea* roots. *Planta Med.* **2008**, *74*, 411–413.
136. Karou, S.D.; Tchacondo, T.; Ouattara, L.; Anani, K.; Savadogo, A.; Agbonon, A.; Attaia, M.B.; de Souza, C.; Sakly, M.; Simpore, J. Antimicrobial, antiplasmodial, haemolytic and antioxidant activities of crude extracts from three selected Togolese medicinal plants. *Asian Pac. J. Trop. Med.* **2011**, *4*, 808–813.
137. Van Vuuren, S.; Viljoen, A. The in vitro antimicrobial activity of toothbrush sticks used in Ethiopia. *S. Afr. J. Bot.* **2006**, *72*, 646–648.
138. Mishra, M.P.; Padhy, R.N. In vitro antibacterial efficacy of 21 Indian timber-yielding plants against multidrug-resistant bacteria causing urinary tract infection. *Osong Public Health Res. Perspect.* **2013**, *4*, 347–357.
139. Geyid, A.; Abebe, D.; Debella, A.; Makonnen, Z.; Aberra, F.; Teka, F.; Kebede, T.; Urga, K.; Yersaw, K.; Biza, T. Screening of some medicinal plants of Ethiopia for their anti-microbial properties and chemical profiles. *J. Ethnopharmacol.* **2005**, *97*, 421–427.
140. Tesfamaryam, G.; Tsegaye, B.; Eguale, T.; Wubete, A. In vitro screening of antibacterial activities of selected athiopian medicinal plants. *Int. J. Microbiol. Res.* **2015**, *6*, 27–33.
141. Fentahun, M.; Ayele, Y.; Amsalu, N.; Alemayehu, A.; Amsalu, G. Antibacterial evaluation and phytochemical analysis of selected medicinal plants against some pathogenic enteric bacteria in Gozamin District, Ethiopia. *J. Pharmacovigil.* **2017**, *5*, 1–6.
142. Saini, N.; Singhal, M.; Srivastava, B.; Sachdeva, K.; Singh, C. Anti-microbial activity of *Tecomaria capensis* leaves extract. *In Vitro* **2011**, *7*, 24.
143. Akhalwaya, S.; Van Vuuren, S.; Patel, M. An in vitro investigation of indigenous South African medicinal plants used to treat oral infections. *J. Ethnopharmacol.* **2018**, *210*, 359–371.
144. Maregesi, S.; Nyamwisenda, N.; Mwangomo, D.; Kidukuli, A. In vitro antimicrobial activity and determination of essential metal and ash value contents of *Trichodesma zeylanicum*. *Int. J. Res. Pharmacol. Pharmacerapeutic* **2013**, *2*, 417–424.
145. Dzoyem, J.P.; Aro, A.O.; McGaw, L.J.; Eloff, J.N. Antimycobacterial activity against different pathogens and selectivity index of fourteen medicinal plants used in southern Africa to treat tuberculosis and respiratory ailments. *S. Afr. J. Bot.* **2016**, *102*, 70–74.

146. Khumalo, G.; Sadgrove, N.; Van Vuuren, S.; Van Wyk, B.-E. Antimicrobial activity of volatile and non-volatile isolated compounds and extracts from the bark and leaves of *Warburgia salutaris* (Canellaceae) against skin and respiratory pathogens. *S. Afr. J. Bot.* **2019**, *122*, 547–550.
147. Kuglerova, M.; Tesarova, H.; Grade, J.T.; Halamova, K.; Wanyana-Maganyi, O.; Van Damme, P.; Kokoska, L. Antimicrobial and antioxidative effects of Ugandan medicinal barks. *Afr. J. Biotechnol* **2011**, *10*, 3628-3632.
148. Steenkamp, V.; Fernandes, A.C.; Jansen van Rensburg, C.E. *Antibacterial Activity of Venda Medicinal Plants*; University of Pretoria: Pretoria, South Africa, 2007.
149. Mbwambo, Z.; Erasto, P.; Innocent, E.; Masimba, P. Antimicrobial and cytotoxic activities of fresh leaf extracts of *Warburgia ugandensis*. *Tanzan. J. Health Res.* **2009**, *11*, 45205.
150. Olila, D.; Opuda-Asibo, J. Antibacterial and antifungal activities of extracts of *Zanthoxylum chalybeum* and *Warburgia ugandensis*, Ugandan medicinal plants. *Afr. Health Sci.* **2001**, *1*, 66–72.
151. Rout, J.; Sajem, A.L.; Nath, M.; Sengupta, M. Antibacterial efficacy of bark extracts of an ethnomedicinal plant *Trema orientalis* Blume. *Curr. Trends Biotechnol. Pharm.* **2012**, *6*, 464–471.
152. Omwenga, E.; Okemo, P.; Mbugua, P.; Ogol, C. *Ethnobotanical Survey and Antimicrobial Evaluation of Medicinal Plants Used by the Samburu Community (Kenya) for Treatment of Diarrhorea*; Kisii University: Kisii, Kenya, 2009; Volume

4, pp. 165–176.
153. Mariita, R.M.; Orodho, J.A.; Okemo, P.O.; Kirimuhuzya, C.; Otieno, J.N.; Magadula, J.J. Methanolic extracts of *Aloe secundiflora* Engl. inhibits in vitro growth of tuberculosis and diarrhea-causing bacteria. *Pharmacogn. Res.* **2011**, *3*, 95.
154. Anywar, G.; Oryem-Origa, H.; Kamatenesi-Mugisha, M. Antibacterial and antifungal properties of some wild nutraceutical plant species from Nebbi District, Uganda. *Br. J. Pharm. Res.* **2014**, *4*, 1753.
155. Ramalivhana, J.; Moyo, S.; Obi, C. The possible role of medicinal plants in tackling resistant microbial pathogens in Limpopo Province, South Africa. *J. Med. Plant Res.* **2010**, *4*, 999–1002.
156. Luo, X.; Pires, D.; Aínsa, J.A.; Gracia, B.; Mulhovo, S.; Duarte, A.; Anes, E.; Ferreira, M.-J.U. Antimycobacterial evaluation and preliminary phytochemical investigation of selected medicinal plants traditionally used in Mozambique. *J. Ethnopharmacol.* **2011**, *137*, 114–120.
157. Oshomoh, E.; Idu, M. Antimicrobial activity of ethanol and aqueous extracts of *Parinari curatellifolia* (stem) on dental caries causing microbes. *Int. J. Pharm. Sci.* **2012**, *3*, 2113.
158. Peni, I.; Elinge, C.; Yusuf, H.; Itodo, A.; Agaie, B.; Mbongo, A.; Chogo, E. Phytochemical screening and antibacterial activity of *Parinari curatellifolia* stem extract. *J. Med. Plant Res.* **2010**, *4*, 2099–2102.
159. More, G.; Tshikalange, T.E.; Lall, N.; Botha, F.; Meyer, J.J.M. Antimicrobial activity of medicinal plants against oral microorganisms. *J. Ethnopharmacol.* **2008**, *119*, 473–477.
160. Lall, N.; de Canha, M.; Reid, A.; Oosthuizen, C.; Langhansova, L.; Mahore, J.; Winterboer, S.; Hamilton, C.; Kumar, V.; Gasa, N.; et al. Antibacterial and Anticancer Activity of Ethnomedicinal Plants Used in the Jongilanga Community, Mpumalanga. *Int. J. Pharmacogn. Phytochem. Res.* **2018**, *10*, 25–37.
161. Fyhrquist, P.; Mwasumbi, L.; Hæggström, C.-A.; Vuorela, H.; Hiltunen, R.; Vuorela, P. Ethnobotanical and antimicrobial investigation on some species of *Terminalia and Combretum* (Combretaceae) growing in Tanzania. *J. Ethnopharmacol.* **2002**, *79*, 169–177.
162. Nyambuya, T.; Mautsa, R.; Mukanganyama, S. Alkaloid extracts from *Combretum zeyheri* inhibit the growth of *Mycobacterium smegmatis*. *BMC Complement. Altern. Med.* **2017**, *17*, 1–11.
163. Masengu, C.; Zimba, F.; Mangoyi, R.; Mukanganyama, S. Inhibitory activity of *Combretum zeyheri* and its S9 metabolites against *Escherichia coli*, *Bacillus subtilis* and *Candida albicans*. *J. Microbial. Biochem. Technol.* **2014**, *6*, 228–235.
164.Wagate, C.G.; Mbaria, J.M.; Gakuya, D.W.; Nanyingi, M.O.; Kareru, P.; Njuguna, A.; Gitahi, N.; Macharia, J.K.; Njonge, F.K. Screening of some Kenyan medicinal plants for antibacterial activity. *Phytother. Res.* **2010**, *24*, 150–153.
165. Mohamad, S.; Zin, N.M.; Wahab, H.A.; Ibrahim, P.; Sulaiman, S.F.; Zahariluddin, A.S.M.; Noor, S.S.M. Antituberculosis potential of some ethnobotanically selected Malaysian plants. *J. Ethnopharmacol.* **2011**, *133*, 1021–1026.
166. Chah, K.; Eze, C.; Emuelosi, C.; Esimone, C. Antibacterial and wound healing properties of methanolic extracts of some Nigerian medicinal plants. *J. Ethnopharmacol.* **2006**, *104*, 164–167.
167. Chander, M.P.; Vijayachari, P. In vitro antimicrobial and anti-oxidant potentials of selected medicinal plants used by the indigenous tribes of Andaman and Nicobar Islands, India. *Bangladesh J. Pharmacol.* **2016**, *11*, 330–332.
168. Akhtar, N.; Mirza, B. Phytochemical analysis and comprehensive evaluation of antimicrobial and antioxidant properties of 61 medicinal plant species. *Arab. J. Chem.* **2018**, *11*, 1223–1235.
169. Magadula, J.J.; Otieno, J.N.; Nondo, R.S.; Kirimuhuzya, C.; Kadukuli, E.; Orodho, J.A.; Okemo, P. Anti-mycobacterial and toxicity activities of some priority medicinal plants from lake Victoria basin, Tanzania. *Eur. J. Med. Plants* **2012**125-131.
170. Dickson, R.; Houghton, P.; Hylands, P.; Gibbons, S. Antimicrobial, resistance-modifying effects, antioxidant and free radical scavenging activities of *Mezoneuron benthamianum* Baill., *Securinega virosa* Roxb. &Wlld. and *Microglossa pyrifolia* Lam. *Phytother. Res.* **2006**, *20*, 41–45.
171. Magak, S.K.; Njoroge, G.N. Self-reported Hand Hygiene Practices among Traditional Health Care Workers (Herbalists) In Bungoma, Kenya. *Trop Med. Health* **2010**, *38*, 133–137.
172. Sonibare, M.A.; Aremu, O.T.; Okorie, P.N. Antioxidant and antimicrobial activities of solvent fractions of *Vernonia cinerea* (L.) Less leaf extract. *Afr. Health Sci.* **2016**, *16*, 629–639.
173. Chen, C.-P.; Lin, C.-C.; Tsuneo, N. Screening of Taiwanese crude drugs for antibacterial activity against *Streptococcus mutans*. *J. Ethnopharmacol.* **1989**, *27*, 285–295.

174. Kumar, S.; Bagchi, G.; Darokar, M. Antibacterial activity observed in the seeds of some coprophilous plants. *Int. J. Pharmacogn.* **1997**, *35*, 179–184.
175. Latha, L.Y., Jr.; Darah, I.; Sasidharan, S.; Jain, K. Antimicrobial activity of *Emilia sonchifolia* DC., *Tridax procumbens* L. and *Vernonia cinerea* L. of Asteracea Family: Potential as Food Preservatives. *Malays. J. Nutr.* **2009**, *15*, 223–231.
176. Danish Rizvi, S.; Biswas, D.; Arif, J.; Zeeshan, M. In-vitro antibacterial and antioxidant potential of leaf and flower extracts of *Vernonia cinerea* and their phytochemical constituents. *Int. J. Pharm. Sci. Rev. Res.* **2011**, *9*, 164–169.
177. Mahida, Y.; Mohan, J. Screening of Indian plant extracts for antibacterial activity. *Pharm. Biol.* **2006**, *44*, 627–631.
178. Izah, S.C.; Ilerhunmwuwa, I.A.; Ibibo, T.D.; Udumo, J.J. Activities of express extracts of Costus afer Ker-Gawl. [Family COSTACEAE] against selected bacterial isolates. *J. Pharm Phytopharm Res.* **2019**, *9*, 39–44.
179. Dahiya, P.; Purkayastha, S. Phytochemical screening and antimicrobial activity of some medicinal plants against multi-drug resistant bacteria from clinical isolates. *Indian J. Pharm. Sci.* **2012**, *74*, 443.
180. Kardong, D.; Upadhyaya, S.; Saikia, L. Screening of phytochemicals, antioxidant and antibacterial activity of crude extract of *Pteridium aquilinum* Kuhn. *J. Pharm. Res.* **2013**, *6*, 179–182.
181. Esimone, C.; Nworu, C.; Onuigbo, E.; Omeje, J.; Nsirim, K.; Ogbu, J.; Ngwu, M.; Chah, K. Anti-mycobacterial activity of root and leaf extracts of *Anthocleista djalonensis* (Loganiaceae) and *Diospyros mespiliformis* (Ebenaceae). *Int. J. Green Pharm.* **2009**, *3*, 201.
182. Mothana, R.A.; Lindequist, U.; Gruenert, R.; Bednarski, P.J. Studies of the in vitro anticancer, antimicrobial and antioxidant potentials of selected Yemeni medicinal plants from the island Soqotra. *BMC Complement. Altern. Med.* **2009**, *9*, 1–11.
183. Hamill, F.; Apio, S.; Mubiru, N.; Bukenya-Ziraba, R.; Mosango, M.; Maganyi, O.; Soejarto, D. Traditional herbal drugs of Southern Uganda, II: Literature analysis and antimicrobial assays. *J. Ethnopharmacol.* **2003**, *84*, 57–78.
184. Villaseñor, I.M.; Cariño, F.A. Antimicrobial activity of new phorbins from *Jatropha curcas* Linn. (Euphorbiaceae) leaves. *Z. Nat. C* **2011**, *66*, 441–446.
185. Rojas, R.; Caviedes, L.; Aponte, J.C.; Vaisberg, A.J.; Lewis, W.H.; Lamas, G.; Sarasara, C.; Gilman, R.H.; Hammond, G.B. Aegicerin, the First Oleanane Triterpene with Wide-Ranging Antimycobacterial Activity, Isolated from *Clavija p rocera*. *J. Nat. Prod.* **2006**, *69*, 845–846.
186. Gangoué-Piéboji, J.; Eze, N.; Djintchui, A.N.; Ngameni, B.; Tsabang, N.; Pegnyemb, D.E.; Biyiti, L.; Ngassam, P.; Koulla-Shiro, S.; Galleni, M. The in-vitro antimicrobial activity of some medicinal plants against β-lactam-resistant bacteria. *J. Infect. Dev. Ctries.* **2009**, *3*, 671–680.
187. Chukwujekwu, J.C.; Van Staden, J.; Smith, P.; Meyer, J. Antibacterial, anti-inflammatory and antimalarial activities of some Nigerian medicinal plants. *S. Afr. J. Bot.* **2005**, *71*, 316–325.
188. Mpinda, C.; Meyer, D.; Lyantagaye, S. In vitro antimicrobial activity of extracts of some plant species used in the management of HIV/AIDS in Tanzania. *Tanz. J. Sci.* **2018**, *44*, 179–190.
189. Merveille, T.O.; Denis, Z.; Abdel, N.J.; Ursula, A.A.; Adie, A.F.A.M.; Smith, B.B.; Zipoh, N.A.; Joseph, Y.N.; Peter, N.F.; Veronique, P.B. Antimicrobial Activities of Some Medicinal Plants Against Multiresistants Microorganisms Causing Urogenital Tract Infections in Cameroon. *JDMP* **2017**, *3*, 33–41.
190. Ighodaro, O.; Akinloye, O.; Popoola, J.; Adebodun, S. Phytochemical distribution and antimicrobial sensitivity of *Sapium ellipticum* (Hochst) pax leaf extracts. *Eur. J. Med. Plants* **2018**, *25*, 1–11.
191. Nguta, J.M.; Kiraithe, M.N. *In vitro* antimicrobial activity of aqueous extracts of *Ocimum suave* Willd., *Plectranthus barbatus* andrews and *Zanthoxylum chalybeum* Engl. against selected pathogenic bacteria. *Biomed. Biotechnol. Res.* **2019**, *3*, 30.
192. Van Puyvelde, L.; Ntawukiliyayo, J.; Portaels, F.; Hakizamungu, E. In vitro inhibition of mycobacteria by Rwandese medicinal plants. *Phytother. Res.* **1994**, *8*, 65–69.
193. Rojas, A.; Hernandez, L.; Pereda-Miranda, R.; Mata, R. Screening for antimicrobial activity of crude drug extracts and pure natural products from Mexican medicinal plants. *J. Ethnopharmacol.* **1992**, *35*, 275–283.
194. Van Puyvelde, L.; Nyirankuliza, S.; Panebianco, R.; Boily, Y.; Geizer, I.; Sebikali, B.; De Kimpe, N.; Schamp, N. Active principles of *Tetradenia riparia*. I. Antimicrobial activity of 8 (14), 15-sandaracopimaradiene-7α, 18-diol. *J. Ethnopharmacol.* **1986**, *17*, 269–275.
195. Chogo, J.; Crank, G. Chemical composition and biological activity of the Tanzanian plant Ocimum suave. *J. Nat. Prod.* **1981**, *44*, 308–311.
196. Mariita, R.M.; Okemo, P.; Orodho, J.A.; Kirimuhuzya, C.; Otieno, J.N.; Joseph, M. Efficacy of 13 medicinal plants used by indigenous communities around Lake Victoria, Kenya, against tuberculosis, diarrhoea causing bacteria and

Candida albicans. *Int. J. Pharm.* **2010**, *2*, 771–791.
197. Ibekwe, N.N.; Nvau, J.B.; Oladosu, P.O.; Usman, A.M.; Ibrahim, K.; Boshoff, H.I.; Dowd, C.S.; Orisadipe, A.T.; Aiyelaagbe, O.; Adesomoju, A.A. In collaboration with 73 visited herbalists. Some Nigerian antituberculosis ethnomedicines: A preliminary efficacy assessment. *J. Ethnopharmacol.* **2014**, *155*, 524–532.
198. Zore, G.; Awad, V.; Thakre, A.; Halde, U.; Meshram, N.; Surwase, B.; Karuppayil, S.M. Activity-directed-fractionation and isolation of four antibacterial compounds from *Abrus precatorius* L., roots. *Nat. Prod. Res.* **2007**, *21*, 838–845.
199. Idris, M.; Nkafamiya, I.; Akinterinwa, A.; Japari, J. Preliminary studies on some medicinal plants in Girei, Adamawa State of Nigeria. *Br. J. Pharm. Res.* **2015**, *6*, 203–213.
200. Lalitha, S.; Rajeshwaran, K.; Kumar, P.S.; Deepa, K.; Gowthami, K. In vivo screening of antibacterial activity of *Acacia mellifera* (BENTH) (Leguminosae) on human pathogenic bacteria. *Glob. J. Pharmacol.* **2010**, *4*, 148–150.
201. Oladosu, P.; Samuel, B.; Okhale, S.; Ibrahim, K.; Okogun, J. Antitubercular activity of the dried fruits of *Acacia nilotica* Linn Willd. *J. Phytomed. Ther.* **2007**, *12*, 76–79.
202. Kambizi, L.; Afolayan, A.J. An ethnobotanical study of plants used for the treatment of sexually transmitted diseases (njo hera) in Guruve District, Zimbabwe. *J. Ethnopharmacol.* **2001**, *77*, 5–9.

203. Constance, C.; Bagar, T.; Walter, C. Aqueous Extracts of *Pericopsis angolensis* and *Swartzia madagascariensis* with High Antimicrobial Activities against *Escherichia coli* O157, *Shigella spp.* and *Salmonella enterica* subsp. enterica (Serovar typhi). *Afr. J. Biotechnol.* **2019**, *18*, 831–844.
204. Mukanganyama, S.; Ntumy, A.N.; Maher, F.; Muzila, M.; Andrae-Marobela, K. Screening for anti-infective properties of selected medicinal plants from Botswana. *Afr. J. Plant Sci.* **2011**, *5*, 1–7.
205. McGaw, L.; Eloff, J.; Meyer, J. Screening of 16 poisonous plants for antibacterial, anthelmintic and cytotoxic activity in vitro. *S. Afr. J. Bot.* **2005**, *71*, 302–306.
206. Van Puyvelde, L.; Geiser, I.; Rwangabo, P.-C.; Sebikali, B. Rwandese herbal remedies used against gonorrhoea. *J. Ethnopharmacol.* **1983**, *8*, 279–286.
207. Zu, Y.-g.; Liu, X.-l.; Fu, Y.-j.; Wu, N.; Kong, Y.; Wink, M. Chemical composition of the SFE-CO_2 extracts from *Cajanus cajan* (L.) Huth and their antimicrobial activity *in vitro* and *in vivo*. *Phytomedicine* **2010**, *17*, 1095–1101.
208. Kong, Y.; Fu, Y.-J.; Zu, Y.-G.; Chang, F.-R.; Chen, Y.-H.; Liu, X.-L.; Stelten, J.; Schiebel, H.-M. Cajanuslactone, a new coumarin with anti-bacterial activity from pigeon pea [*Cajanus cajan* (L.) Millsp.] leaves. *Food Chem.* **2010**, *121*, 1150–1155.
209. Amugune, B.; Mwangi, J.; Thoithi, G.; Kibwage, I. In Vitro Screening of Ten Selected Traditionally Used Medicinal Plants in Vihiga County, Kenya for Antibacterial and Antifungal Activity. *Int. J. Med. Plants Nat. Prod.* **2017**, *3*, 37–44.
210. Sindambiwe, J.; Calomme, M.; Cos, P.; Totte, J.; Pieters, L.; Vlietinck, A.; Berghe, D.V. Screening of seven selected Rwandan medicinal plants for antimicrobial and antiviral activities. *J. Ethnopharmacol.* **1999**, *65*, 71–77.
211. Abdallah, M.; Warodi, F.; Gambo, R. Antibacterial Activity of Leaf Extract (*Cassia Mimosoides* Linn) on some Bacterial isolates from diarrhoeal samples of infant. *IJIR* **2017**, *3*.
212. Tekwu, E.M.; Askun, T.; Kuete, V.; Nkengfack, A.E.; Nyasse, B.; Etoa, F.-X.; Beng, V.P. Antibacterial activity of selected Cameroonian dietary spices ethno-medically used against strains of *Mycobacterium tuberculosis*. *J. Ethnopharmacol.* **2012**, *142*, 374–382.
213. Fotie, J.; Nkengfack, A.; Peter, M.; Heydenreich, M.; Fomum, Z. Chemical constituents of the ethyl acetate extracts of the stem bark and fruits of *Dichrostachys cinerea* and the roots of *Parkia bicolor*. *Bull. Chem. Soc. Ethiop.* **2004**, *18*.
214. Tamokou, J.d.D.; Chouna, J.R.; Fischer-Fodor, E.; Chereches, G.; Barbos, O.; Damian, G.; Benedec, D.; Duma, M.; Efouet, A.P.N.; Wabo, H.K. Anticancer and antimicrobial activities of some antioxidant-rich Cameroonian medicinal plants. *PLoS ONE* **2013**, *8*, e55880.
215. Fankam, A.G.; Kuete, V.; Voukeng, I.K.; Kuiate, J.R. Antibacterial activities of selected Cameroonian spices and their synergistic effects with antibiotics against multidrug-resistant phenotypes. *BMC Complement. Altern. Med.* **2011**, *11*, 1–11.
216. Tchinda, A.T.; Fuendjiep, V.; Mekonnen, Y.; Ngo, B.B.; Dagne, E. A bioactive diterpene from *Entada abyssinica*. *Nat. Prod. Commun.* **2007**, *2*, 9–12.
217. Fabry, W.; Okemo, P.O.; Ansorg, R. Antibacterial activity of East African medicinal plants. *J. Ethnopharmacol.* **1998**, *60*, 79–84.

218. Bunalema, L.; Kirimuhuzya, C.; Tabuti, J.; Waako, P.; Magadula, J.; Otieno, N.; Orodho, J.A.; Okemo, P. The efficacy of the crude root bark extracts of *Erythrina abyssinica* on rifampicin resistant *Mycobacterium tuberculosis*. *Afr. Health Sci.* **2011**, *11*, 587–593.
219. Dzoyem, J.P.; Kuete, V.; McGaw, L.J.; Eloff, J.N. The 15-lipoxygenase inhibitory, antioxidant, antimycobacterial activity and cytotoxicity of fourteen ethnomedicinally used African spices and culinary herbs. *J. Ethnopharmacol.* **2014**, *156*, 1–8.
220. Djeussi, D.E.; Noumedem, J.A.; Seukep, J.A.; Fankam, A.G.; Voukeng, I.K.; Tankeo, S.B.; Nkuete, A.H.; Kuete, V. Antibacterial activities of selected edible plants extracts against multidrug-resistant Gram-negative bacteria. *BMC Complement. Altern. Med.* **2013**, *13*, 1–8.
221. Voravuthikunchai, S.P.; Limsuwan, S. Medicinal plant extracts as anti–*Escherichia coli* O157: H7 agents and their effects on bacterial cell aggregation. *J. Food Prot.* **2006**, *69*, 2336–2341.
222. Tshikalange, T.E.; Modishane, D.C.; Tabit, F.T. Antimicrobial, antioxidant, and cytotoxicity properties of selected wild edible fruits of traditional medicinal plants. *J. Herbs Spices Med. Plants* **2017**, *23*, 68–76.
223. Isa, A.I.; Awouafack, M.D.; Dzoyem, J.P.; Aliyu, M.; Magaji, R.A.; Ayo, J.O.; Eloff, J.N. Some *Strychnos spinosa* (Loganiaceae) leaf extracts and fractions have good antimicrobial activities and low cytotoxicities. *BMC Complement. Altern. Med.* **2014**, *14*, 1–8.
224. Tabit, F.T.; Komolafe, N.T.; Tshikalange, T.E.; Nyila, M.A. Phytochemical constituents and antioxidant and antimicrobial activity of selected plants used traditionally as a source of food. *J. Med. Food* **2016**, *19*, 324–329.
225. Lagnika, L.; Amousa, M.; Adjovi, Y.; Sanni, A. Antifungal, antibacterial and antioxidant properties of *Adansonia digitata* and *Vitex doniana* from Bnin pharmacopeia. *J. Pharmacogn. Phytother.* **2012**, *4*, 44–52.
226. Mulaudzi, R.B.; Ndhlala, A.R.; Kulkarni, M.G.; Finnie, J.F.; Van Staden, J. Antimicrobial properties and phenolic contents of medicinal plants used by the Venda people for conditions related to venereal diseases. *J. Ethnopharmacol.* **2011**, *135*, 330–337.
227. Khan, M.N.; Ngassapa, O.; Matee, M.I. Antimicrobial activity of Tanzanian chewing sticks against oral pathogenic microbes. *Pharm. Biol.* **2000**, *38*, 235–240.
228. Begashaw, B.; Mishra, B.; Tsegaw, A.; Shewamene, Z. Methanol leaves extract *Hibiscus micranthus* Linn exhibited antibacterial and wound healing activities. *BMC Complement. Altern. Med.* **2017**, *17*, 1–11.
229.Woldeyes, S.; Adane, L.; Tariku, Y.; Muleta, D.; Begashaw, T. Evaluation of Antibacterial Activities of Compounds Isolated from *Sida rhombifolia*. *Nat. Prod. Chem. Res.* **2012**, *1*, 1-8
230. York, T.; Van Vuuren, S.; De Wet, H. An antimicrobial evaluation of plants used for the treatment of respiratory infections in rural Maputaland, KwaZulu-Natal, South Africa. *J. Ethnopharmacol.* **2012**, *144*, 118–127.
231. Ngoci, N.S.; Ramadhan, M.; Ngari, M.S.; Leonard, O.P. Screening for antimicrobial activity of *Cissampelos pareira* L. methanol root extract. *Eur. J. Med. Plants* **2014**, *4*, 45.

232. Ayepola, O.O.; Samson, A.F.; Onile-Ere, O. In vitro Antioxidant and Anti-staphylococcal Activity of *Bixa orellana* Linn. and Milicia excelsa Welw. *J. Altern. Complement. Med.* **2018**, *5*.
233. Nkya, J.W.; Erasto, P.; Chacha, M. Antimycobacterial and cytotoxicity activities of *Moringa oleifera* Lam extracts. *Am. J. Res. Commun.* **2014**, *2*, 108–120.
234. Abdallah, E.M. Antibacterial properties of leaf extracts of *Moringa oleifera* Lam. growing in Sudan. *J. Adv. Med. Pharm. Sci.* **2016**, *5*, 1–5.
235. Patel, N.; Mohan, J. Antimicrobial activity and phytochemical analysis of *Moringa oleifera* Lam. crude extracts against selected bacterial and fungal strains. *Int. J. Pharmacogn. Phytochem. Res.* **2018**, *10*, 68–79.
236. Marrufo, T.; Nazzaro, F.; Mancini, E.; Fratianni, F.; Coppola, R.; De Martino, L.; Agostinho, A.B.; De Feo, V. Chemical composition and biological activity of the essential oil from leaves of *Moringa oleifera* Lam. cultivated in Mozambique. *Molecules* **2013**, *18*, 10989–11000.
237. Bukar, A.; Uba, A.; Oyeyi, T. Antimicrobial profile of *Moringa oleifera* Lam. extracts against some food–borne microorganisms. *Bayero J. Pure Appl. Sci.* **2010**, *3*, 43–48.
238. Pallant, C.; Steenkamp, V. In-vitro bioactivity of Venda medicinal plants used in the treatment of respiratory conditions. *Hum. Exp. Toxicol.* **2008**, *27*, 859–866.
239. Boga Gogo, L.; Djaman Allico, J. In vitro evaluation of the activity of aqueous extract of a plant coded phyllam (*Phyllanthus amarus*) on two strains of *Mycobacterum tuberculosis* and one strain of *Mycobacterum ulcerans*. *Skin* **2013**, *11*, 13.
240. Temitope, O.O.; Ajayi Ayodele, O. Antimicrobial, phytochemical and proximate analysis of four Nigerian

medicinal plants on some clinical microorganisms. *Curr. Res. Microbiol. Biotechnol.* **2014**, *2*, 457–461.
241. Cimanga Kanyanga, R.; Malika Bool-Miting, F.; Tona Lutete, G.; Kambu Kabangu, O.; Vlietinck, A.; Pieters, L. Antibacterial screening of aqueous extracts of some medicinal plant and their fractions used as antidiarrheal agents in Kinshasa-Democratic Republic of Congo. *World J. Pharm. Pharm. Sci.* **2018**, *7*, 223–242.
242. Joseph, C.; Moshi, M.; Sempombe, J.; Nkunya, M. (4-methoxy-benzo [1, 3] dioxol-5-yl)-phenylmethanone: An antibacterial benzophenone from *Securidaca longepedunculata*. *Afr. J. Tradit. Complement. Altern. Med.* **2006**, *3*, 80–86.
243. Tiksa, T.; Abdissa, D.; Abdissa, N. Chemical constituents of *Securidaca longipedunculata* root bark and evaluation of their antibacterial activities. *Ethiop. J. Educ. Sci.* **2019**, *14*, 1–8.
244. Mostafa, M.; Afolayan, A. Antimicrobial activity of *Clematis brachiata* Thunb leaf extracts. *Int. J. Pharm. Sci.* **2013**, *4*, 243.
245. Molla, Y.; Nedi, T.; Tadesse, G.; Alemayehu, H.; Shibeshi, W. Evaluation of the in vitro antibacterial activity of the solvent fractions of the leaves of *Rhamnus prinoides* L'Herit (Rhamnaceae) against pathogenic bacteria. *BMC Complement. Altern. Med.* **2016**, *16*, 1–9.
246. Jadhav, N.; Kulkarni, S.; Mane, A.; Kulkarni, R.; Palshetker, A.; Singh, K.; Joshi, S.; Risbud, A.; Kulkarni, S. Antimicrobial activity of plant extracts against sexually transmitted pathogens. *Nat. Prod. Res.* **2015**, *29*, 1562–1566.
247. Kritheka, N.; Kumar, R.S.; Kumar, S.S.; Murthy, N.V.; Sundram, R.S.; Perumal, P. Anti-inflammatory and Antimicrobial activities of Petroleum ether and Ethanol extracts of *Scutia myrtina* (Rhamnaceae). *Adv. Tradit. Med.* **2008**, *8*, 400–407.
248. Nyaberi, M.; Onyango, C.; Mathooko, F.; Maina, J.; Makobe, M.; Mwaura, F. Evaluation of phytochemical, antioxidant and antibacterial activity of edible fruit extracts of *Ziziphus abyssinica* A. Rich. *J. Anim. Plant Sci.* **2010**, *6*, 623–629.
249. Mustapha, Y.; Bala, M. Antimicrobial activity of leaf extracts of *Pavetta crassipes* (hutch) against some respiratory tract pathogens. *Sci. World J.* **2007**, *2*.
250. Odumosu, P.O.; Lough, W.J.; Davis Yakubu, K.T.; Williamson, G.; Haroune, N. Anti-mycobacterial assessment and characterization of 5-O-caffeoylquinic acid methyl ester and rutin from *Pavetta crassipes*. *J. Appl. Pharm. Sci.* **2016**, *6*, 001–007.
251. Man, A.; Santacroce, L.; Iacob, R.; Mare, A.; Man, L. Antimicrobial activity of six essential oils against a group of human pathogens: A comparative study. *Pathogens* **2019**, *8*, 15.
252. Mehmood, T.; Afzal, A.; Anwar, F.; Iqbal, M.; Afzal, M.; Qadir, R. Variations in the composition, antibacterial and haemolytic activities of peel essential oils from unripe and ripened *Citrus limon* (L.) Osbeck fruit. *J. Essent. Oil Bear. Plants* **2019**, *22*, 159–168.
253. Saeb, S.; Amin, M.; Gooybari, R.S.; Aghel, N. Evaluation of antibacterial activities of *Citrus limon*, *Citrus reticulata*, and *Citrus grandis* against pathogenic bacteria. *Int. J. Enteric. Pathog.* **2016**, *4*, 3–37103.
254. Kirbaşlar, F.G.; Tavman, A.; Dülger, B.; Türker, G. Antimicrobial activity of Turkish citrus peel oils. *Pak. J. Bot.* **2009**, *41*, 3207–3212.
255. Ngbolua, K.; Tshibangu, D.; Mpiana, P.; Mihigo, S.; Mavakala, B.; Ashande, M.; Muanyishay, L. Anti-sickling and antibacterial activities of Some Extracts from *Gardenia ternifolia* subsp. *jovis-tonantis* (Welw.) Verdc.(Rubiaceae) and *Uapaca heudelotii* Baill.(Phyllanthaceae). *Infection* **2015**, *4*, 6.
256. Mayaka, R.K.; Langat, M.K.; Omolo, J.O.; Cheplogoi, P.K. Antimicrobial prenylated acetophenones from berries of *Harrisonia abyssinica*. *Planta Med.* **2012**, *78*, 383–386.
257. Gakuubi, M.M.; Micheni, K.N.; Wanzala, W. In vitro Antibacterial Activity of Essential Oil from the Fruits of *Toddalia asiatica* (L) Lam.(Rutaceae). *J. Biol. Act. Prod. Nat* **2017**, *7*, 52–61.
258. He, N.; Wang, P.; Wang, P.; Ma, C.; Kang, W. Antibacterial mechanism of chelerythrine isolated from root of *Toddalia asiatica* (Linn) Lam. *BMC Complement. Altern. Med.* **2018**, *18*, 1–9.
259. Shi, L.; Ji, Z.-q.; Li, Y.-m.; Li, Y.-y.; Guo, C.-s. Antioxidant and antibacterial activity of *Toddalia asiatica* (Linn) Lam root extracts. *Afr. J. Tradit Complement. Altern. Med.* **2015**, *12*, 169–179.
260. Karthikeyan, A.; Sivasaravanan, K.; Gnanavel, I.; Prakash, D.; Velpandian, V. Screening of anti microbial activity of Siddha herbal drug Milagaranai Ver Chooranam (root of *Toddalia asiatica*). *Int. J. Res. Ayurveda Pharm.* **2014**, *5*, 347–351.

261. Duraipandiyan, V.; Ignacimuthu, S. Antibacterial and antifungal activity of Flindersine isolated from the traditional medicinal plant, Toddalia asiatica (L.) Lam. *J. Ethnopharmacol.* **2009**, *123*, 494–498.
262. Chrian, M.; Erasto, P.; Otieno, J. Antimycobacterial activity and cytotoxicity effect of extracts of *Hallea rubrostipulata* and *Zanthoxylum chalybeum*. *Spatula DD* **2011**, *1*, 147.
263. Jelager, L.; Gurib-Fakim, A.; Adsersen, A. Antibacterial and antifungal activity of medicinal plants of Mauritius. *Pharm. Biol.* **1998**, *36*, 153–161.
264. Barreto, F.; Sousa, E.; Rodrigues, F.; Costa, J.; Campos, A. Antibacterial activity of *Lantana camara* linn *lantana montevidensis* brig extracts from cariri-ceara, Brazil. *J. Young Pharm* .**2010**, *2*, 42-44.
265. Naz, R.; Bano, A. Phytochemical screening, antioxidants and antimicrobial potential of *Lantana camara* in different solvents. *Asian Pac. J. Trop. Dis.* **2013**, *3*, 480–486.
266. Eldeen, I.; Van Staden, J. Cyclooxygenase inhibition and antimycobacterial effects of extracts from Sudanese medicinal plants. *S. Afr. J. Bot.* **2008**, *74*, 225–229.
267. Mwangi, H.M.; Mabusela, W.T.; Abegaz, B.M.; Martin, O.O. Antimicrobial activities of a novel biflavonoid and other constituents from *Rhus natalensis*. *J. Med. Plant Res.* **2013**, *7*, 619–623.
268. Njoroge, P.W.; Opiyo, S.A. Some antibacterial and antifungal compounds from root bark of *Rhus natalensis*. *Am. J. Chem.* **2019**, *9*, 150–158.
269. Lall, N.; Kishore, N.; Bodiba, D.; More, G.; Tshikalange, E.; Kikuchi, H.; Oshima, Y. Alkaloids from aerial parts of *Annona senegalensis* against *Streptococcus mutans*. *Nat. Prod. Res.* **2017**, *31*, 1944–1947.
270. Okoye, T.C.; Akah, P.A.; Okoli, C.O.; Ezike, A.C.; Omeje, E.O.; Odoh, U.E. Antimicrobial effects of a lipophilic fraction and kaurenoic acid isolated from the root bark extracts of *Annona senegalensis*. *Evid. Based Complement. Altern.* **2012**, *2012*.
271. Ebeh Messanga, R.; Dominique Serge, N.B.; Abouem, A.; Zintchem, A.; Norbert, M.N.I.; Esther Del Florence, M.N.; Patrick Hervé, B.D.; Maximilienne Ascension, N.; Alex De Théodore, A.; Dieudonné Emmanuel, P.; et al. Rauvolfianine, a new antimycobacterial glyceroglycolipid and other constituents from *Rauvolfia caffra*. Sond (Apocynaceae). *Nat. Prod. Res.* **2018**, *32*, 1971–1976.
272. Binutu, O.A.; Adesogan, K.E.; Okogun, J.I. Antibacterial and antifungal compounds from *Kigelia pinnata*. *Planta Med.* **1996**, *62*, 352–353.
273. Hanwa, U.A.; Tijjani, M.A.; Sani, Y.M.; Atiku, I.; Sule, M.I.; Hassan, H.S.; Pateh, U.U. Antimicrobial activity of ipolamiide isolated from the stembark of *Stereospermum kunthianum* Cham (Bignoniaceae). *JPB* **2017**, *14*, 150–154.
274. Kamau, R.W.; Midiwo, J.O.; Mgani, Q.A.; Masila, V.M.; Omosa, L.K.; Bwire, R.N.; Jacob, M.R.; Wiggers, F.T.; Muhammad, I. Oleanolic Acid and other Compounds Isolated from *Cordia africana* Lam which Inhibit Vancomycin Resistant Enterococcus. *Pharmacogn. Commun.* **2019**, *9*, 91–95.
275. Opiyo, S.A.; Manguro, L.O.; Okinda-Owuor, P.; Ateka, E.M.; Lemmen, P. 7α-Acetylugandensolide and antimicrobial properties of *Warburgia ugandensis* extracts and isolates against sweet potato pathogens. *Phytochem. Lett.* **2011**, *4*, 161–165.
276. Mushi, N.F.; Innocent, E.; Kidukuli, A.W. Cytotoxic and antimicrobial activities of substituted phenanthrenes from the roots of *Combretum adenogonium* Steud Ex A. Rich (Combretaceae). *J. Intercult. Ethnopharmacol.* **2015**, *4*, 52.
277. Rücker, G.; Kehrbaum, S.; Sakulas, H.; Lawong, B.; Goeltenboth, F. Acetylenic Glucosides from *Microglossa pyrifolia*. *Planta Med.* **1992**, *58*, 266–269.
278. Rabe, T.; Mullholland, D.; Van Staden, J. Isolation and identification of antibacterial compounds from *Vernonia colorata* leaves. *J. Ethnopharmacol.* **2002**, *80*, 91–94.
279. Adu, J.K.; Twum, K.; Brobbey, A.; Amengor, C.; Duah, Y. Resistance modulation studies of vernolide from *Vernonia colorata* (Drake) on ciprofloxacin, amoxicillin, tetracycline and erythromycin. *J. Phytopharm.* **2018**, *7*, 425–430.
280. Okwu, D.E.; Nnamdi, F.U. Two novel flavonoids from *Bryophyllum pinnatum* and their antimicrobial activity. *J. Chem. Pharm. Res* **2011**, *3*, 1–10.
281. Ebere Okwu, D.; Uchenna Nnamdi, F. A novel antimicrobial phenanthrene alkaloid from *Bryopyllum pinnatum*. *Electron. J. Chem.* **2011**, *8*, 1456–1461.
282. San Francisco, M.; Cooper-Driver, G. Anti-microbial activity of phenolic acids in *Pteridium aquilinum*. *Am. Fern J.* **1984**, *74*, 87–96.
283. Mohammad, R.H. Pterosins and Pterosides in Bracken (*Pteridium aquilinum* (L.) Kuhn). Ph.D. Thesis, Bangor University, Bangor, United Kingdom, 2016.
284. Perumal, S.; Mahmud, R.; Ramanathan, S. Anti-infective potential of caffeic acid and epicatechin 3-gallate

isolated from methanol extract of *Euphorbia hirta* (L.) against *Pseudomonas aeruginosa*. *Nat. Prod. Res.* **2015**, *29*, 1766–1769.
285. Mujovo, S.F.; Hussein, A.A.; Meyer, J.J.; Fourie, B.; Muthivhi, T.; Lall, N. Bioactive compounds from *Lippia javanica* and H*oslundia opposita*. *Nat. Prod. Res.* **2008**, *22*, 1047–1054.
286. Pereda-Miranda, R.; Hernández, L.; Villavicencio, M.J.; Novelo, M.; Ibarra, P.; Chai, H.; Pezzuto, J.M. Structure and stereochemistry of pectinolides AC, novel antimicrobial and cytotoxic 5, 6-dihydro-α-pyrones from *Hyptis pectinata*. *J. Nat. Prod.* **1993**, *56*, 583–593.
287. Fragoso-Serrano, M.; Gibbons, S.; Pereda-Miranda, R. Anti-staphylococcal and cytotoxic compounds from *Hyptis pectinata*. *Planta Med.* **2005**, *71*, 278–280.
288. Limmatvapirat, C.; Sirisopanaporn, S.; Kittakoop, P. Antitubercular and antiplasmodial constituents of *Abrus precatorius*. *Planta Med.* **2004**, *70*, 276–278.
289. Mutai, C.; Bii, C.; Vagias, C.; Abatis, D.; Roussis, V. Antimicrobial activity of *Acacia mellifera* extracts and lupane triterpenes. *J. Ethnopharmacol.* **2009**, *123*, 143–148.
290. Mutai, C.; Bii, C.; Rukunga, G.; Ondicho, J.; Mwitari, P.; Abatis, D.; Vagias, C.; Roussis, V.; Kirui, J. Antimicrobial activity of pentacyclic triterpenes isolated from *Acacia mellifera*. *Afr. J. Tradit. Complement. Altern. Med.* **2009**, *6,* 57072.

291. Endo, M.; Naoki, H. Antimicrobial and antispasmodic tetrahydroanthracenes from *Cassia singueana*. *Tetrahedron* **1980**, *36*, 2449–2452.
292. Banso, A.; Adeyemo, S. Evaluation of antibacterial properties of tannins isolated from *Dichrostachys cinerea*. *Afr. J. Biotechnol.* **2007**, *6*, 1785-87.
293. Stanley, I. Isolation and Characterization of Antimicrobial Compounds from the Plants, *Erythrina abyssinica* dc. and *Chasmanthera dependens* HOCHST. Master's Thesis, Kenyata University, Nairobi, Kenya, 2012.
294. Biftu, A.; Adane, L.; Tariku, Y. Evaluation of antibacterial activities of compounds isolated from fruits of *Sida rhombifolia* Linn. *Middle East J. Sci. Res.* **2014**, *22*, 681–689.
295. Mbaveng, A.T.; Sandjo, L.P.; Tankeo, S.B.; Ndifor, A.R.; Pantaleon, A.; Nagdjui, B.T.; Kuete, V. Antibacterial activity of nineteen selected natural products against multi-drug resistant Gram-negative phenotypes. *SpringerPlus* **2015**, *4*, 1–9.
296. Oluduro, O.; Aderiye, B.; Connolly, J.; Akintayo, E.; Famurewa, O. Characterization and antimicrobial activity of 4-(β-D-glucopyranosyl-1→ 4-α-L-rhamnopyranosyloxy)-benzyl thiocarboxamide; a novel bioactive compound from *Moringa oleifera* seed extract. *Folia Microbiol.* **2010**, *55*, 422–426.
297. Eilert, U.; Wolters, B.; Nahrstedt, A. The antibiotic principle of seeds of *Moringa oleifera* and *Moringa stenopetala*. *Planta Med.* **1981**, *42*, 55–61.
298. Padla, E.P.; Solis, L.T.; Levida, R.M.; Shen, C.-C.; Ragasa, C.Y. Antimicrobial isothiocyanates from the seeds of *Moringa oleifera* Lam. *Z. Nat. C* **2012**, *67*, 557–564.
299. Magadula, J.J.; Mwangomo, D.T.; Moshi, M.J.; Heydenreich, M. A novel γ-lactone and other constituents of a Tanzanian *Antidesma venosum*. *Spatula DD Peer Rev. J. Complement. Med. Drug Dis.* **2013**, *3*, 7À12.
300. Ekuadzi, E.; Dickson, R.; Fleischer, T.; Annan, K.; Pistorius, D.; Oberer, L.; Gibbons, S. Flavonoid glycosides from the stem bark of *Margaritaria discoidea* demonstrate antibacterial and free radical scavenging activities. *Phytother. Res.* **2014**, *28*, 784–787.
301. Chouna, J.R.; Nkeng-Efouet-Alango, P.; Lenta, B.N.; Sewald, N. Antimicrobial triterpenes from the stem bark of *Crossopteryx febrifuga*. *Z. Nat. C* **2015**, *70*, 169–173.
302. Bello, I.A.; Ndukwe, G.I.; Audu, O.T.; Habila, J.D. A bioactive flavonoid from *Pavetta crassipes* K. Schum. *Org. Med. Chem. Lett.* **2011**, *1*, 1–5.
303. Ibekwe, N.N.; Boshoff, H.; Randle, J.; Adesomoju, A.A.; Barry III, C.E.; Okogun, J.I. Chemical constituents and antimycobacterial studies of the leaf extracts of *Pavetta crassipes* K. Schum. *Trop. Plant Res.* **2018**, *5*, 88–95.
304. Raj, M.K.; Balachandran, C.; Duraipandiyan, V.; Agastian, P.; Ignacimuthu, S. Antimicrobial activity of Ulopterol isolated from *Toddalia asiatica* (L.) Lam.: A traditional medicinal plant. *J. Ethnopharmacol.* **2012**, *140*, 161–165.
305. Phatchana, R.; Yenjai, C. Cytotoxic coumarins from *Toddalia asiatica*. *Planta Med.* **2014**, *80*, 719–722.
306. Hu, J.; Shi, X.; Chen, J.; Mao, X.; Zhu, L.; Yu, L.; Shi, J. Alkaloids from *Toddalia asiatica* and their cytotoxic, antimicrobial and antifungal activities. *Food Chem.* **2014**, *148*, 437–444.
307. Begum, S.; Wahab, A.; Siddiqui, B. Antimycobacterial activity of flavonoids from *Lantana camara* Linn. *Nat. Prod. Res.* **2008**, *22*, 467–470.
308. Induli, M.; Cheloti, M.; Wasuna, A.; Wekesa, I.; Wanjohi, J.M.; Byamukama, R.; Heydenrich, M.; Makayoto, M.;

Yenesew, A. Naphthoquinones from the roots of *Aloe secundiflora*. *Phytochem. Lett.* **2012**, *5*, 506–509.
